# Multifunctional hydrogel nano-probes for atomic force microscopy

**DOI:** 10.1038/ncomms11566

**Published:** 2016-05-20

**Authors:** Jae Seol Lee, Jungki Song, Seong Oh Kim, Seokbeom Kim, Wooju Lee, Joshua A. Jackman, Dongchoul Kim, Nam-Joon Cho, Jungchul Lee

**Affiliations:** 1 Department of Mechanical Engineering, Sogang University, 35 Baekbeom-ro (Sinsu-dong), Mapo-gu, Seoul 04107, South Korea; 2 School of Materials Science and Engineering and Centre for Biomimetic Sensor Science, Nanyang Technological University, 50 Nanyang Drive, Singapore 637553, Singapore; 3 School of Chemical and Biomedical Engineering, Nanyang Technological University, 62 Nanyang Drive, Singapore 637459, Singapore

## Abstract

Since the invention of the atomic force microscope (AFM) three decades ago, there have been numerous advances in its measurement capabilities. Curiously, throughout these developments, the fundamental nature of the force-sensing probe—the key actuating element—has remained largely unchanged. It is produced by long-established microfabrication etching strategies and typically composed of silicon-based materials. Here, we report a new class of photopolymerizable hydrogel nano-probes that are produced by bottom-up fabrication with compressible replica moulding. The hydrogel probes demonstrate excellent capabilities for AFM imaging and force measurement applications while enabling programmable, multifunctional capabilities based on compositionally adjustable mechanical properties and facile encapsulation of various nanomaterials. Taken together, the simple, fast and affordable manufacturing route and multifunctional capabilities of hydrogel AFM nano-probes highlight the potential of soft matter mechanical transducers in nanotechnology applications. The fabrication scheme can also be readily utilized to prepare hydrogel cantilevers, including in parallel arrays, for nanomechanical sensor devices.

## Abstract (web summary)

Atomic force microscopy typically employs hard tips to map the surface topology of a sample, with sub-nanometre resolution. Here, the authors instead develop softer hydrogel probes, which show potential for multifunctional measurement capabilities beyond that of conventional systems.

## Graphical abstract

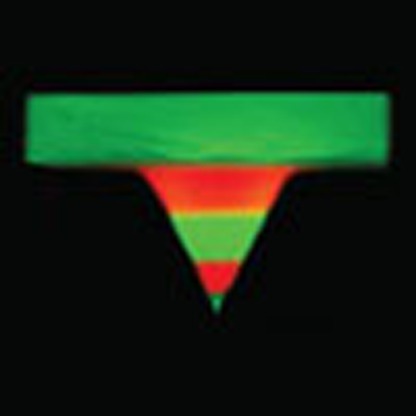

A compelling motivation in surface science is the development of nanoscale probes to seamlessly interrogate the molecular-level properties of material interfaces. This goal envisions probes which both engage and respond to the local material environment. To this end, scanning probe microscopy^1^,^2^ techniques have revolutionized our experimental capabilities for surface metrology. Atomic force microscopy (AFM)^3^ is one of the most successful scanning probe microscopy techniques and employs a cantilever-mounted tip to probe atomic details of a surface. When the tip approaches a surface, the cantilever deflection is influenced by atomic interactions between the tip and sample. Depending on the application and sample properties, the AFM probe design can be varied for optimal sensing—common parameters to adjust include mechanical properties of the cantilever such as spring constant^4^ and resonance frequency^5^ as well as the tip geometry^6^. In the case of conventional silicon-based probes, mechanical characteristics of the probe are mainly controlled by geometrical dimensions of the cantilever. Indeed, there is a narrow tuning range of the elastic modulus, and tip geometries are typically confined to cones or pyramids with fixed aspect ratios stemming from limited recipes for materials etching. Another option is to functionalize the tip to improve imaging performance or enable a specific application. In such cases, the tip can be treated with a covalent surface modification (for example, functional groups by silane or thiol chemistry^7^), which have a high-aspect ratio nanomaterial (for example, carbon nanotube^8^) attached to the tip apex, or be fabricated from an unconventional material (for example, hydrophobic acrylate and epoxy blend^9^ or SU8 photoplastic^10^). Silicon-based probes remain the standard technology in the field for both the cantilever and tip components due to advanced fabrication capabilities, including tip designs with small radii of curvature for high-resolution performance. At the same time, the current emphasis on silicon-based technologies has limited efforts to discover new promising materials or fabrication methods for further innovating AFM probes.

With a growing range of AFM nanotechnology applications^11^,^12^, the need for developing multifunctional AFM probes is paramount, motivating the exploration of new material compositions and fabrication strategies^13^. In particular, there is significant opportunity to develop AFM probes that go beyond two-dimensional surface functionalization and have three-dimensional (3D) programmable features with compositionally tunable properties. To realize this goal, molecular self-assembly offers key advantages over conventional microfabrication for materials programming, that is, imparting functionality through modular combinations of polymeric, organic and inorganic nanomaterials. Photopolymerizable hydrogels are an excellent class of candidate material with tunable mechanical properties^14^,^15^, vast functionalization possibilities^16^,^17^,^18^ and flexibility to encapsulate nanomaterials of varying size^19^,^20^. Furthermore, the soft and compliant nature of hydrogels could be useful for soft matter and biological AFM applications, for which sample wear and damage is a major challenge^21^. Hence, hydrogels have strong merits to be explored as a material for fabricating AFM probes.

Realizing the potential of hydrogel composites as nanoscale actuating probes for AFM applications^5^,^22^,^23^ would also represent a significant technical advance for nanomechanical sensors in general. The functional features of AFM probes inspired the creation of the field of cantilever-based nanomechanical sensing, which involves highly sensitive detection of biological and chemical analytes among other application possibilities^24^,^25^. As with AFM probes, tipless cantilever sensors are typically fabricated from silicon-based materials; however, there has been strong interest in exploring polymeric materials^26^,^27^ with lower mechanical stiffness as an alternative, often superior option for high-sensitivity detection^25^,^28^. In some cases, nanoparticles have been incorporated into the polymeric cantilevers for multifunctional applications^29^. To date, the cantilevers have been composed of relatively stiff, hydrophobic polymers, while softer hydrogels have been explored as a surface coating option to improve the stability and reusability of SU8 polymeric cantilevers^30^ as well as for ion sensing^31^. Hydrogel-based cantilevers with lower, and widely tunable, mechanical stiffnesses could enable more sensitive detection capabilities while also providing an improved tool for nanomechanical measurements on soft matter systems. These features would be especially advantageous if a simple and reproducible fabrication scheme could be employed for producing the hydrogel cantilevers. Taken together, all these points highlight the potential significance of developing hydrogel-based cantilevers, both in the context of fully integrated AFM probes as well as for cantilever-based nanomechanical sensor devices.

Towards this goal, we explore the design, fabrication and testing of multifunctional hydrogel AFM probes. From a fabrication perspective, the design of hydrogel AFM probes represents a particularly challenging feature because it involves manufacturing and integrating hydrogel cantilevers and tips—the nanoscale geometrical features of the latter are also important. The learnings from work in this direction can be directly applied to fabricating tipless cantilevers as well. Experimentally, our findings indicate that hydrogel AFM probes enable stable and reproducible force, and imaging measurements of various inorganic and biological samples in air and liquid conditions. The combination of fabrication possibilities and multifunctional versatility afforded by the hydrogel probes offers the first demonstration of how 3D materials design strategies can be utilized for AFM applications, thereby showing the potential of soft matter to provide superior and versatile actuators for surface metrology. The hydrogel nano-probes developed in this work have the potential to be extended to cantilever-based nanomechanical sensing applications in both single measurement and array configurations.

## Results

### Fabrication of hydrogel AFM probes

**Figure 1:**
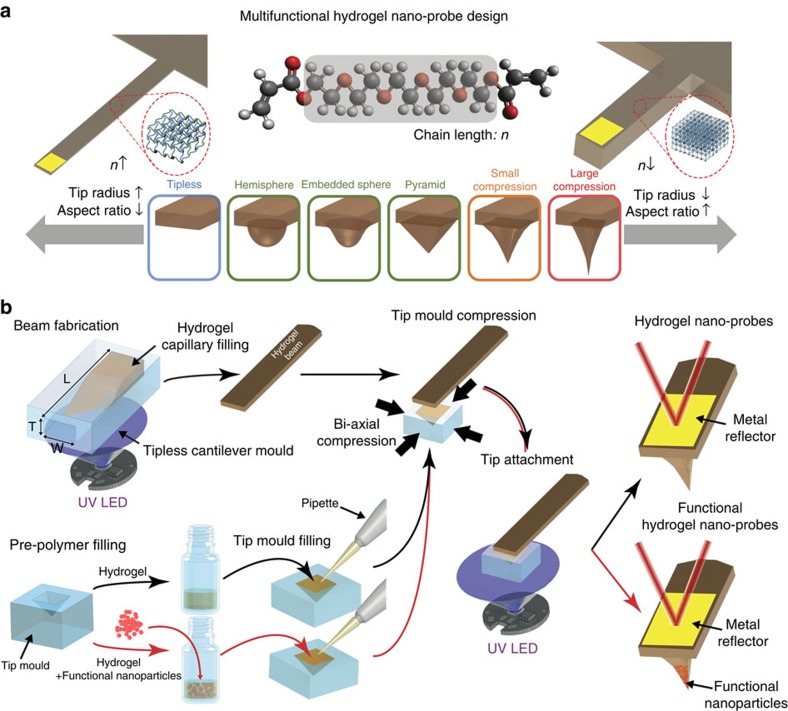
Design and fabrication of hydrogel AFM nano-probes. (**a**) Conceptual schematic for beam and tip geometry to tune probe characteristics for various AFM applications. Capillary driven filling of ultraviolet curable hydrogels into the engineered beam mould facilitates fabrication of tipless hydrogel cantilevers with tunable spring constants over several orders of magnitude by varying the MW of the monomer composition and geometrical dimensions of the cantilever. Nanoscale dimensions of the tip shape can also be tuned by preparing different tip moulds or by applying deformation to a given mould. When the tip mould is compressed, the tip becomes sharper and the tip aspect ratio typically increases. (**b**) Fabrication method for hydrogel AFM probes. A tipless hydrogel cantilever is first prepared by ultraviolet curing of the pre-polymer solution introduced into the cantilever beam mould. The tipless hydrogel cantilever then makes contact with a tip mould filled with pre-polymer solution without or with encapsulated functional elements, followed by a second round of ultraviolet exposure which cures the hydrogel in the tip mould and results in firm attachment between the cantilever and tip. Before the second ultraviolet exposure, the hydrogel-filled tip mould can be optionally deformed by applying bi-axial compressive strains to facilitate tunable tip sharpness and aspect ratio.

Hydrogel materials are readily fabricated by a molecular self-assembly process that involves light-sensitive polymerization reactions^32^. A wide variety of materials can form hydrogels and an even greater range of materials can be encapsulated within hydrogels^33^. While hydrogels are routinely fabricated on the microscale, hydrogel fabrication on the nanoscale requires a delicate approach based on high precision tuning of the tip dimensions to enable various scanning probe applications (Fig. 1a). To fabricate hydrogel AFM probes, we took advantage of a bottom-up strategy that uses aqueous conditions, which contrasts with the typical etching of silicon probes that involves harsh treatments and organic solvents^34^. As presented in Fig. 1b, the strategy involves fabrication of the tipless hydrogel cantilever, which is first prepared by ultraviolet light-induced curing of a pre-polymer solution introduced into the cantilever beam mould. The tipless hydrogel cantilever then makes contact with a tip mould filled with pre-polymer solution, followed by a second round of ultraviolet light exposure which cures the hydrogel in the tip mould and results in firm attachment between the cantilever and tip. Before the second ultraviolet light exposure, the hydrogel-filled tip mould can be optionally deformed to apply compressive strains which facilitate tunable tip sharpness and aspect ratio. After tip fabrication, a metal reflective coating is then added onto the top portion of the cantilever.

**Figure 2:**
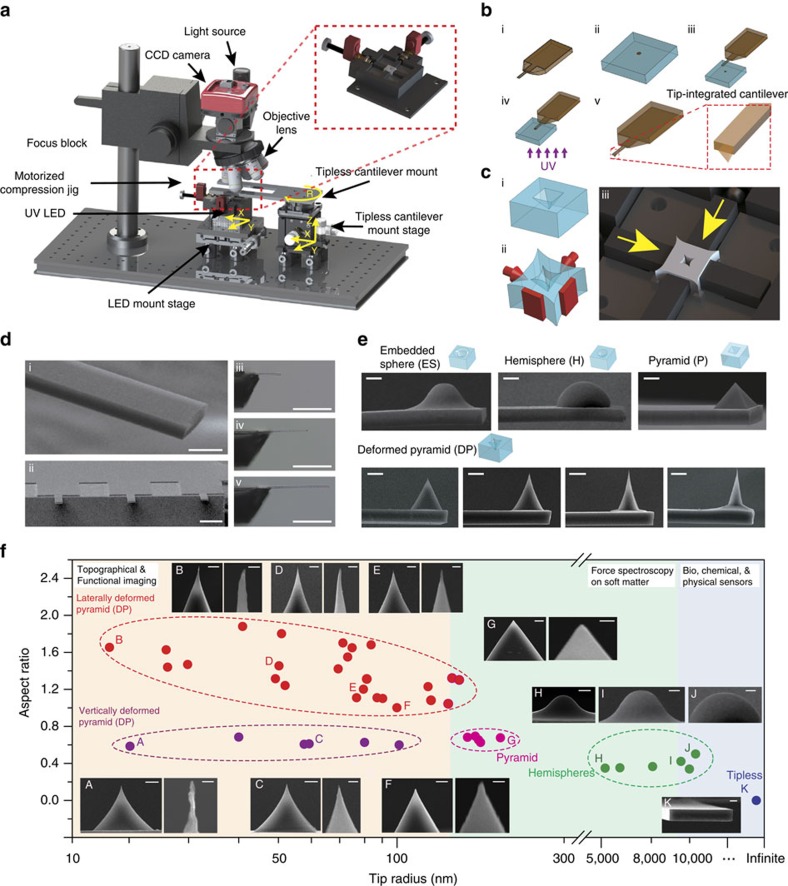
Replica moudling strategy to fabricate hydrogel nano-probes. (**a**) Experimental set-up for fabricating hydrogel nano-probes, which consists of a custom-built microscope, a ultraviolet light-emitting diode (LED), and a compression jig for tip shape tuning. (**b**) Tip integration process (i) Fabricated tipless hydrogel cantilever, (ii) Fill the pre-polymer solution into a tip mould (in this case, an inverted pyramid although the design can vary), (iii) Approach the tipless hydrogel cantilever towards the hydrogel-filled tip mould and make contact between the cantilever and tip mould, (iv) Cure hydrogel tip with ultraviolet light, (v) Separate the tip-integrated hydrogel cantilever from tip mould). (**c**) Tip shape tuning via compression replica moudling (i) PDMS pyramidal tip mould, (ii) bi-axial compression to the tip mould and 3D schematic (iii) for bi-axial compression of the tip mould. (**d**) Scanning electron and optical micrographs of a single tipless hydrogel cantilever (i) and its array (ii). Tipless hydrogel cantilevers with lengths of (iii) 300, (iv) 500 and (v) 700 μm, respectively. Scale bars are 20, 200 and 500 μm, for (i), (ii) and (iii–v), respectively. (**e**) Scanning electron micrographs showing different tips integrated on hydrogel cantilevers—embedded sphere (ES), hemisphere (H), pyramid (P) and deformed pyramid (DP). All scale bars are 10 μm. (**f**) Summary of tip radii and aspect ratios of various deformed pyramidal tips. Scale bars are 5 μm for overall views, 200 nm for the zoom-ins of A and B, and 1 μm for the other zoom-ins (C–G).

The detailed fabrication set-up is presented in Fig. 2a. A hydrogel cantilever was fabricated by using a polydimethylsiloxane (PDMS) replica mould inside a curing set-up (Supplementary Fig. 1). The pre-polymer solution containing poly(ethylene glycol) diacrylate (PEG-DA; molecular weight (MW) of 250 g mol^−1^) was filled into the mould and the solution was cured by applying ultraviolet light (Supplementary Fig. 1). To functionalize the hydrogel cantilever with a tip, simultaneous hydrogel tip fabrication and attachment was performed (Fig. 2b). The steps entailed filling a tip mould with PEG-DA pre-polymer solution, contacting the cantilever base with the tip mould, and applying ultraviolet light to cure the hydrogel tip (Fig. 2b and Supplementary Figs 2 and 3). This last step cross-linked the PEG-DA monomers in the tip mould and also led to cross-linking between PEG-DA monomers in the tip and cantilever base. Hence, an integrated hydrogel AFM probe was created that possesses a cantilever base and tip. Control of the tip geometry and sharpness was established by using a compression jig that is included in the fabrication set-up (Fig. 2c). Sequential uniaxial and bi-axial compression enables precise tip shapes to be formed. The resulting hydrogel cantilevers had controllable length and exhibited flatness in ambient conditions (Fig. 2d). Representative scanning electron microscopy images further demonstrate the fabrication of hydrogel tips with various shapes, including embedded spheres, hemispheres and pyramids (Fig. 2e). For pyramidal tips, the tip radius was estimated by circular fitting of the tip apex region (Supplementary Fig. 4). Importantly, hydrogel tips with sub 20 nm radii were achievable with the compressible replica moudling approach. Taken together, the characterization studies indicate that the tip radius and aspect ratio can be precisely tuned for different applications (Fig. 2f and Supplementary Figs 5 and 6). As PEG-DA hydrogels are optically transparent, the detector side of the cantilever was partially covered by a reflective metal (Ti/Au; 20/100 nm) coating for AFM operation (Supplementary Fig. 7) and the cantilevers remained flat in air and water (Supplementary Fig. 8). For more precise registration of the tip, the area and position of the metal coating can be adjusted to allow the scanning tip to be visually accessible (Supplementary Fig. 9).

### Tunable mechanical force sensing

**Figure 3:**
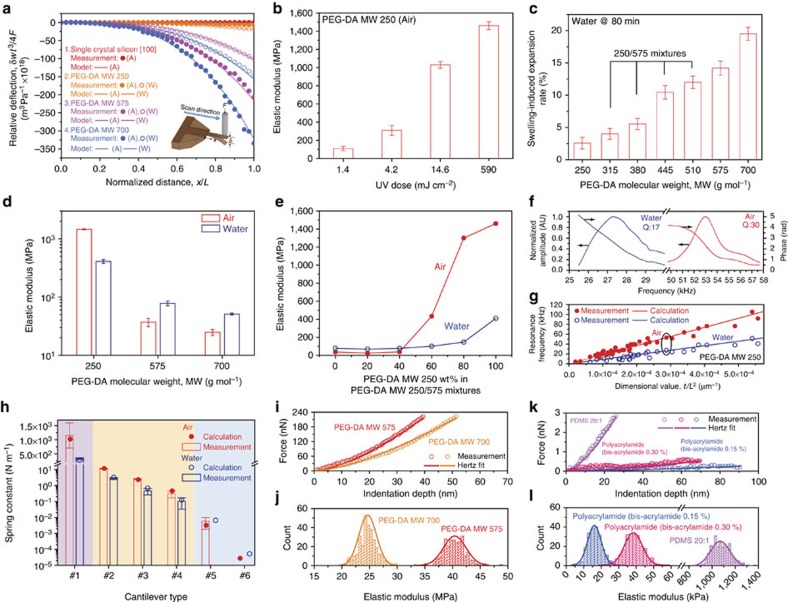
Tunable mechanical properties of hydrogel nano-probes for force-sensing applications. (**a**) Relative deflection of hydrogel cantilevers as a function of normalized displacement. ‘A' and ‘W' represent air and water, respectively. (**b**) Elastic moduli of PEG-DA MW 250 hydrogel cured under different ultraviolet doses. Error bars represent s.d. of the mean with *N*=3 measurements. (**c**) Swelling-induced expansion of PEG-DA hydrogels in water. (**d**) Elastic moduli of PEG-DA MW 250, 575 and 700 in air and water. Error bars represent s.d. of the mean with *N*=3 measurements. (**e**) Elastic moduli of PEG-DA MW 250/575 mixtures in air and water as a function of wt% of PEG-DA MW 250. (**f**) Resonance spectra of a hydrogel cantilever in air and water (length, width and thickness: 222, 50 and 15 μm). (**g**) Resonance frequencies of various hydrogel cantilevers with different dimensions in air and water. The two data points enclosed by the ellipse correspond to the measurement values for the hydrogel cantilever shown in **f**. A total of 53 and 35 measurements were completed in air and water, respectively. (**h**) Experimental and theoretical spring constant values for hydrogel cantilevers in air and water (length, width and thickness: 500, 350 and 100 μm (#1); 220, 50 and 19 μm (#2); 400, 50 and 20 μm (#3); 375, 50 and 11 μm (#4); 505, 50 and 11 μm (#5); and 1500, 35 and 5 μm (#6)). Cantilevers #1–4 were fabricated using PEG-DA MW 250, and cantilevers #5 and #6 were fabricated using PEG-DA MW 700. Error bars represent s.d. of the mean with *N*=3 measurements. (**i**) Force-indentation curves on PEG-DA hydrogel slabs in air with Hertzian curve fits. (**j**) Elastic moduli of different MW PEG-DA samples in air. (**k**) Force-indentation curves on different polymeric substrates in water with Hertzian curve fits. (**l**) Elastic moduli of PDMS 20:1 and polyacrylamide with different bis-acrylamide concentrations in water.

A major advantage of hydrogel materials is that molecular characteristics can be easily customized by adjusting factors such as monomer size^35^, polymer weight fraction^36^ and curing condition^37^. Depending on the PEG-DA MW, the mechanical properties of the cantilever are tunable. With increasing MW, the cantilevers become more flexible, as indicated by trends in the relative deflection (Fig. 3a). Likewise, with increasing ultraviolet light dose in the curing step, the elastic modulus of PEG-DA MW 250 cantilevers in air increases^38^ (Fig. 3b). To characterize the mechanical properties of hydrogel cantilevers in water, stylus forces were calibrated and transient polymer swelling was first assessed as a function of PEG-DA MW (Supplementary Figs 10 and 11). Under steady-state conditions, hydrated PEG-DA hydrogels with higher average MW monomers exhibited greater swelling (Fig. 3c and Supplementary Fig. 12). Accordingly, the elastic modulus of PEG-DA MW 575 and 700 cantilevers increased in water due to greater stress among the swollen, cross-linked polymer chains^39^,^40^ (Fig. 3d). On the other hand, the elastic modulus of hydrated PEG-DA MW 250 cantilevers decreased by 30%. In this latter case, the lack of swelling is advantageous for AFM applications. Parylene coating is an alternative and optional step to partial metal coating to prevent issues resulting from water absorption (Supplementary Fig. 13). To finely adjust the elastic modulus across a wide range (∼30 MPa to 1.5 GPa), cantilevers were also prepared from PEG-DA MW 250 and 575 mixtures (Fig. 3e and Supplementary Fig. 14). Widely tunable and comparably low elastic moduli of hydrogel cantilevers are in stark contrast with those of silicon-based cantilevers which exhibit a more limited tuning range that typically relies on crystallinity^41^.

Next, we investigated the resonance spectra of a PEG-DA MW 250 cantilever in air and water. The fundamental resonance frequencies in air and water were 53 and 27 kHz, respectively. The quality factors in air and water were 30 and 17, respectively (Fig. 3f). By adjusting the geometrical dimensions of the PEG-DA MW 250 cantilever, we varied the resonance frequency between 4 and 105 kHz in air and between 2 and 51 kHz in water, respectively (Fig. 3g). By adjusting the cantilever dimensions and PEG-DA MW, the spring constant was also varied between 0.000027 and 1,022 N m^−1^ in air, and between 0.000053 and 280 N m^−1^ in water (Fig. 3h and Supplementary Table 1). In particular, the spring constants of some hydrogel cantilevers in water were specifically tuned to match those of commercial silicon cantilevers (ranging from 0.09 to 2 N m^−1^) to compare contact mode AFM imaging performance, as described below. The mechanical force-sensing capabilities of the hydrogel cantilevers were determined by force-displacement measurements^42^ (Supplementary Method, Supplementary Figs 15–17, and Supplementary Table 2). As shown in Fig. 3i–l, the hydrogel cantilever is well-suited for measuring the elastic moduli of various hard and soft polymeric substrates in cases where the hydrogel tip deformation is negligibly small (see Supplementary Discussion, Supplementary Fig. 18 and Supplementary Table 3). The measured elastic moduli of all substrates were in good agreement with results obtained by stylus measurements or reference values (Supplementary Table 4). Collectively, the experiments indicate that the bottom-up fabrication approach permits excellent control over the mechanical properties of hydrogel cantilevers and the spring constant can be readily tuned over at least eight orders of magnitude from ∼0.00003 to ∼1,000 N m^−1^ which is a wider tuning range than that offered by commercial silicon-based cantilevers (Supplementary Fig. 19). It is therefore feasible to employ integrated hydrogel probes in AFM applications on both soft and hard substrates as well as for nanomechanical sensing applications^43^,^44^,^45^,^46^.

### Deflection and resonance stabilities

**Figure 4:**
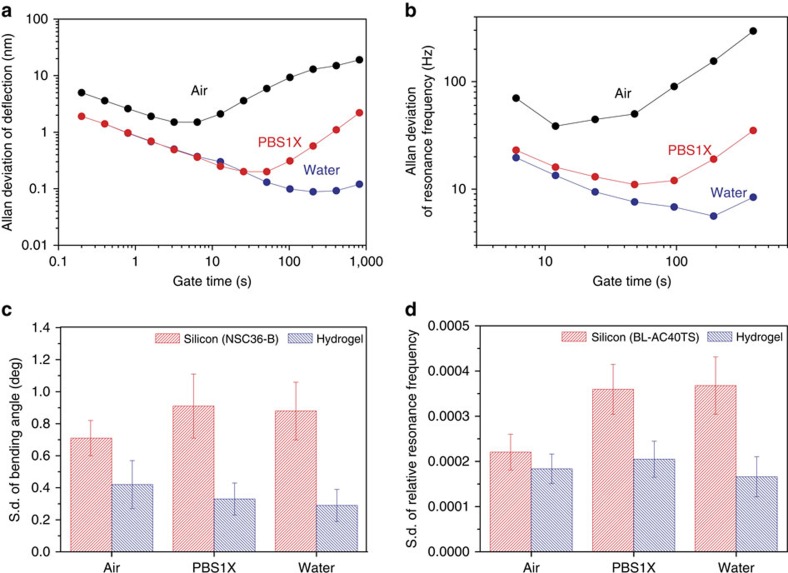
Stability of hydrogel probes in different environmental conditions. (**a**,**b**) Allan deviation of the static deflection of a hydrogel probe (length, width and thickness: 240, 50 and 10 μm) (**a**) and Allan deviation of the resonance frequency of another hydrogel probe (length, width and thickness: 220, 50 and 20 μm) (**b**) in air, water and PBS solution. (**c**,**d**) S.d. of the bending angles of three silicon (NSC36-B, Mikromasch) and three hydrogel probes with similar spring constants (**c**) and s.d. of the relative resonance frequencies of three silicon (BL-AC40TS, Olympus) and three hydrogel probes with similar resonance frequencies in air, water and PBS solution. Error bars represent s.d. of the average from three measurements for each device (*N*=9 measurements for each probe material).

To investigate the stability of hydrogel contact and noncontact probes, we measured time-dependent variations of the static deflection and resonance frequency of hydrogel probes in different surrounding media conditions (Supplementary Discussion and Supplementary Fig. 20a,b). Measurement results were plotted as Allan deviations (Fig. 4a,b) and s.d.'s (Fig. 4c,d). Both the Allan deviations and the s.d.'s of the static deflection and resonance frequency indicate that hydrogel probes are most stable in water. High ionic strength conditions in phosphate-buffered saline (PBS) solution caused the hydrogel probes to be marginally less stable than devices operating in water^47^. Resonance frequencies measured in PBS also differed from measurements conducted in water (Supplementary Fig. 20b). For benchmarking, the stability characteristics of hydrogel probes were further compared with those of commercial silicon probes which had similar spring constants or resonance frequency values (Supplementary Discussion and Supplementary Fig. 20c,d). In air, the hydrogel probes are slightly less stable than the silicon probes for static operation when the fluctuation of static deflection is directly compared (Supplementary Fig. 20c). Once the different probe lengths are taken into account, the hydrogel probes appear to be more stable than the silicon probes (Fig. 4c). On the other hand, in water and PBS conditions, hydrogel probes are more stable than silicon probes for both static and dynamic operation in terms of both absolute and relative figures (Fig. 4c,d and Supplementary Fig. 20c,d). Given these experimental findings that demonstrate hydrogel probes have stable deflection and resonance stabilities in air and liquid conditions, we next explored the potential of hydrogel probes for AFM applications.

### AFM performance of hydrogel probes

**Figure 5:**
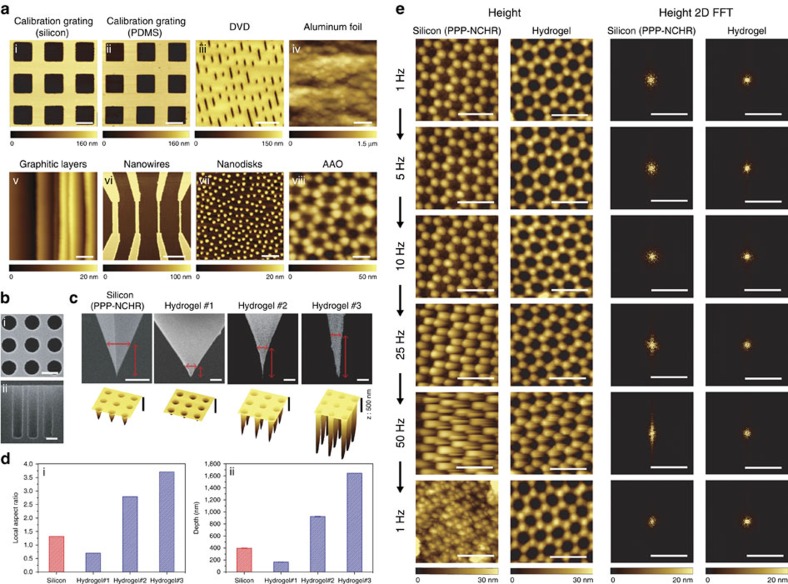
Noncontact mode imaging performance of hydrogel probes and comparison with silicon probes. (**a**) Noncontact mode height images of calibration gratings (silicon and PDMS replica), digital versatile disk media with written data bits, nanowires, aluminium foil, graphitic layers, nanodisks and an AAO sample (70 nm pore diameter). Scale bars are 5, 5, 5, 10, 1, 5, 0.4 and 0.1 μm for (i–viii), respectively. (**b**) Top (i) and side (ii) view of SEM images of cylindrical pores with 1 μm diameter, 1.4 μm pitch and 5 μm depth. The side view was taken after the sample was cleaved. Scale bars are 1 μm. (**c**) Scanning electron microscopy (SEM) images showing tip apex regions of commercial silicon (PPP-NCHR, Nanosensors) and fabricated hydrogel (length, width and thickness: 200, 50 and 20 μm) probes and corresponding 3D representations of noncontact height images for the deep cylindrical pores. All scale bars are 1 μm. (**d**) Local aspect ratio (i) and maximum depth (ii) measured with silicon and hydrogel probes. (**e**) Height images for another AAO sample (35 nm pore diameter) taken by commercial silicon (PPP-NCHR, Nanosensors) and fabricated hydrogel (length, width and thickness: 170, 50 and 20 μm) probes at 1, 5, 10, 25, 50 and 1 Hz, respectively. Height images are 2D fast Fourier transformed (2D FFT) to quantitatively compare image distortions and artifacts. Scale bars for height and 2D FFT images are 100 nm and 200 μm^−1^, respectively. The same colour bars are used for height and 2D FFT images, respectively.

To initially check the feasibility of hydrogel probes for AFM applications, calibration gratings were imaged in contact and noncontact modes with fabricated hydrogel and commercial silicon probes (Fig. 5a, i–ii and Supplementary Fig. 21). Hydrogel probes were then employed to image various samples including digital versatile disk media with data written, aluminium foil, graphitic layers, nanowires, nanodisks and anodized aluminium oxide (AAO) samples (Fig. 5a, iii–viii). Hydrogel probes successfully resolved ∼1–2 nm steps in graphitic layers and 70 nm diameter pores in AAO. Once a softer hydrogel nano-probe (Supplementary Fig. 22) was employed in contact mode imaging of graphitic layers, enhanced friction contrast was obtained (Supplementary Fig. 23). In addition, a silicon sample with a deep cylindrical pore array was prepared by deep reactive ion etching and imaged by three fabricated hydrogel probes with local aspect ratio of 0.7, 2.79 and 3.71 (Fig. 5b). A commercial silicon probe with local aspect ratio of 1.32 was tested as well (Fig. 5c). Here, the local aspect ratio is defined as the ratio of tip height to tip width corresponding to the pore diameter represented with vertical and horizontal arrows in the top panel of Fig. 5c. Three-dimensional representations of noncontact mode height images indicate that hydrogel probes with a deformed pyramidal tip outperform the silicon probe while the hydrogel probe with a regular non-deformed pyramidal tip underperforms the silicon probe (Fig. 5c). The local aspect ratio of each probe and the corresponding maximum depth measured are shown in Fig. 5d. The depth measured by the hydrogel probe with local aspect ratio of 3.71 is ∼4.1 times deeper than that measured by the silicon probe. Once the tip mould is initially replicated from a specific tip shape and then bi-axially compressed, the local aspect ratio always increases. Therefore, hydrogel probes show excellent demonstrated potential to outperform silicon probes in the case of imaging deep pores and trenches.

In addition, the advantage of a relatively low quality factor for high-speed imaging with hydrogel probes was investigated. A hydrogel probe with resonance frequency of 141 kHz and quality factor of 35.1 was used to image an AAO sample with 35 nm diameter pores at various scan rates. For benchmarking, a commercial silicon probe with resonance frequency of 322 kHz and quality factor of 589 was employed (Fig. 5e). Both hydrogel and silicon probes exhibited tip sharpness of ∼10 nm and feedback control parameters for hydrogel and silicon probes were individually optimized at a 1-Hz scan rate, and then maintained for other subsequently higher scan rates. The hydrogel probe maintained similar imaging quality at all scan rates while the silicon probe produced degraded image qualities at scan rates higher than 25 Hz. Height images obtained by the silicon probes exhibited much greater distortions and artifacts at scan rates above 25 Hz. To quantitatively compare the distortions, all height images were two-dimensional fast Fourier transformed (FFT) using the Hanning window to derive the modulus—the absolute value of the complex Fourier coefficient. There were hexagonal bright spots at the centre in common for both probes at all scan rates. While the symmetry of the bright spots was maintained for the hydrogel probe, it was broken and significantly stretched along the slow scan axis for the silicon probe. The superior performance of the hydrogel probes can be explained by the cantilever bandwidth, *B*, which is proportional to the ratio of the resonance frequency, *f*_0_, to the quality factor, *Q*. The *f*_0_/*Q* ratios are 4.02 and 0.55 kHz for hydrogel and silicon probes, respectively. The lower noise observed with the hydrogel probe may be attributed to the higher *f*_0_/*Q* of the hydrogel probe by an order of magnitude because a higher *f*_0_/*Q* is known to be beneficial for high-speed imaging^48^. After imaging at 50 Hz, the AAO sample was imaged again at 1 Hz with both probes to check for any damage. Interestingly, only the hydrogel probe offered image quality similar to that of the first 1-Hz scan. The silicon probe tip was significantly damaged while the hydrogel probe only underwent regular wear (Supplementary Fig. 24). While the imaging performance of the silicon probe could possibly be improved by using one with a softer cantilever^49^, the result nevertheless demonstrates that the hydrogel AFM probe is useful for high-speed imaging applications.

**Figure 6:**
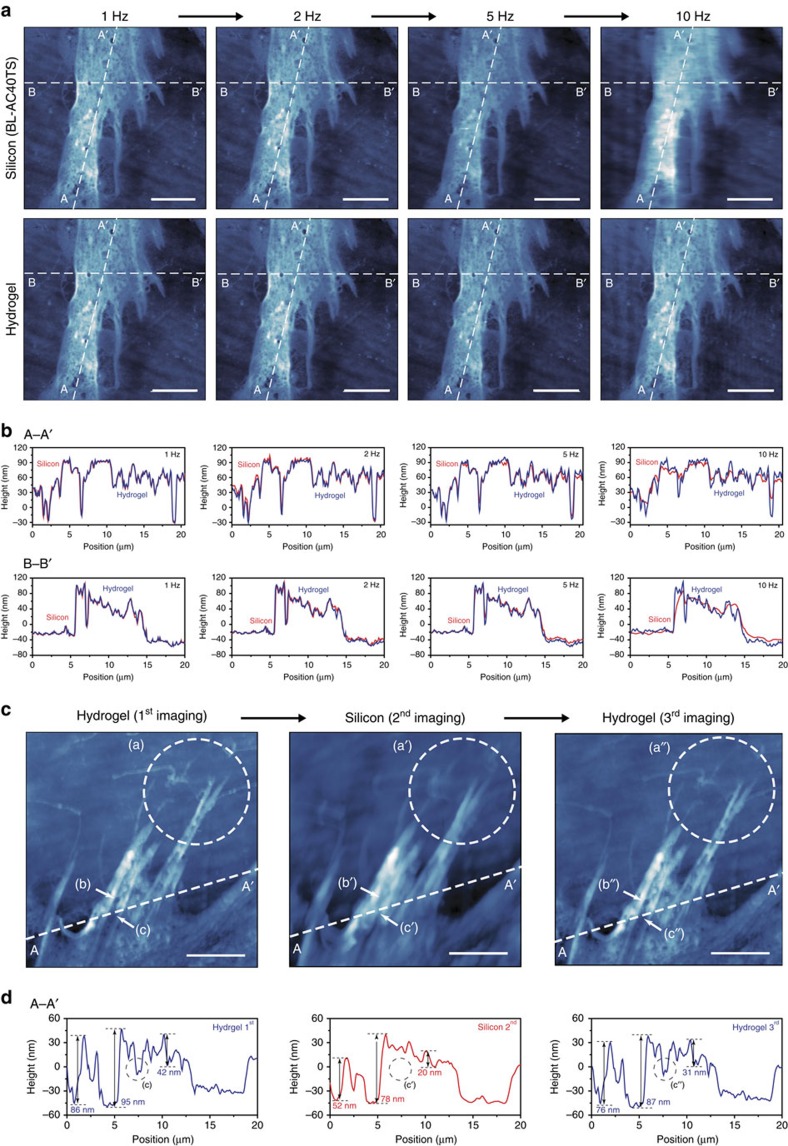
Contact mode imaging results in liquid using hydrogel probes and performance comparison with silicon probes. (**a**) Contact mode height images, with fixed 3 nN contact forces, for human fibroblast cells (MRC-5) in PBS imaged by a commercial silicon probe (first row) and a fabricated hydrogel probe (second row) at various scan rates. All scale bars are 5 μm. (**b**) Height profiles along the A–A′ and B–B′ lines in **a**. (**c**) Contact mode height images, with fixed 300 nN contact force, for MRC-5 cells in PBS sequentially imaged by using hydrogel (first), silicon (second) and hydrogel (third) probes at a scan rate of 1 Hz. All scale bars are 5 μm. (**d**) Height profiles along the A–A′ line in **c**.

We next investigated the imaging performance of hydrogel probes in liquid conditions. First, changes in tip radius induced by physical contact and swelling were shown to be negligible for typical liquid imaging conditions (Supplementary Fig. 25). Experiments with human MRC-5 fibroblast cells and HeLa cells attested to the biological imaging capabilities of the hydrogel AFM probe (Fig. 6 and Supplementary Figs 26–28). The imaging performance of hydrogel and silicon AFM probes on MRC-5 fibroblast cell surfaces was directly compared in parallel experiments. For these experiments, the silicon probe (BL-AC40TS, Olympus) had a spring constant of 2 N m^−1^ and a pyramidal tip with a 10-nm radius of curvature, while the hydrogel probe had a cantilever with a 1-N m^−1^ spring constant and a pyramidal tip with a sub 30 nm radius of curvature. Feedback control parameters were individually optimized for each cantilever used. Comprehensive experiments were performed at increasing scan rates (1–10 Hz) at an identical contact force of 3 nN, and demonstrated that hydrogel and silicon probes had similar performances up to 5 Hz. However, at a 10-Hz rate, only the hydrogel probe maintained consistent image quality while significant distortions are observed in the image obtained by the silicon probe specifically designed for high-speed imaging in liquids (Fig. 6a,b). High-speed imaging for MRC-5 fibroblast cells in noncontact mode performed with both silicon and hydrogel probes also showed the outperformance of hydrogel probes over silicon probes (Supplementary Fig. 29).

Another set of cellular imaging experiments was also conducted to compare image quality^21^ by hydrogel and silicon probes at a high contact force. We prepared a MRC-5 fibroblast cell sample and imaged the same region of the cellular plasma membrane sequentially with a hydrogel, silicon and again hydrogel probe (Fig. 6c,d). Fine details of the cell surface topography were revealed by the hydrogel probe in both experiments, with appreciably lower spatial resolution afforded by the silicon probe even though the same contact force of 300 nN was applied to both probes. It should be stressed that the low spatial resolution obtained with the silicon probe was not due to sample damage by the hydrogel probe because the second scan with the hydrogel probe showed comparable results to the first scan with the hydrogel probe. Next, the cells were imaged in PBS solution under dynamic temperature cycling between 34.5 and 40.4 °C and the results were comparable to those obtained on the same cell surface in measurements conducted at a fixed temperature of around 36 °C (Supplementary Fig. 30). Lastly, human fibroblast cells were continuously imaged in PBS solution with contact mode for 4 h to investigate the long-term stability of hydrogel probes in a practical setting (Supplementary Fig. 31). The image quality was maintained without any evidence of degradation or contamination. The hydrogel-metal interface was durable enough to function properly after 50 h immersion in water (Supplementary Fig. 32).

### Durability test and tip recovery

**Figure 7:**
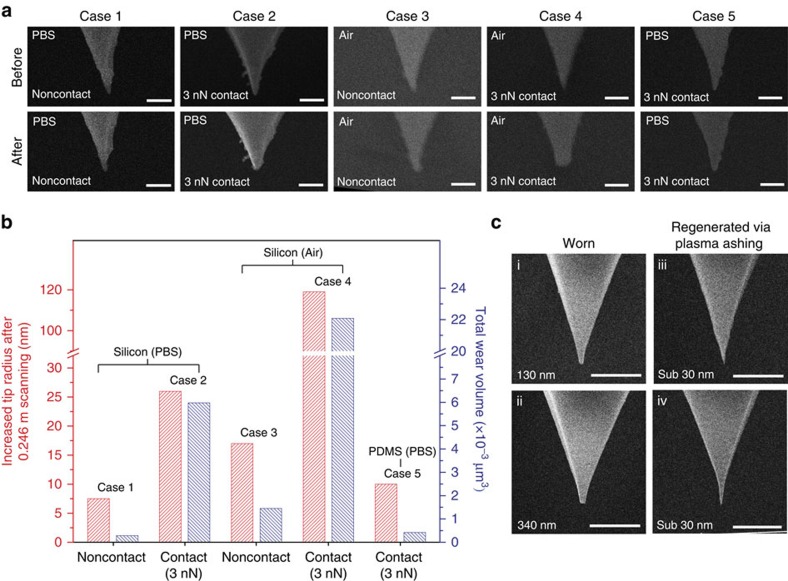
Wear characteristics of hydrogel nano-probes and regeneration via oxygen plasma ashing. (**a**) Scanning electron microscopy (SEM) images of hydrogel probe tips taken before and after wear tests. Imaging mode, condition and surrounding media are indicated on the left side of each micrograph. A hard silicon calibration grating sample was used for test cases 1–4, and a PDMS calibration grating sample replicated from the silicon grating was used for test case 5. All scale bars are 500 nm. (**b**) Increased tip radius and total wear volume after scanning a 0.246-m length (12 frame imagings with a scan size of 40 × 40 μm^2^) for each operating condition. (**c**) SEM images of worn hydrogel nano-probe tips before (i, ii) and after (iii, iv) oxygen plasma ashing. Hydrogel nano-probes with tip radii of 130 and 340 nm were re-sharpened to have sub 30 nm tip radii after plasma ashing. All scale bars are 5 μm.

Hydrogel probes with tip radii of ∼30 nm were employed to investigate wear characteristics in air and PBS solution by repeated imaging on silicon and PDMS gratings (Supplementary Methods and Supplementary Figs 33 and 34). Scanning electron microscopy images show the tip regions of five hydrogel probes before and after wear tests (Fig. 7a). When a hydrogel probe was used to image a hard silicon grating, the tip wear was significantly reduced in noncontact mode (Fig. 7b). Tip wear in noncontact mode is attributed to intermittent hammering impact^50^,^51^ while tip wear in contact mode is attributed to constant sliding friction^52^,^53^. In addition, tip wear during imaging in air occurs more quickly than that observed during imaging in PBS solution (Fig. 7b). The liquid surrounding the hydrophilic tip may act as a lubricant and/or coolant to slow down wear. When a hydrogel probe was used to image a soft PDMS grating, tip wear was significantly decelerated compared to imaging on the hard silicon sample (Fig. 7b). Nonetheless, with any operation mode and imaging condition, wear of the hydrogel tip cannot be completely prevented. One can readily replace worn hydrogel probes with new hydrogel probes through fast and cost-effective fabrication means. We applied oxygen plasma ashing^54^ to re-sharpen worn hydrogel probes. Worn hydrogel probes with tip radii of 130 and 340 nm were regenerated to have sub 30 nm tip radii after oxygen plasma ashing (Fig. 7c). This post-treatment step is applicable to re-sharpening of hydrogel probe tips degraded during any AFM application.

### Multifunctional capabilities via materials encapsulation

**Figure 8:**
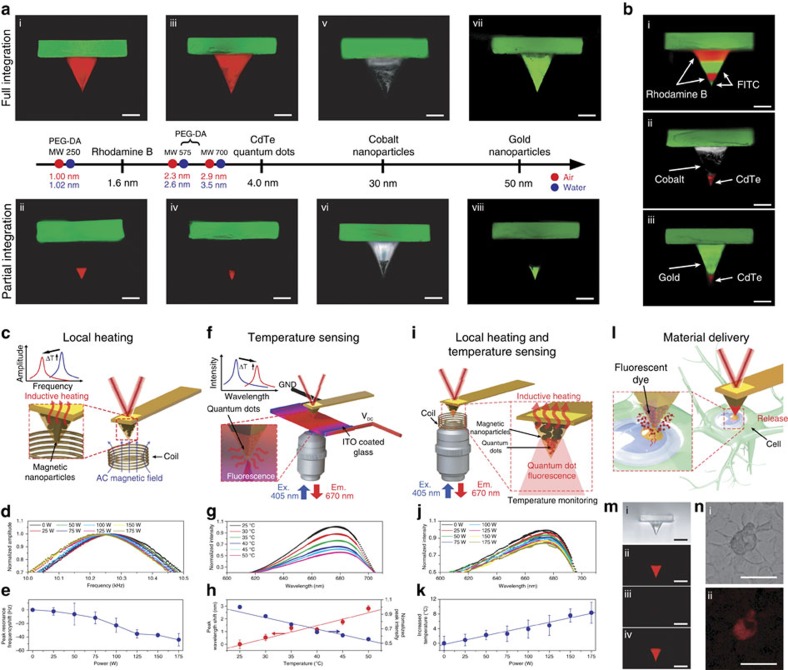
Programming of multifunctional hydrogel nano-probes. (**a**) Hydrogel nano-probes with integrated functional nanomaterials achieved through materials encapsulation. Depending on the encapsulated nanomaterial, the tip structure is composed of PEG-DA with varying MW as specified: (i, ii) Rhodamine B dye (MW 575), (iii, iv) cadmium telluride (CdTe) quantum dots (MW 700), (v, vi) cobalt (Co) nanoparticles (MW 250) and (vii, viii) gold (Au) nanoparticles (MW 700). The mesh sizes of PEG-DA monomers with different MWs are presented. All scale bars are 10 μm. (**b**) Multifunctional hydrogel nano-probes with sequential loading of FITC and Rhodamine B (i), CdTe quantum dots and Co nanoparticles (ii), and CdTe quantum dots and gold nanoparticles (iii). All scale bars are 10 μm. (**c**) Schematic for local inductive heating. (**d**) Normalized amplitude spectra of a hydrogel nano-probe with a Co nanoparticle-embedded tip under induction heating in water. (**e**) Resonance frequency shift as a function of the input power. (**f**) Schematic for local temperature sensing via fluorescence quenching of quantum dots. (**g**) Fluorescence spectra from a hydrogel nano-probe with a CdTe quantum dot-embedded tip in contact with an ITO-coated glass substrate under Joule heating in water. (**h**) Peak wavelength shift and the normalized peak intensity as a function of the temperature at the tip contact point. (**i**) Schematic for a dual function hydrogel nano-probe for local heating and temperature sensing. (**j**) Fluorescence spectra from a hydrogel nano-probe with sequentially embedded CdTe quantum dots and Co nanoparticles under induction heating. (**k**) Temperature increase as a function of the power applied to the induction coil. (**l**) Schematic for localized materials delivery. (**m**) Bright-field and fluorescence microscopy images of a hydrogel nano-probe before (i) and after (ii) the first loading as well as after the first delivery (iii) and after the second loading (iv), respectively. All scale bars are 20 μm. (**n**) Bright-field optical microscopy image (i) and fluorescence microscopy image (ii) for a breast cancer cell (MCF-7) demonstrating localized delivery of Rhodamine B dye. Scale bars are 10 μm. All error bars represent the s.d. of the mean with *N*=3 measurements.

Beyond imaging capabilities, hydrogel AFM probes enable multifunctional features based on materials encapsulation^55^,^56^. Taking advantage of the bottom-up fabrication approach, we incorporated various nanomaterials into the aqueous pre-polymer solution before photopolymerization. By tuning the hydrogel mesh network dimensions^57^, different nanomaterials can be encapsulated indefinitely or exhibit controlled release profiles. Due to the modular design of the hydrogel tip and cantilever, the hydrogel material in both parts can be varied to encapsulate different materials (Supplementary Fig. 35 and Supplementary Table 5). As demonstrated in Fig. 8a, this approach can be applied to fluorescent dyes (Rhodamine B), quantum dots (cadmium telluride, CdTe), magnetic nanoparticles (cobalt, Co) and plasmonic nanoparticles (gold, Au) across a wide range of sizes from 1.6 to 50 nm diameter. The approach enables nanomaterials to come into close proximity to target interfaces (for example, cell surfaces) in a highly controlled manner, and the hydrogel network minimizes nanomaterial toxicity^58^ and related issues. With different loading methods, the nanomaterials were encapsulated throughout the entire tip (Fig. 8a, i, iii, v and vii) or into the tip apex only (Fig. 8a, ii, iv, vi and viii). This control was achieved by filling the mould completely with pre-polymer solution loaded with nanomaterial, or only partial filling with the former and then filling the remaining volume in the tip mould with pre-polymer solution without loaded nanomaterial. Following the tip attachment process as described above, the tips were integrated with the cantilevers to form the functional probes. Using this approach, several nanomaterials could also be sequentially encapsulated within the AFM tip, demonstrating excellent spatial control (Fig. 8b).

To explore the multifunctional capabilities of hydrogel probes, we focused on materials encapsulation as well as several AFM applications: local heating via induction, temperature sensing via quantum dot fluorescence quenching and swelling-mediated materials delivery. In all the experiments described below, the tips were composed of PEG-DA of varying MW and had a pyramidal tip shape. With embedded magnetic Co nanoparticles in PEG-DA MW 250, out-of-plane bending was induced by a permanent magnet (Supplementary Fig. 36) and localized inductive heating was induced by an externally applied alternating current magnetic field^59^ (Fig. 8c and Supplementary Fig. 37). The temperature rise was indirectly confirmed by a decrease in the fundamental resonance frequency of the cantilever in water (Fig. 8d,e) and could also be detected in air (Supplementary Fig. 38). To confirm that the temperature rise is due to the encapsulated Co nanoparticles, the same experiment was also performed using an identical hydrogel probe without embedded Co nanoparticles and there was a negligible signal response. With embedded CdTe quantum dots in PEG-DA MW 700, local temperature sensing^60^ was enabled when the tip contacted an indium tin oxide-coated glass substrate under Joule heating (Fig. 8f). Fluorescence emission intensities decreased and the peak wavelength increased as the temperature at the contact point increased in water (Fig. 8g,h) or air (Supplementary Fig. 39) where embedded CdTe quantum dots remained stable due to their diameters larger than mesh sizes of PEG-DA monomers (Supplementary Fig. 40). Supplementary Figure 41 summarizes temperature sensitivities of CdTe quantum dots embedded hydrogel nano-probes. Importantly, local induction heating and *in situ* temperature sensing could be performed simultaneously in water or air using a multifunctional tip loaded with both Co and CdTe nanoparticles (Fig. 8j,k and Supplementary Figs 42 and 43).

The encapsulation and controlled release of Rhodamine B dye from a PEG-DA MW 575 tip was also investigated (Fig. 8l). When the hydrogel tip is in ambient conditions, the encapsulated dye remains stable but diffuses away over time in aqueous conditions. The rate of release depends on properties of the encapsulated molecule along with the polymer mesh size, and is controllable. Dye encapsulation can take place during fabrication through inclusion in the pre-polymer solution, or after fabrication if the tip is immersed in a molecular ink depot^39^ (Supplementary Fig. 44a). A single hydrogel probe can be used for multiple rounds of materials loading and delivery (Fig. 8m). Vice versa, a dye-loaded tip can make contact with a polymeric substrate and, with sufficient contact force and time, locally deliver the dye molecules. Localized materials delivery was optimally achieved with high dye concentration and short contact time (Supplementary Fig. 44b). The experimental results are consistent with simulations of the delivered Rhodamine B concentration as a function of the contact time and molecular diffusion properties in the system (Supplementary Figs 45 and 46). Extending this concept to cellular applications, localized delivery was demonstrated on the surface of live human breast cancer cells at 37 °C (Fig. 8n). A gentle contact force of 1–2 nN was applied in conjunction with a relatively blunt tip of 150 nm curvature radius to permit effective materials delivery without tip penetration into the cell membrane. This demonstration validates the potential of localized materials delivery with hydrogel tips which offer size-controlled delivery of loaded materials based on a tunable mesh size through the direct contact area. Such aspects distinguish the proposed swelling-mediated materials delivery from dip-pen nanolithography^61^. Altogether, the application examples highlight the range of possibilities for programmable and multifunctional design of hydrogel AFM probes. Looking forward, it should also be stressed that bulk fabrication of hydrogel probes at the wafer scale is possible. Towards this goal, preliminary data supports that hydrogel nano-probes obtained by our PDMS-based fabrication scheme are free of silicone oil contaminants^62^ (Supplementary Fig. 47), can be produced in parallel by a slit type shadow mask (Supplementary Fig. 48), and the corresponding production costs are forecast to be appreciably lower than the production costs for silicon probes (Supplementary Fig. 49).

## Discussion

Herein, we have demonstrated the bottom-up fabrication of hydrogel AFM probes to enable a wide range of stiffness tuning and nanoscale tip geometries. Importantly, by encapsulating functional nanomaterials into the hydrogel tip, we also demonstrated the programmable and multifunctional capabilities of hydrogel probes, including local heating, temperature sensing and materials delivery among other possibilities. The developments in this study set new precedents for AFM probe design, and a number of innovations following this idea can be envisioned. In particular, hydrogel cantilevers may facilitate high-speed imaging capabilities due in part to lower elastic moduli and quality factors which make the imaging tip compliant and effectively absorb mechanical noise during scanning. Given the breadth of hydrogel research and functionalization possibilities, there is also strong motivation to develop the hydrogel probe as a platform for various applications and materials encapsulation strategies. A key advantage is the simplicity of bottom-up fabrication with straightforward photopolymerization reactions. The fabrication steps in our scheme are widely used in polymer chemistry and materials science, accessible to many researchers and generalizable to many different polymer choices. Considering that silicon probe fabrication strategies are highly specialized and offer limited customization, the opportunities afforded by hydrogel AFM probes are compelling to further explore. Without a doubt, silicon probes have played a critical role in AFM instrumentation and will continue to do so. Our contention is not that hydrogel probes will replace silicon probes. Both types of probes have important roles and complement one another. Rather, hydrogel probes are a harbinger of the potential that lies ahead with the convergence of soft matter and nanotechnology. Such potential can also be readily extended to fabricating hydrogel cantilevers for nanomechanical sensor devices, including in parallel array configurations for high-throughput applications.

## Methods

### Hydrogel preparation

Aqueous pre-polymer solutions were prepared using PEG-DA monomers (Sigma Aldrich), with phenylbis(2,4,6-trimethylbenzoyl) phosphine oxide (Sigma Aldrich) as the photoinitiator. The PEG-DA:photoinitiator weight ratio was 98:2. In most experiments, only one type of PEG-DA monomer was used with the average MW varied between 250 and 700 g mol^−1^. In some experiments, PEG-DA mixtures of the MW 250 and MW 575 monomers were prepared to finely tune the average MW. After preparation, the aqueous pre-polymer solutions were magnetically stirred for 24 h and kept in brown bottles wrapped in aluminium foil to prevent light exposure before experiment.

### Hydrogel cantilever fabrication

Replica moudling was employed to fabricate hydrogel cantilevers (Supplementary Fig. 1). First, an SU8 cantilever master mould was fabricated. SU8-2015 and SU8-2005 photoresists (Microchem) were spin-coated onto 4-inch silicon wafers for 30 s (5,000 and 3,000 rpm for 10 and 15 μm target thicknesses, respectively, with 2015 type; and 3,000 rpm for 5 μm target thickness with 2005 type), and then soft-baked for 4 min at 105 °C. The photoresists were next exposed to a 365-nm i-line ultraviolet source (20 mW cm^−2^; MA6, Karl Suss) for 15 and 10 s for 2015 and 2005 types, respectively. A commercial silicon cantilever handle (PPP-NCHR, Nanosensors) was used to define the cantilever handle mould. A PDMS pre-polymer mixture (weight ratio 10:1; Sylgard 184, Dow Corning) was poured onto the two master moulds and cured on a hot plate at 100 °C for 60 min. The replica from the top cantilever mould was cut to have an inlet for injecting PEG-DA pre-polymer solution and aligned on top of the other replica from the bottom handle mould. The aqueous pre-polymer solution (98:2 weight ratio of PEG-DA and photoinitiator) was pipetted near the inlet and introduced into the aligned replica mould assembly via capillary action and cured by using a ultraviolet light-emitting diode source (CBT-90-ultraviolet-C31-M400-22, Luminus Devices) for 5 s (ultraviolet dose: 590 mJ cm^−2^). After the top cantilever replica mould was removed, the cured PEG-DA cantilever was cut to the desired length^40^ and separated from the bottom handle replica mould, which was facilitated by an oxygen inhibition layer between the PDMS and PEG-DA hydrogel^41^.

### Hydrogel tip integration

A 4-inch single crystal silicon wafer was thermally oxidized to grow a 600-nm-thick silicon dioxide film. The oxide layer was patterned in a reactive ion etcher with a photoresist mask (PR1-1000 A, Futurrex) to leave exposed circular regions (10–20 μm in diameter). This exposed silicon region on the oxide-patterned wafer was etched anisotropically in 30 wt% aqueous potassium hydroxide solution at 70 °C for 15 min to form negative pyramids or etched isotropically in a mixture of hydrofluoric, nitric and acetic acids (HNO_3_:HF:CH_3_COOH=95:2:3 v v^−1^ v^−1^), at room temperature for 5 min resulting in negative hemispheres. Then, the oxide layer was removed in 49% hydrofluoric acid and the wafer was cleaned with piranha solution, a mixture of sulfuric acid and hydrogen peroxide (H_2_SO_4_:H_2_O_2_=3:1 v v^−1^, 10 min, 100 °C) and deionized water. 20 ml of the photoresist (PR1-2000 A, Futurrex) mixed with 1 μl of a polystyrene microsphere suspension (20 μm in diameter, 4.55 × 10^7^ particles per ml, Polysciences Inc.) was spin-coated (1,000 rpm/15 s-3,000 rpm/20 s-1,000 rpm/10 s) onto a 4-inch silicon wafer, and then baked on a hot plate at 120 °C for 1 min to embed the microspheres into a thin layer of photoresist. The wafer with negative pyramids or hemispheres and the wafer with embedded microspheres was silanized under vacuum conditions (−80 kPa) for 30 min by 95% trichlorosilane (tridecafluoro-1,1,2,2-tetrahydrooctyl, Gelest) before PDMS replica moudling. PDMS pre-polymer was poured onto silanized wafers and cured on a hot plate at 95 °C for 40 min. The cured PDMS substrate was peeled off and cut for preparing embedded sphere moulds (Supplementary Fig. 2a). The cured PDMS substrate with positive hemispheres or pyramids was silanized to be used as the master mould. Once the PDMS pre-polymer was poured and cured on the silanized PDMS substrate, the final PDMS substrate was peeled off and cut for preparing hemisphere (H; Supplementary Fig. 2b) and pyramid (P) moulds (Supplementary Fig. 2c). The fabrication process for pyramid moulds is similar to that of hemisphere moulds except for the shape of the etched silicon. The P mould can be deformed by using a custom-made compression jig (Fig. 2a) to control the aspect ratio and radius of curvature of the pyramidal tip. Embedded sphere and H tip moulds are typically used without deformation. Each tip mould placed on top of the ultraviolet light-emitting diode was filled with PEG-DA pre-polymer solution. After a hydrogel cantilever contacted the PEG-DA filled tip mould, ultraviolet exposure was applied to cure the tip. In the case of deformed pyramid moulds, lateral or vertical compression was applied by using piezomotors (Picomotor #8303, Newport) within the strain range of 10–14% and 2–6%, respectively. If the empty mould is compressed first and then filled with hydrogel, the apex of the deformed pyramid mould may not be completely filled. Excess PEG-DA pre-polymer solution on tip mould compression was guided along the cantilever via capillary action over the handle. No special care is necessary when the cured tip integrated on the cantilever is separated from the mould due to the volume contraction of the cured PEG-DA hydrogel and the oxygen inhibition layer.

### Cell preparation

Human breast cancer cells (MCF-7, Sigma), human fibroblast cells (MRC-5, Japanese Collection of Research Bioresources Cell Bank; JCRB Cell Bank) and HeLa cells (ATCC CCL-2, ATCC) were used in experiments. Cells were maintained in Eagle's Minimum Essential Medium (ATCC 30-2003, ATCC) supplemented with 10% foetal bovine serum (SV30160.03IR, Hyclone, Thermo Fisher Scientific) and 1% penicillin/streptomycin (10378016, Invitrogen Life Technique) at 37 °C in a humidified atmosphere containing 5% CO_2_. Cells were harvested for subculture post trypsinization, washed and re-suspended at 1 × 10^5^ ml^−1^ concentration in a normal growth medium. For AFM experiments, the cell concentration was adjusted to ∼1 × 10^3^ ml^−1^, and then 1 ml of the cell suspension was seeded on a 35-mm culture dish. Cells were washed by PBS for 5 min and fixed by 4% paraformaldehyde before AFM experiment.

### Oxygen plasma ashing of worn hydrogel tip

Worn hydrogel nano-probes with various tip radii were oxygen plasma-ashed by reactive ion etching (RIE 80 plus, Oxford Instrument) with a forward power of 43 W, oxygen flow rate of 6 s.c.c.m., and chamber pressure of 30 mTorr. With these processing parameters, an average etch rate of ∼10 nm min^−1^ was obtained. Considering the tip radius of a worn hydrogel nano-probe and the average etch rate, the processing time was determined for each hydrogel nano-probe.

## Additional information

**How to cite this article:** Lee, J. S. *et al*. Multifunctional hydrogel nano-probes for atomic force microscopy. *Nat. Commun.* 7:11566 doi: 10.1038/ncomms11566 (2016).

## Supplementary Material

Supplementary Information Supplementary Figures 1-49, Supplementary Tables 1-5, Supplementary Discussion, Supplementary Methods and Supplementary References
