# Supplementary material for: Multifunctional hydrogel nano-probes for atomic force microscopy
Source: Nat Commun. 2016 May 20;7:11566. doi: 10.1038/ncomms11566 (PMC4876479; doi:10.1038/ncomms11566)
Supplement: Supplementary Information — Supplementary Figures 1-49, Supplementary Tables 1-5, Supplementary Discussion, Supplementary Methods and Supplementary References [file ncomms11566-s1.pdf]

Supplementary Figures

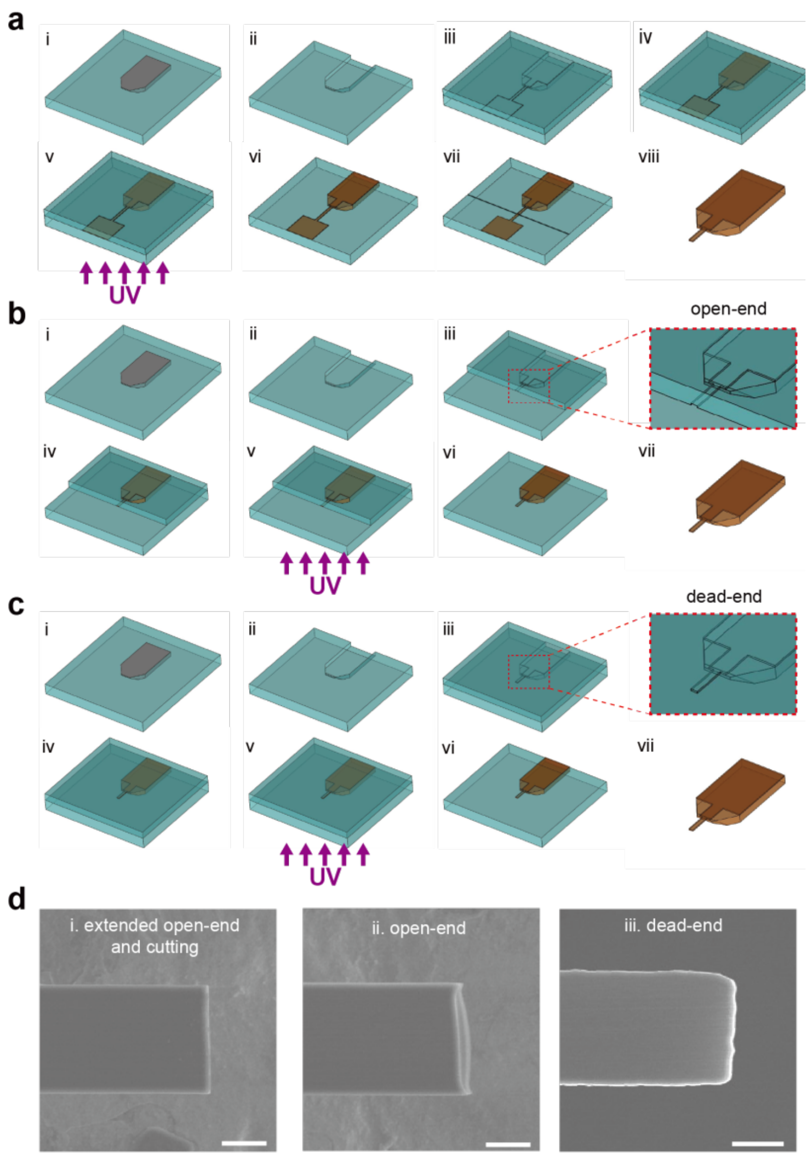

Supplementary Figure 1 | Three fabrication strategies for tipless PEG-DA hydrogel cantilevers. **a,** Fabrication process for tipless hydrogel cantilevers using the extended open-end mold and cutting (i: Mold PDMS over a silicon base, ii: Remove the silicon base and cut, iii: Place the cantilever mold on top of the base platform, iv: Fill pre-polymer solution into the mold assembly, v: Cure hydrogel with UV light, vi: Remove the cantilever mold, vii: Cut the cantilever to the desired length, viii: Remove the fabricated cantilever). **b,** Fabrication process for PEG-DA hydrogel cantilevers using an open-end mold. Fabrication steps are the same as those shown in Supplementary Fig. 1a except that the cantilever mold has a fixed length with an open end. When a pre-polymer solution is introduced into the mold, the open end acts as a stop valve. **c,** Fabrication process for PEG-DA hydrogel cantilevers using a dead-end mold. Fabrication steps are similar to those shown in Supplementary Fig. 1a except that the cantilever mold has a fixed length with the end closed. **d,** Scanning electron micrographs of fabricated hydrogel cantilevers obtained by using the extended open-end mold with cutting afterwards (i), the open-end mold (ii), and the dead-end mold (iii). The open-end and dead-end mold methods result in concave and convex corners, respectively, while the extended open-end mold in conjunction with cutting provides right-angled corners. All scale bars are 25 μm.

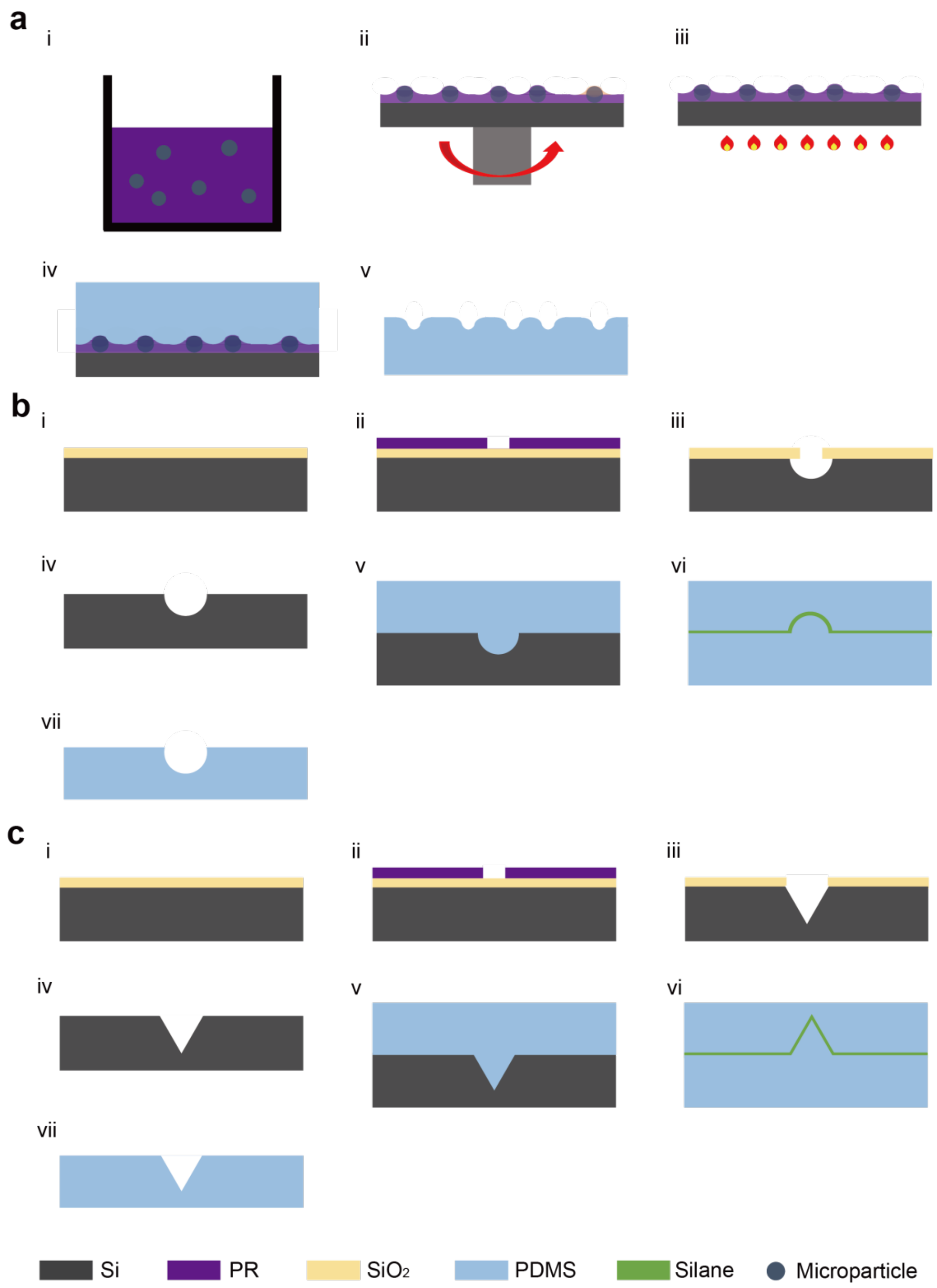

Supplementary Figure 2 | Fabrication of PDMS molds for controlling the shape of hydrogel tips. **a,** Fabrication scheme for the embedded-sphere tip mold (i: Mix photoresist with microparticles, ii: Spin-coat the microparticle-laden photoresist, iii: Soft-bake, iv: Pour and cure PDMS, v: The embedded sphere tip mold fabricated). **b,** Fabrication scheme for the hemispherical tip mold (i: Grow 600 nm thick silicon dioxide layer, ii: Spin-coat and pattern a photoresist mask, iii: Etch silicon dioxide using RIE, remove the photoresist mask, and etch the underlying silicon isotropically using HNA, iv: Remove the silicon dioxide mask using 49% HF solution, v: Pour and cure PDMS to obtain a positive hemispherical mold, vi: Pour and cure PDMS on top of the silanized positive hemispherical mold to obtain a negative hemispherical mold, vii: The hemispherical tip mold fabricated). **c,** Fabrication scheme for the pyramidal tip mold. Processes are the same as those for the hemispherical tip mold except the silicon etching step. 30 wt% KOH solution is used to etch the silicon anisotropically at 70 °C.

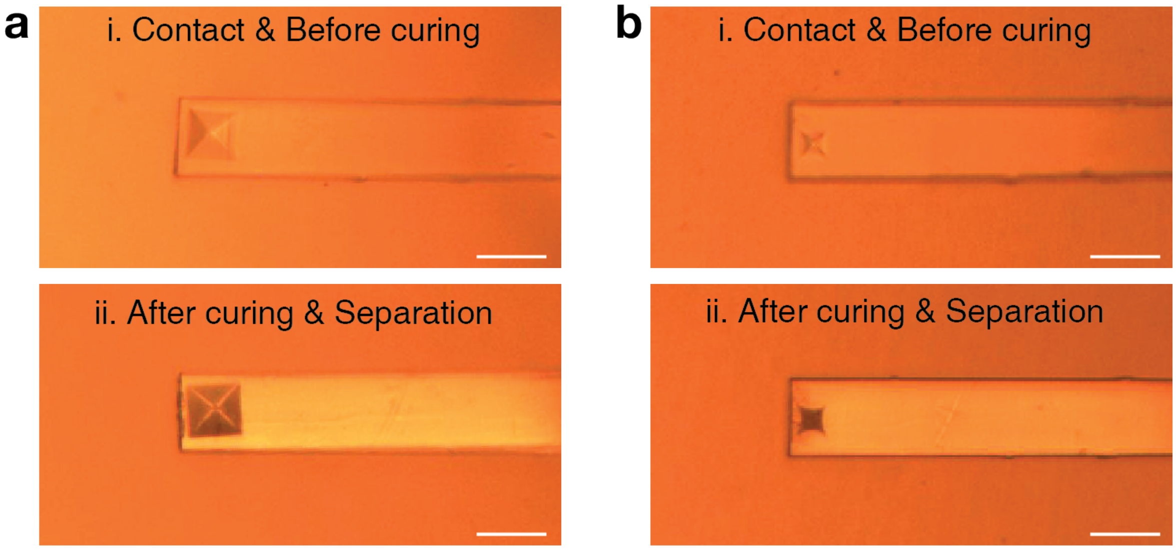

Supplementary Figure 3 | Control of the tip position and compression during the integration of undeformed and deformed pyramid tips. a, Optical micrographs showing a tipless hydrogel cantilever (length, width, and thickness: 400, 50, and 15 µm) in contact with a pre-polymer solution-filled undeformed pyramid mold (i) and tip-integrated hydrogel cantilever (ii). **b,** Optical micrographs showing a tipless hydrogel cantilever in contact with a pre-polymer solution-filled deformed pyramid mold (i) and tip-integrated hydrogel cantilever (ii). All micrographs are taken from the side opposite to the tip integration. All scale bars are 50 µm.

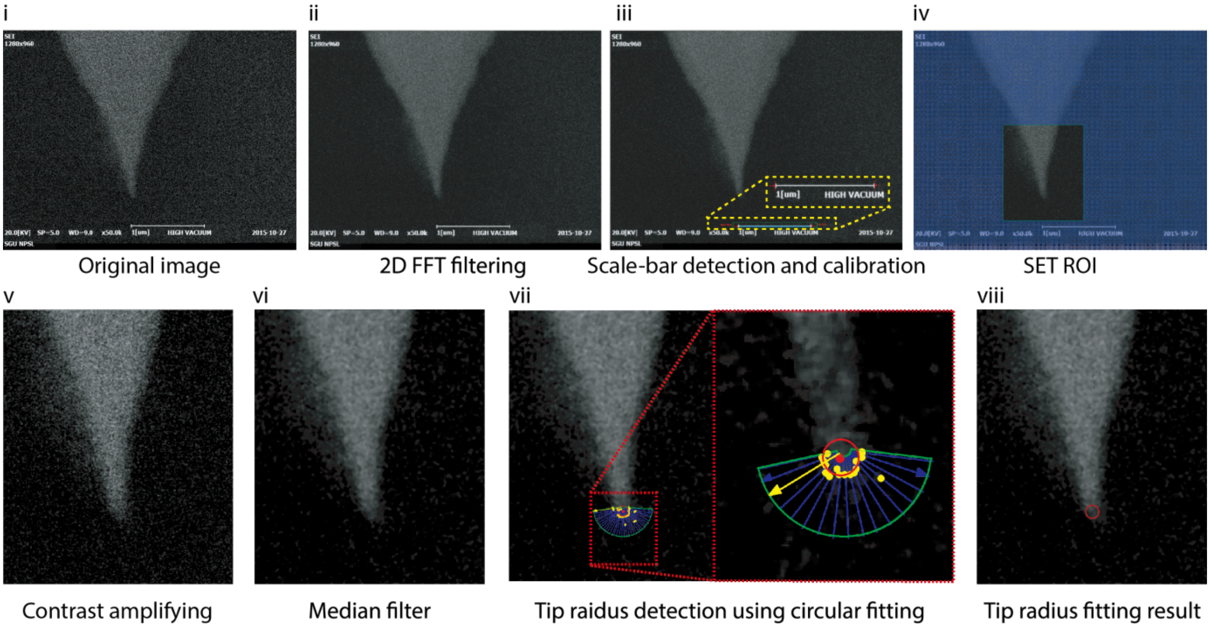

Supplementary Figure 4 | Tip radius estimation protocol. i: Load an SEM image of the hydrogel tip. ii: 2D FFT Low pass filtering, iii: Scale bar detection and pixel-to-length calibration, iv: Define region of interest (ROI), v: Adjust the image contrast to amplify tip-background boundaries, vi: Median filtering for smoothing and reducing noises, vii: Edge detection by brightness thresholding and circular fitting for estimating the tip radius, viii: Tip radius fitting result.

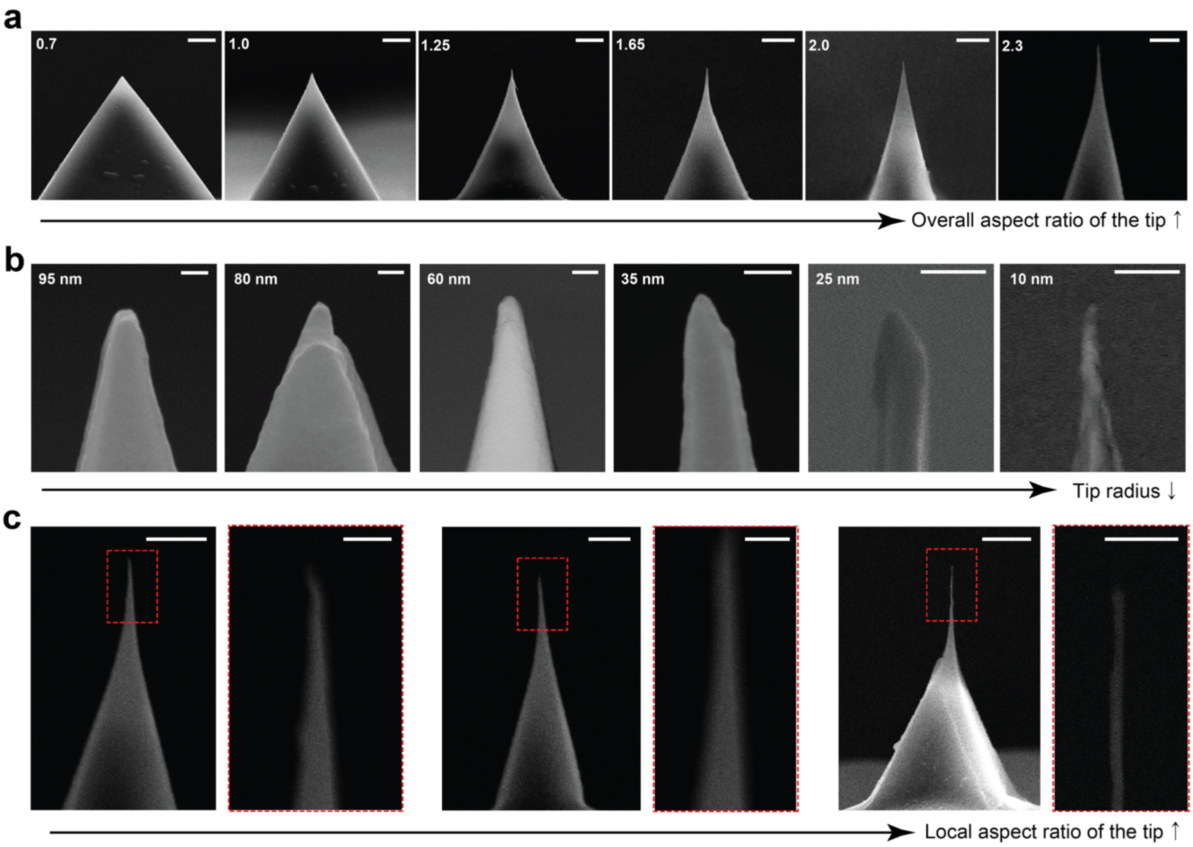

Supplementary Figure 5 | Deformed pyramidal tips with various aspect ratios, radii, and shapes. a, SEM images of hydrogel tips sorted in the order of increasing overall aspect ratio (numbers provided at the upper left corners). All scale bars are 5 μm, **b,** SEM images of hydrogel tips sorted in the order of decreasing tip radius (numbers provided at the upper left corners). All scale bars are 200 nm. **c,** SEM images showing hydrogel tips with high local aspect ratios resulting from large bi-axial compressions. All scale bars are 5 and 1 μm for the left and right of each set, respectively.

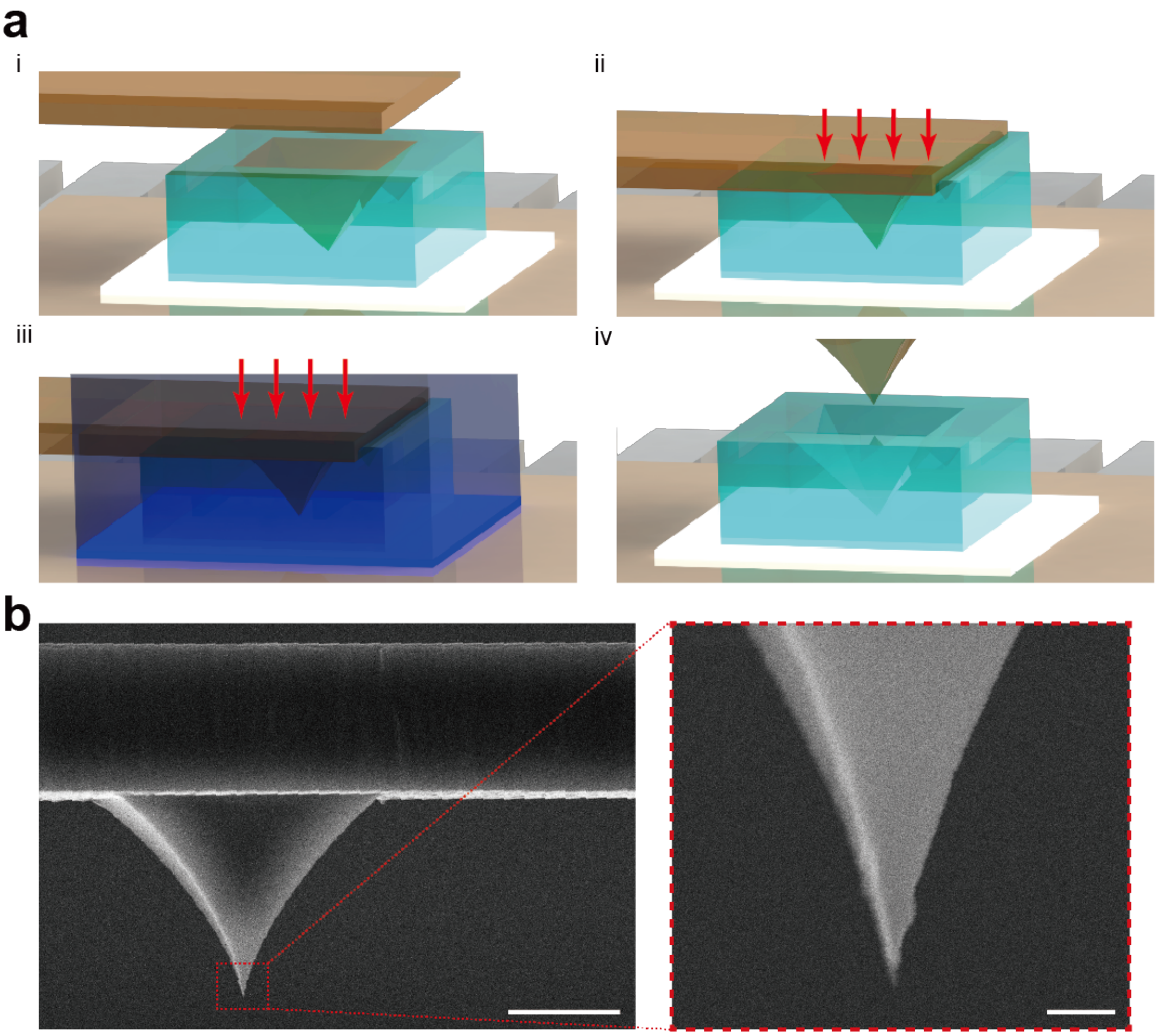

Supplementary Figure 6 | Vertical tip mold compression for tip sharpness improvement. a, Vertical compression scheme (i: Align a tipless hydrogel cantilever to a pre-polymer solution-filled pyramid tip mold, ii: Vertically compress the pyramid tip mold. iii: UV curing, iv: Fabricated hydrogel nano-probe with a deformed pyramid tip via vertical compression). **b,** SEM images of a hydrogel nano-probe with a vertically compressed tip. Scale bars are 10 and 1 μm for the left and right images, respectively.

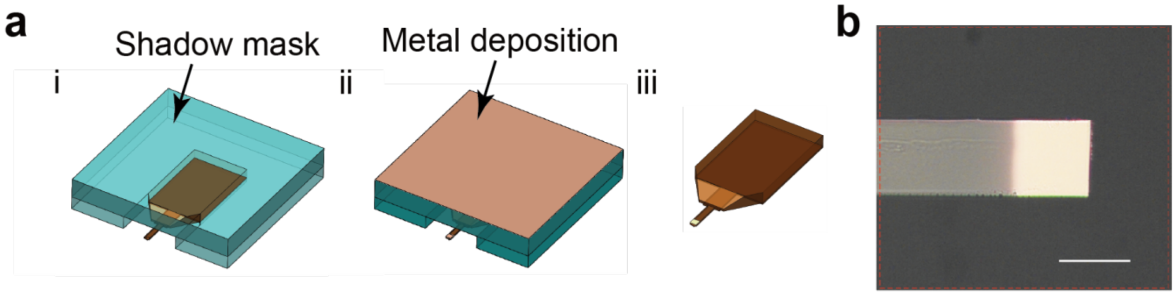

Supplementary Figure 7 | Partial metal coating. a, Schematics for partial metal coating using shadow masks (i: Place cantilevers underneath the shadow mask with the free end partially exposed, ii:Metal deposition, iii: Hydrogel probe partially deposited with metal on its end), **b,** Metal deposited hydrogel probe (10% coverage of the whole cantilever length). Scale bar is 50 μm.

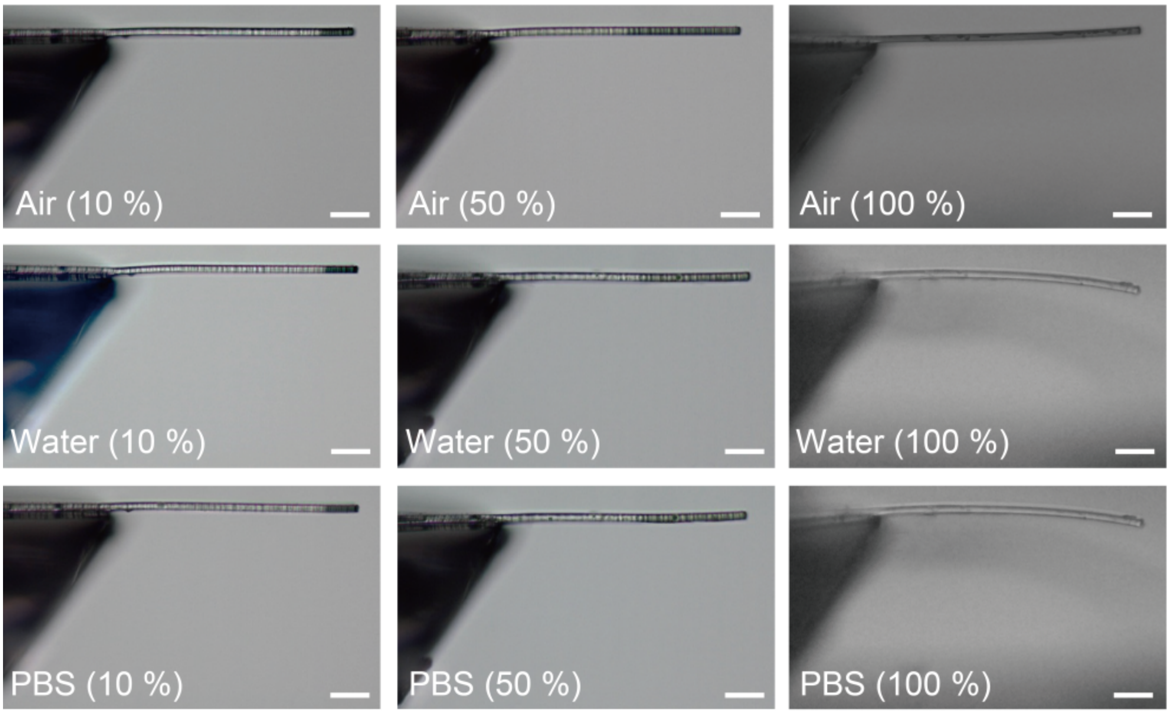

Supplementary Figure 8 | Effect of the coverage of metal coating on swelling-induced bending of PEG-DA hydrogel cantilevers. The top side of the hydrogel cantilever was fully or partially coated with a metal thin film. Side view optical microscope images of PEG-DA hydrogel cantilevers, in which the metal film surface coverage is 10, 50, and 100% from the free end, respectively, in air, water, and phosphate-buffered saline, (PBS) solution respectively. The deposited metal was 120 nm thick. All scale bars are 50 µm.

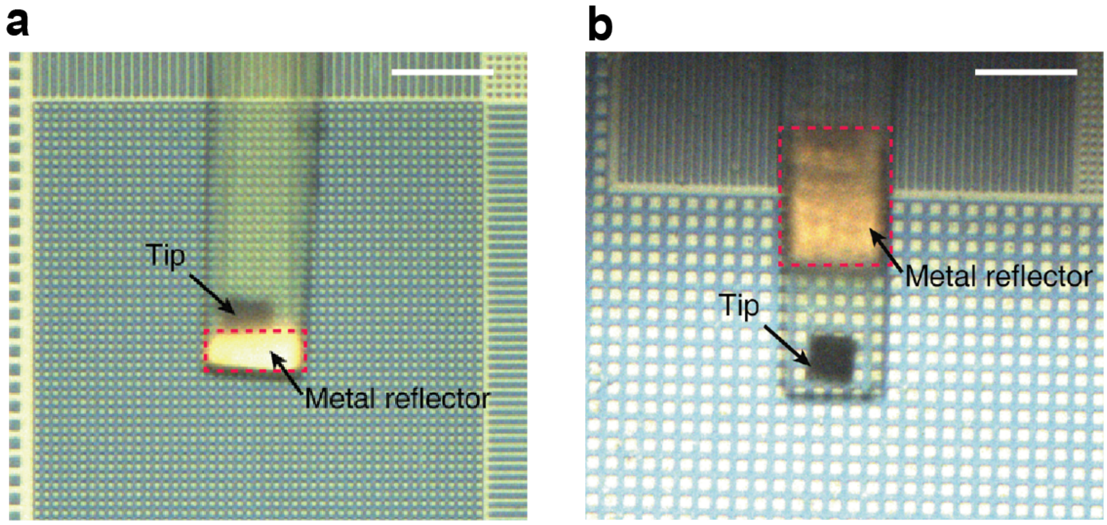

Supplementary Figure 9 | Optical micrographs of hydrogel nano-probes with partial metal coatings hovering over a calibration grating. a, Transparent property of hydrogel and partial metal deposition (at the free end) make the sample visible through the cantilever. **b,** Transparent property of hydrogel and partial metal deposition (offset from the free end) make the sample and tip position visible. Scale bars are 50 µm.

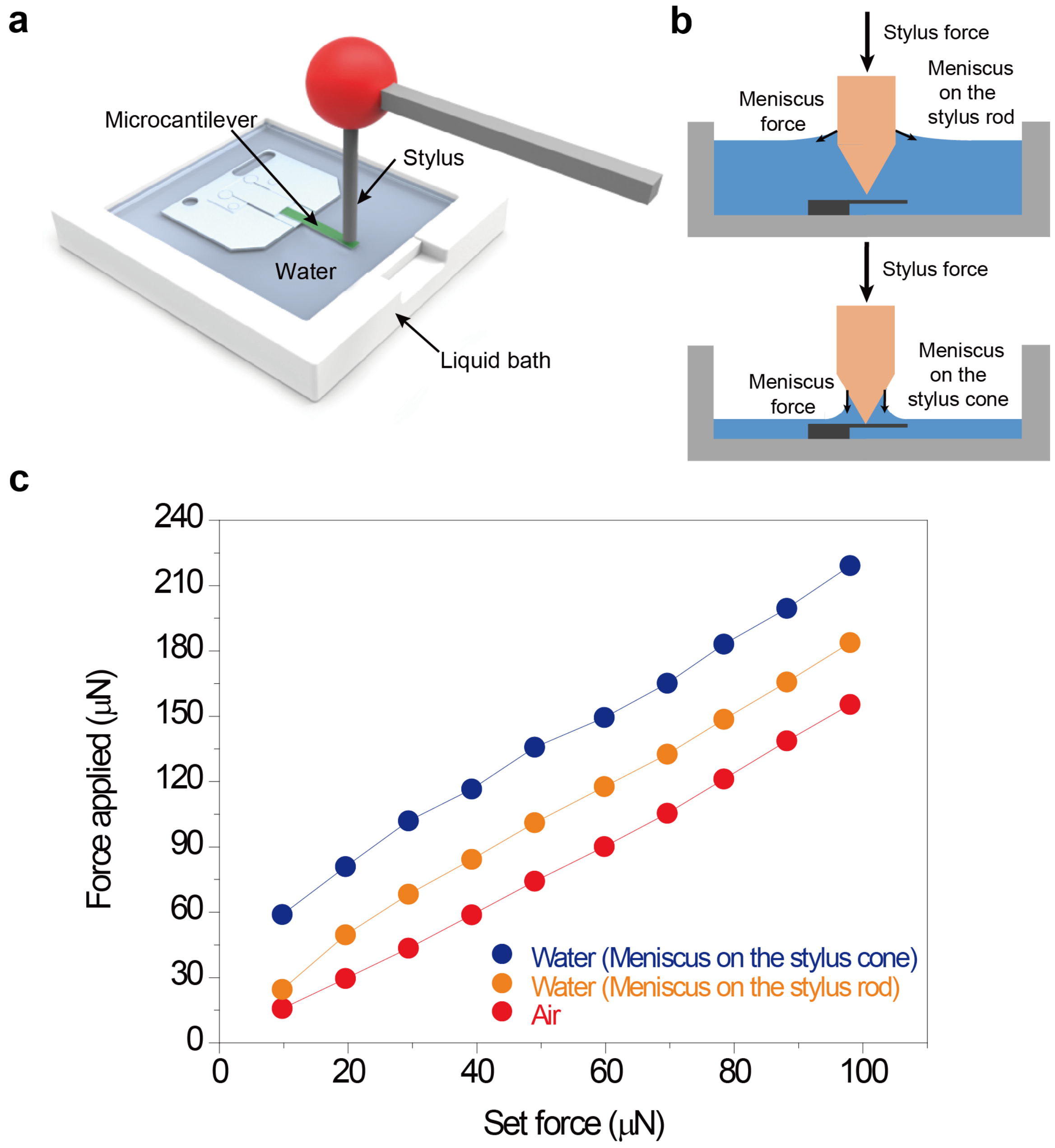

Supplementary Figure 10 | Force calibration for stylus profilometer measurements of PEG-DA hydrogel cantilevers in air and water. a, Schematic of stylus profilometer experiment for commercial silicon and hydrogel cantilevers. **b,** Force-free body diagram applied to a cantilever during stylus profiling in water. Water level relative to the position of the stylus affects the direction of the meniscus force, thereby changing the overall applied forces to the cantilever. Meniscus forces are applied downward regardless of the water level. **c,** Relationship between the force setpoint and the measured force applied to a commercial silicon cantilever during stylus profiling in air and water with different menisci formed.

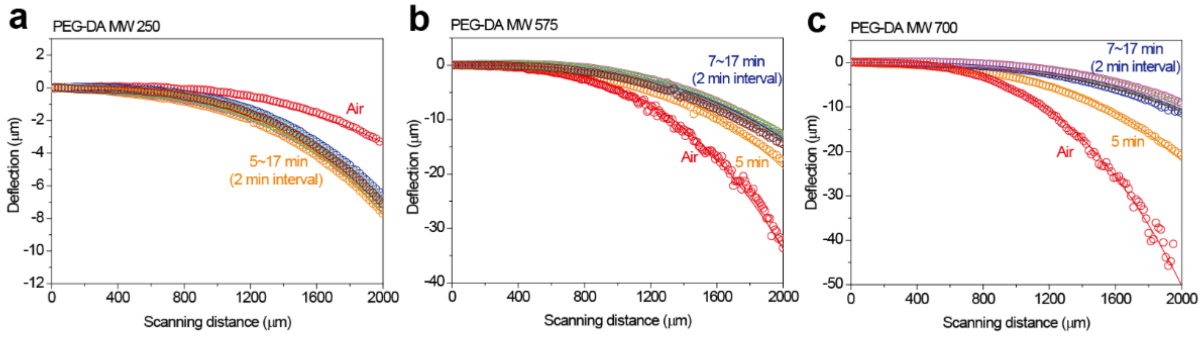

Supplementary Figure 11 | Deflection traces of PEG-DA hydrogel cantilevers from stylus profilometer measurements in air and water demonstrating transient responses due to partial swelling. a, Deflection traces of a hydrogel (PEG-DA MW 250) cantilever in air and water at 5-17 min after immersion. Similar deflection traces are presented for other cantilever types: **b,** hydrogel (PEG-DA MW 575) cantilever and **c,** hydrogel (PEG-DA MW 700) cantilever.

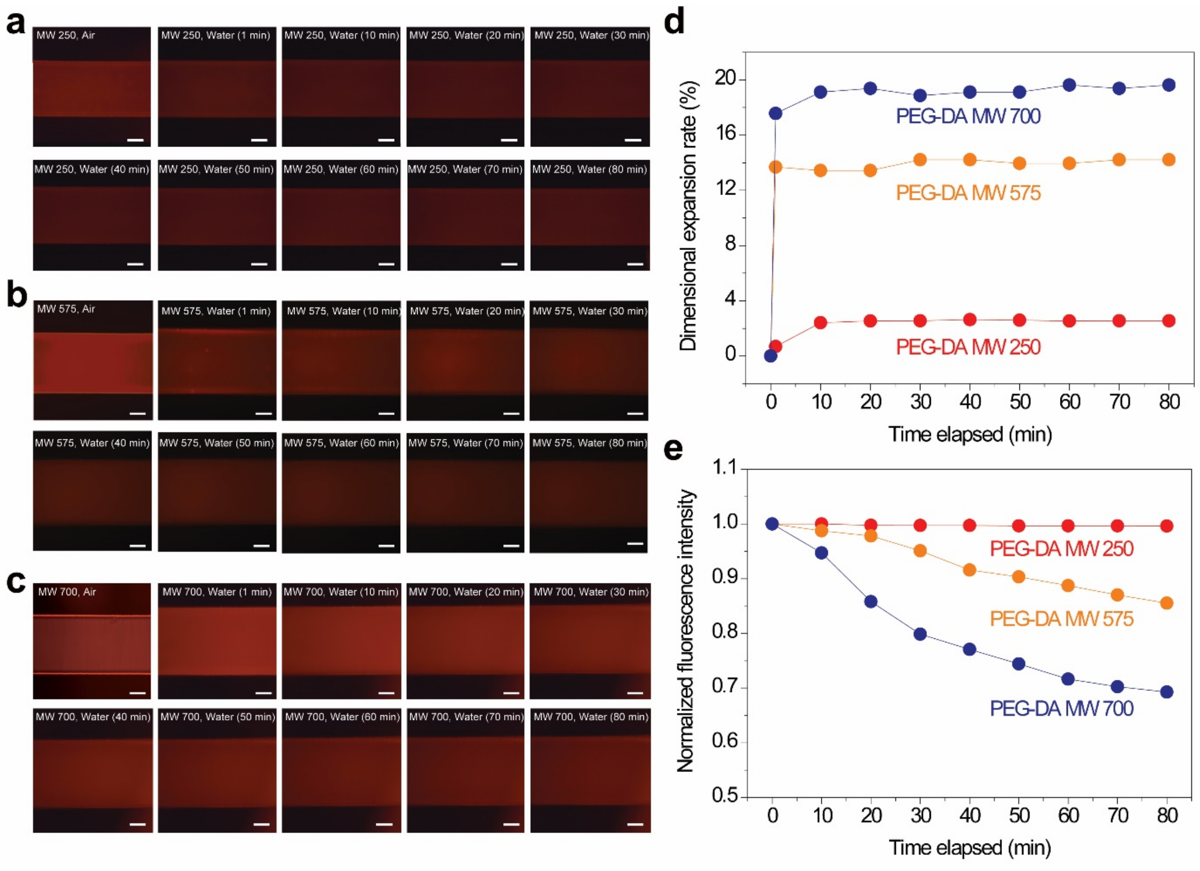

Supplementary Figure 12 | Water-induced swelling of PEG-DA hydrogels and fluorescence intensity change due to dye diffusion. a-c, Fluorescence microscope images of PEG-DA MW 250 (a), MW 575 (b), and MW 700 (c) hydrogel cantilevers (length, width, and thickness: 2000, 350, and 100 μm) in air and water. Linear expansion of the hydrogel cantilever structure due to swelling and a decrease in fluorescence intensity due to loss of fluorescent dye molecules by diffusion were observed. All images were taken from the top, showing the widths of the cantilevers. All scale bars are 100 μm. **d,** Water-induced swelling expansion rate as a function of dwell time in water. **e,** Variation in fluorescence intensity as a function of dwell time in water.

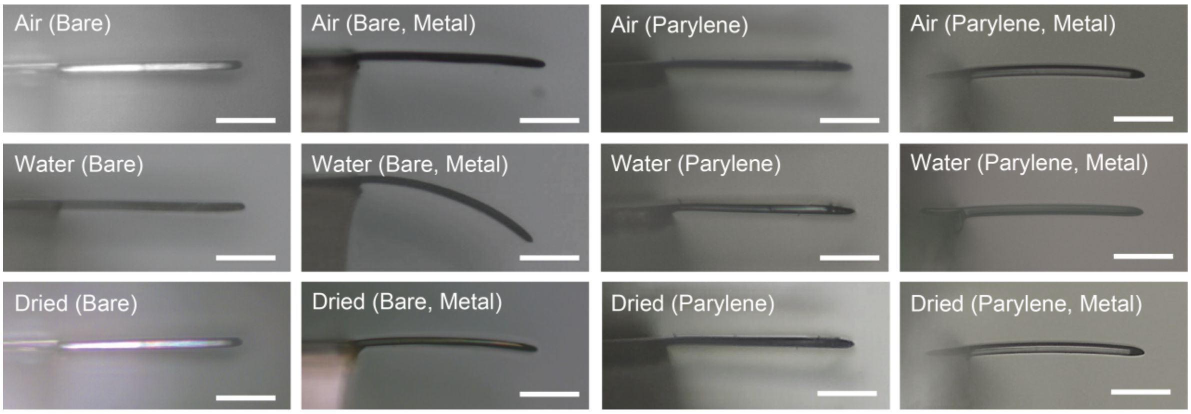

Supplementary Figure 13 | Effect of parylene coating on swelling-induced bending of PEG-DA hydrogel cantilevers. Side view optical microscope images of PEG-DA hydrogel cantilevers in sequential order: air, water, and air again after drying. From left to right, the columns present four types of hydrogel cantilevers: bare, metal-deposited, parylene-deposited, and parylene and metal sequentially deposited. Thicknesses of the parylene and metal coating were 1 μm and 120 nm, respectively. Parylene was deposited on all sides of the cantilever, while metal was deposited on the top side only. All scale bars are 100 μm.

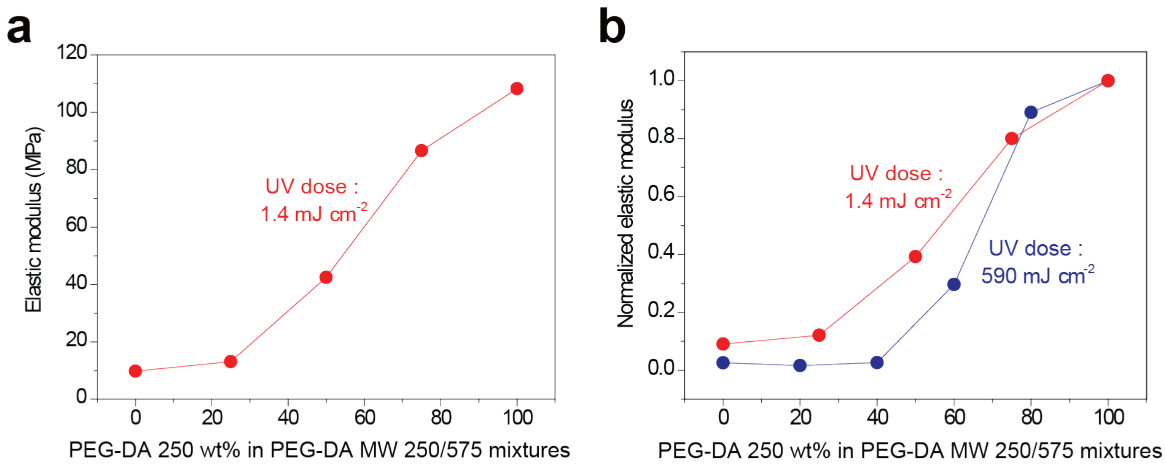

Supplementary Figure 14 | Elastic moduli of PEG-DA MW 250/575 mixtures in air. a, Elastic moduli of PEG-DA 250/575 mixtures obtained by stylus profilometer measurements. A UV light dose of 1.4 mJ cm^{-2} was used. Elastic moduli of PEG-DA MW 250 and MW 575 were measured to be 110 and 10 MPa, respectively. **b,** Elastic moduli of PEG-DA 250/575 mixtures at two different UV doses (1.4 and 590 mJ cm^{-2}) normalized by values of 100% PEG-DA MW 250.

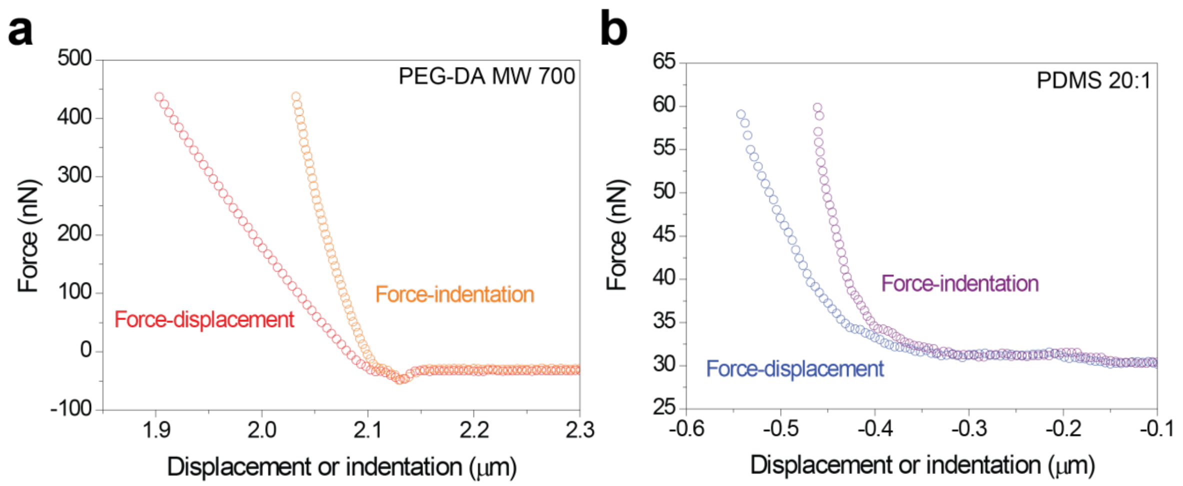

Supplementary Figure 15 | Conversion from force-displacement to force-indentation curves. a-b, Force-displacement and converted force-indentation curves for PEG-DA MW 700 substrate in air (a) and PDMS 20:1 substrate in water (b).

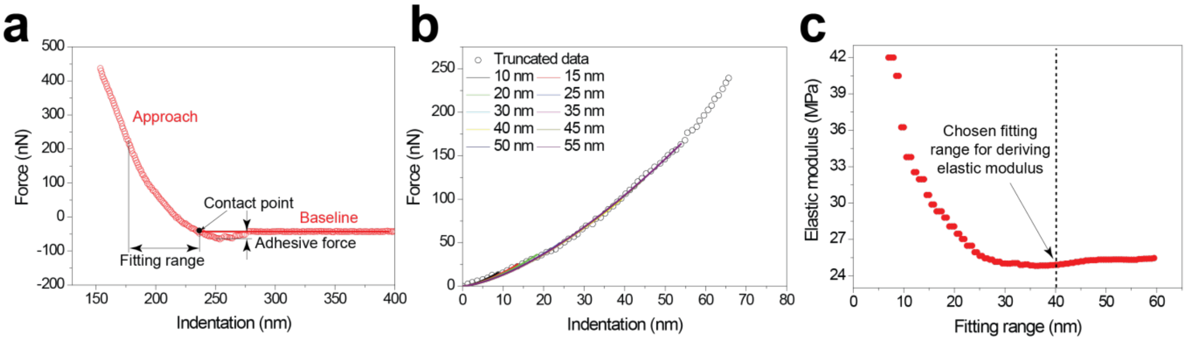

Supplementary Figure 16 | Criteria for determining the contact point in *F-d* measurements conducted on a PEG-DA MW 700 substrate in air and protocols for deriving the elastic modulus. a, Approach portion of the force indentation curve on a PEG-DA MW 700 substrate and determination of the contact point by intersecting the approach curve with the extended linear fit of the baseline. **b,** Truncated force indentation curve with respect to the chosen contact point and various Hertz fit results obtained by increasing the fitting range. **c,** Derived elastic modulus dependent on the fitting range. The fitting range is determined to be ~40 nm beyond which the elastic modulus reaches a constant value.

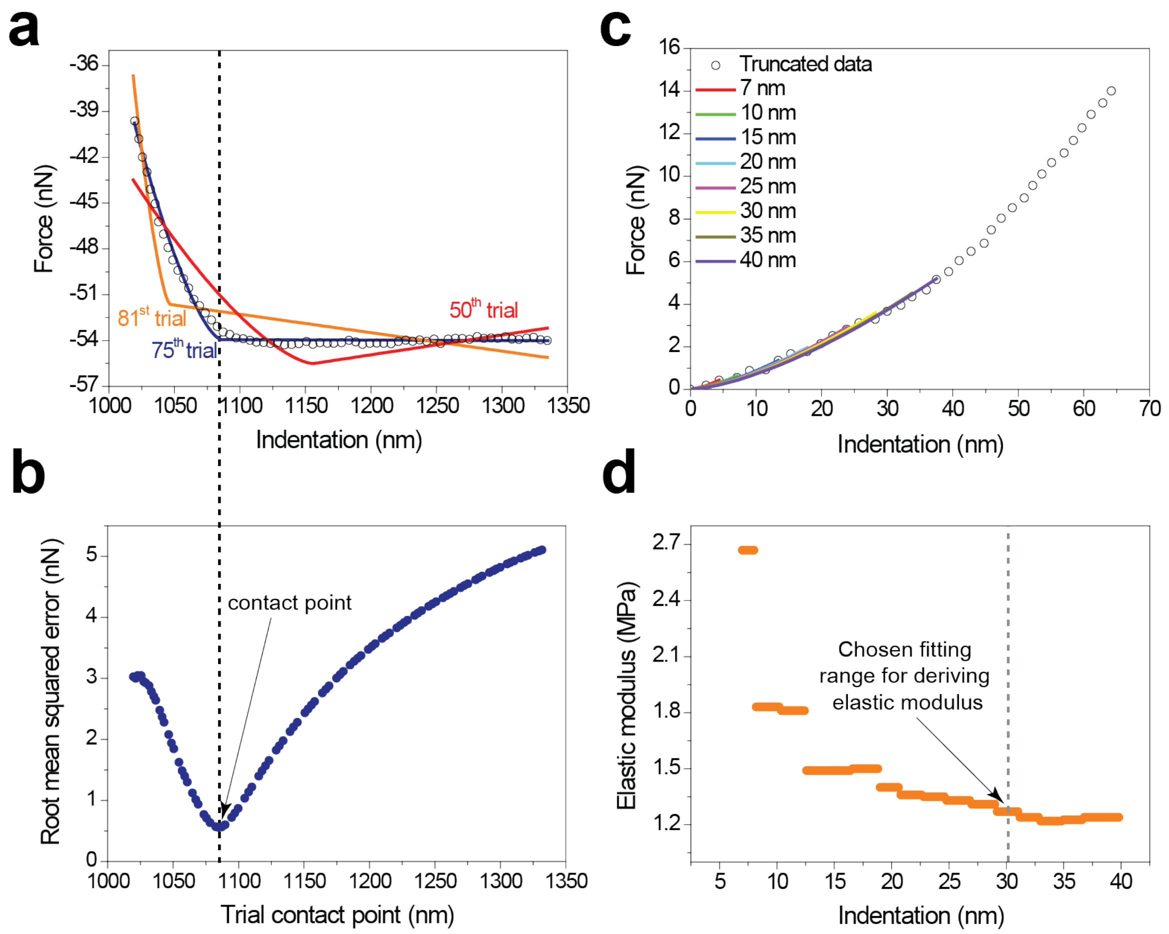

Supplementary Figure 17 | Criteria for determining the contact point in *F-d* measurements conducted on a PDMS 20:1 substrate in water and protocols for deriving the elastic modulus. **a,** Truncated region of interest in the force-indentation data for a PDMS 20:1 substrate with infinite thickness in water, and procedure for identifying the contact point using the equation (18). **b,** Calculated root mean squared error for each trial of contact point. **c,** Truncated force indentation curve with respect to the chosen contact point and various Hertz fit results obtained by increasing the fitting range. **d,** Derived elastic modulus dependent on the fitting range. The fitting range is determined to be ~30 nm beyond which the elastic modulus reaches a constant value.

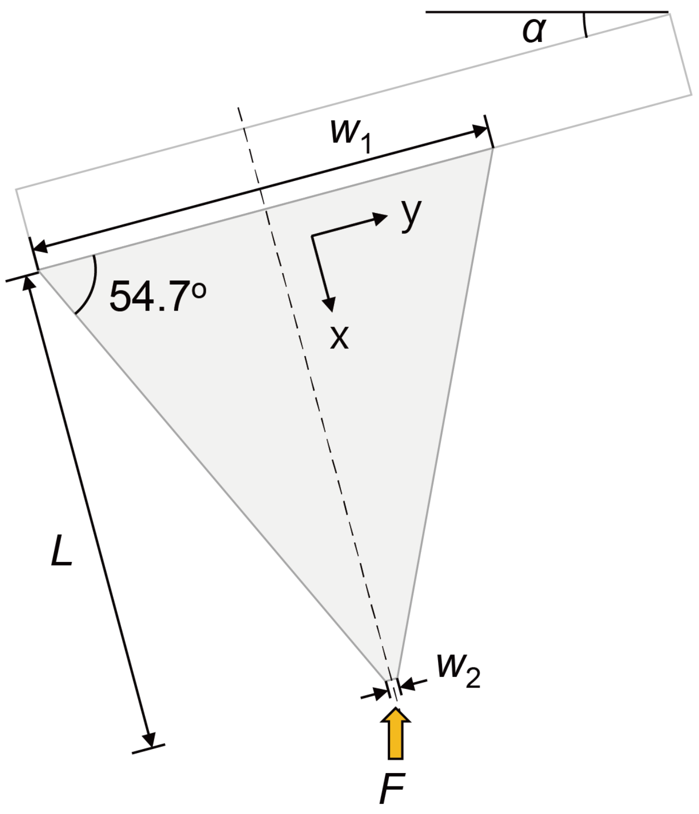

Supplementary Figure 18 | Simplified tip model for estimation of the lateral tip deformation. Simplified model for calculation of the lateral deformation of a hydrogel tip. The cross-sectional area at the center of the tip was shown. w_1, w_2, L, and α are widths of tip base and apex, tip height, and tilt angle, respectively.

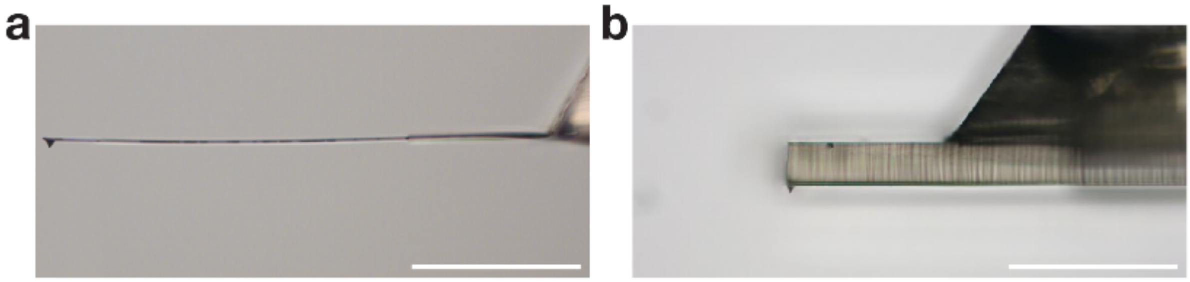

Supplementary Figure 19 | Optical micrographs of the lowest and highest spring constant hydrogel nano-probes. a, PEG-DA MW 700 hydrogel nano-probe (length, width, and thickness: 1500, 35, and 5 μm, k=0.00003 N m^{-1}). **b,** PEG-DA MW 250 hydrogel nano-probe (length, width, and thickness: 500, 350, and 100 μm, k=1000 N m^{-1}). Scale bars are 500 μm.

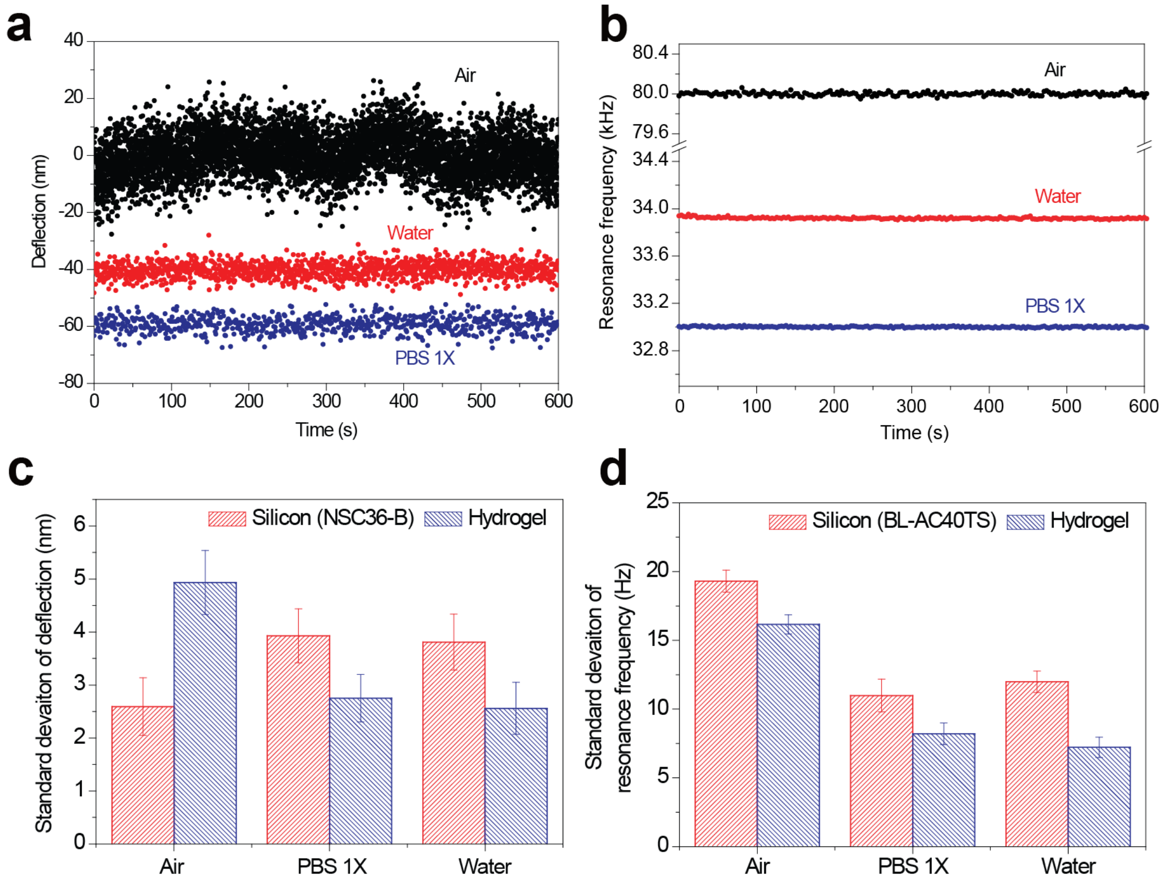

Supplementary Figure 20 | Static deflection and resonance frequency fluctuations of hydrogel cantilevers showing stability characteristics in comparison with silicon cantilevers. a, Static deflection of one of three type #1 hydrogel cantilevers (length, width, and thickness: 240, 50, and 10 μm) in air, water, and PBS solution. The data are offset for clarity. **b,** Resonance frequency of one of three type #2 hydrogel cantilevers (length, width, and thickness: 220, 50, and 20 μm) in air, water, and PBS solution. **c,** Standard deviations of the static deflection of three hydrogel (type #1; length, width, and thickness: 240-250, 50, and 10 μm) and three silicon cantilevers (NSC36-B, Mikromasch) in air, water, and PBS solution. **d,** Standard deviations of the resonance frequency of three hydrogel (type #2; length, width, and thickness: 200-220, 50, and 20 μm) and three silicon cantilevers (BL-AC40TS, Olympus) in air, water, and PBS solution.

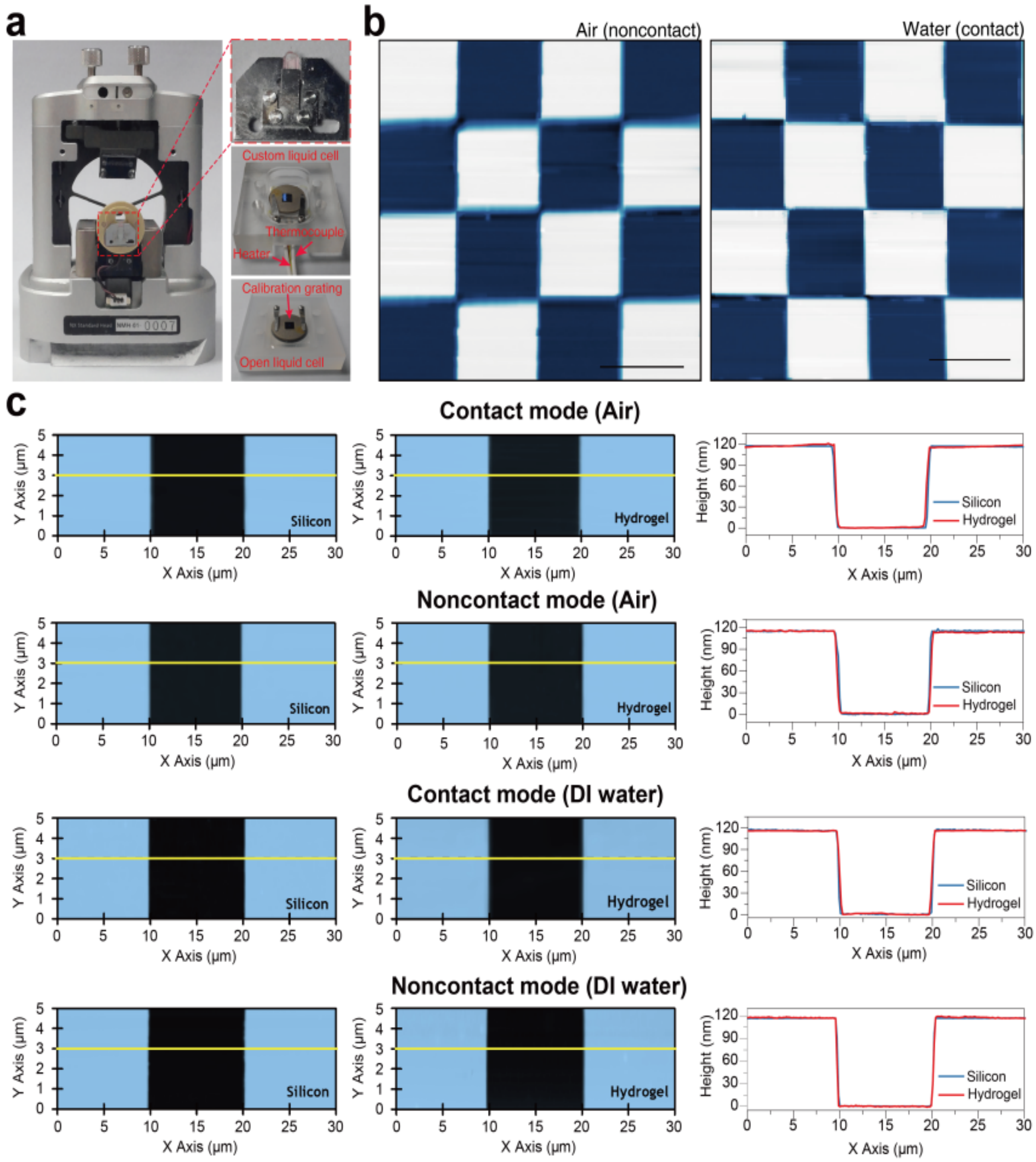

Supplementary Figure 21 | Contact and noncontact mode imaging with hydrogel AFM nano-probes. a, A hydrogel AFM nano-probe was mounted on the liquid probehand of the NX10 AFM instrument (Park Systems) (left and top right). A calibration grating mounted in custom-built (middle right) or commercial (bottom right) liquid cell is immersed in water. **b,** Noncontact mode and contact mode topographic imaging results for a 150 nm tall silicon grating (TGX11P, MikroMasch) in air (left) and water (right), respectively by a hydrogel AFM probe (length, width, and thickness: 240, 50, and 20 μm). Scale bars are 5 μm. **c,** Direct comparison of silicon and hydrogel AFM probe performance. Topographical imaging with contact mode in air (top), noncontact mode in air (the second from top), contact mode in water (the third from top), and noncontact mode in water (bottom) for a 120-nm deep calibration grating (HS-100MG, Budget sensors) obtained by commercial silicon AFM probes (PPP-CONTSCR for contact mode and PPP-NCHR for noncontact mode, Nanosensors) (left) and by a fabricated hydrogel AFM probe (length, width, and thickness: 320, 50, and 15 μm) (center). Line scans (right) compare results at y=3 μm (yellow lines).

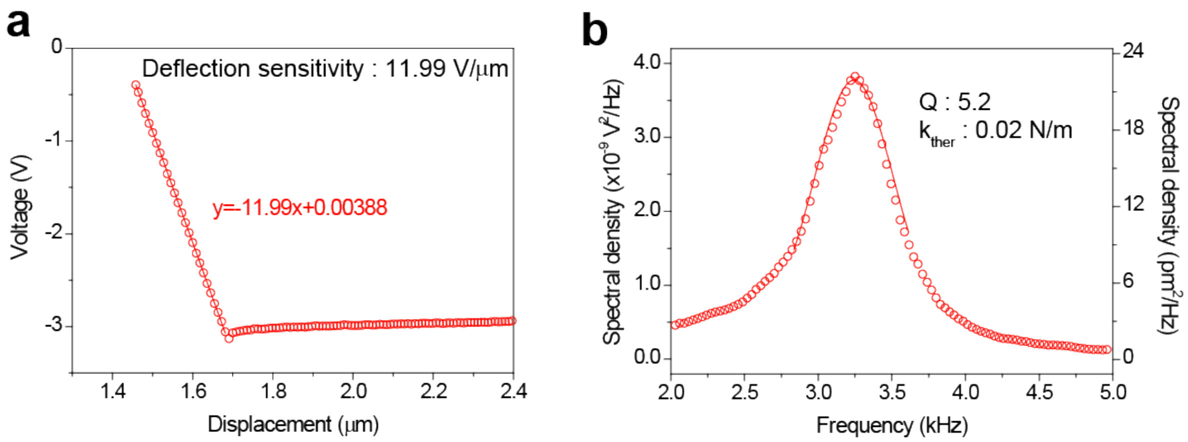

Supplementary Figure 22 | Characterization of a soft hydrogel nano-probe. a, *F*-*d* measurement data using a soft hydrogel nano-probe on a freshly cleaved mica substrate. **b,** Thermomechanical noise spectrum of the soft hydrogel nano-probe.

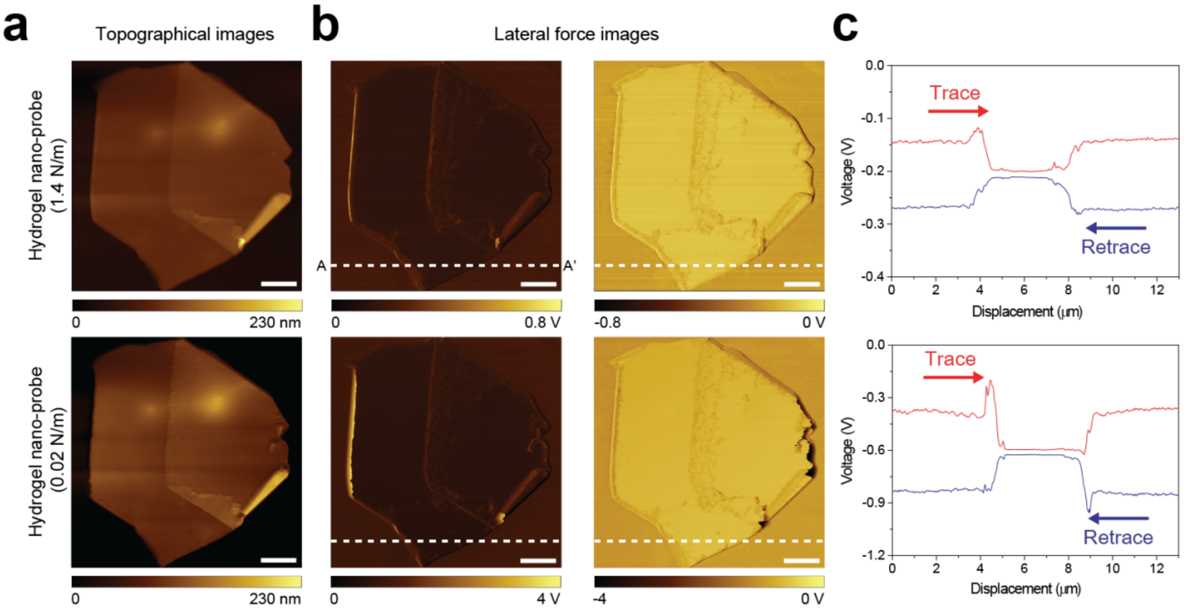

Supplementary Figure 23 | Friction imaging application of soft hydrogel nano-probes. a-b, Comparison of AFM (a) and LFM (b) images of graphitic layers on a silicon substrate obtained with hydrogel nano-probes with different spring constants (1.4 and 0.02 N m^{-1}). **c,** Line scans of LFM images along lines A-A'. All scale bars are 2 μm.

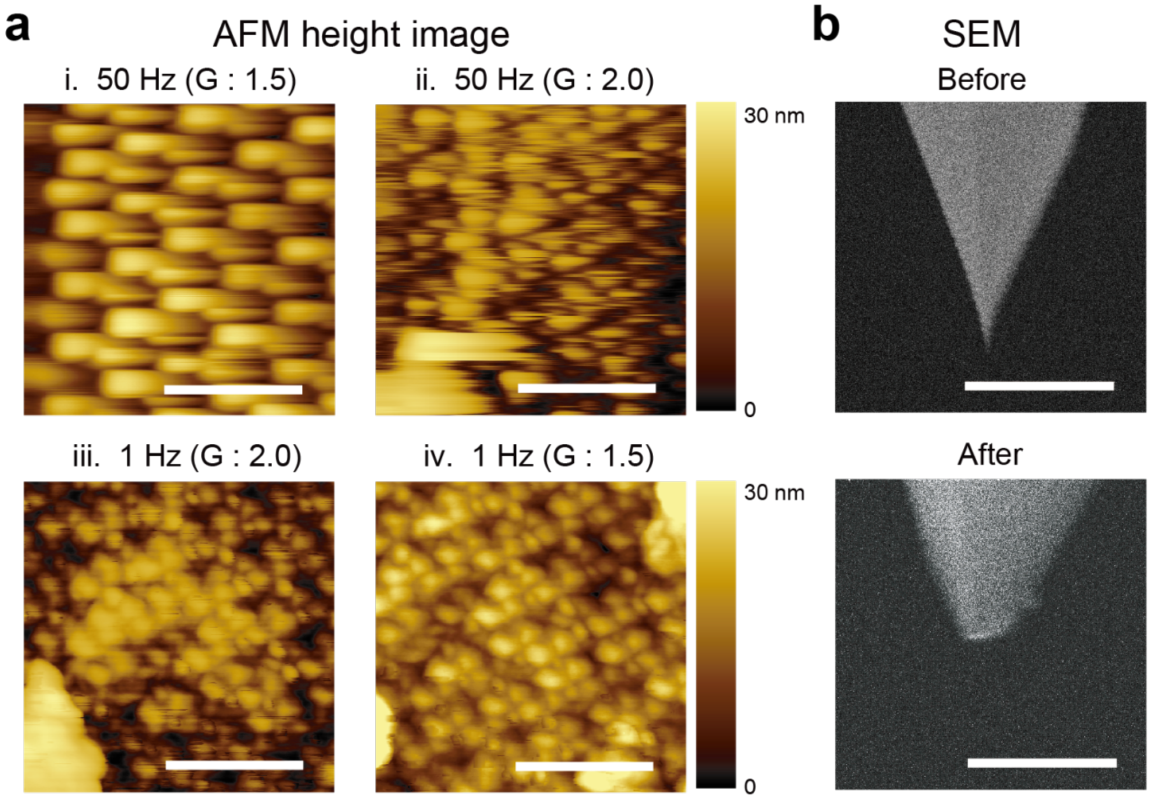

Supplementary Figure 24 | Noncontact mode high speed imaging with a commercial silicon probe showing the image distortion and degradation. a, Sequential AFM height images taken at 50, 50, 1, and 1 Hz with z-servo gains of 1.5, 2.0, 2.0, and 2.0, respectively. All scale bars are 100 nm. **b,** SEM images of the silicon probe before and after the noncontact mode high speed imaging. Scale bars are 1 μm.

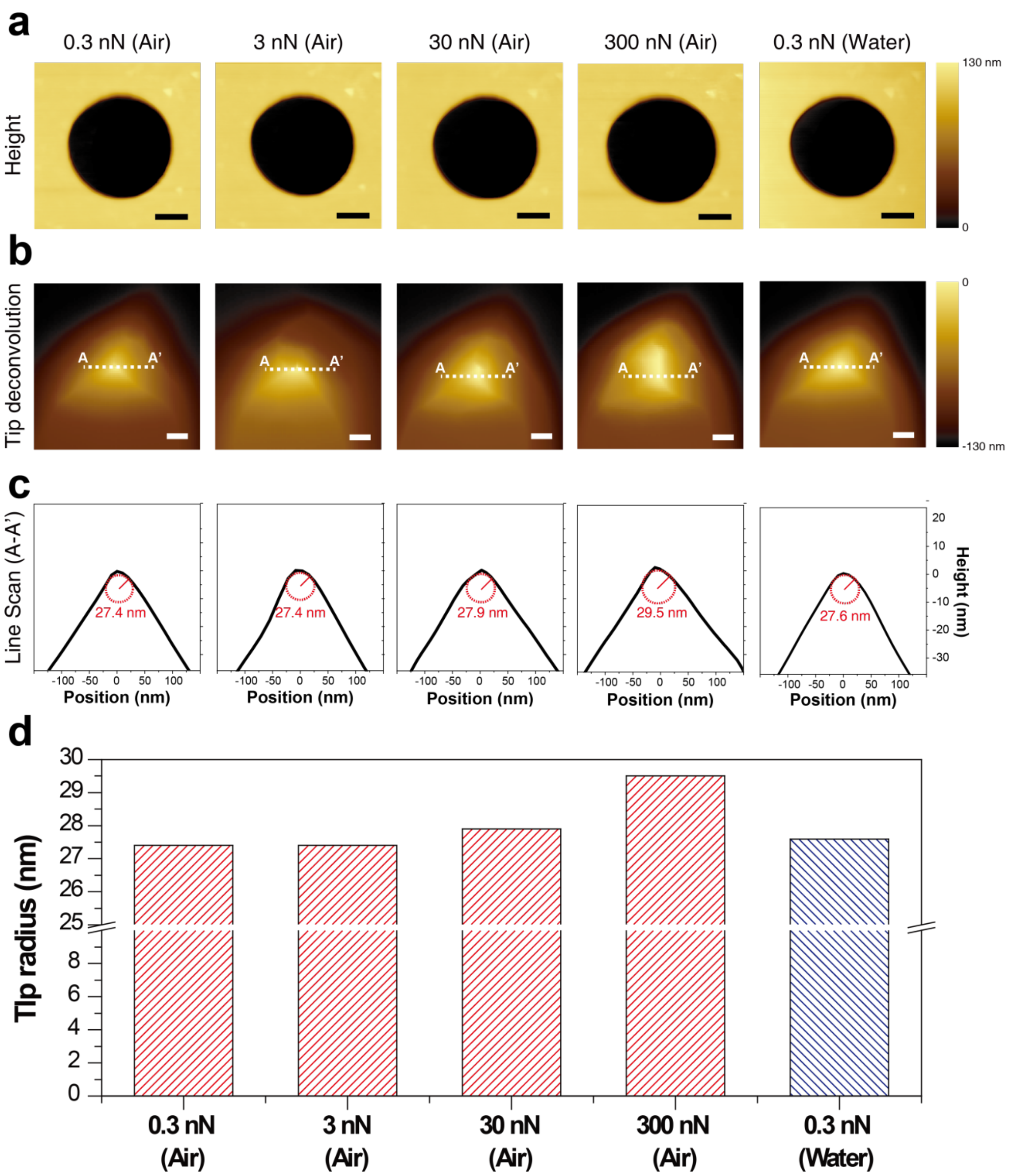

Supplementary Figure 25 | Effect of contact force and swelling on the tip radius of hydrogel nano-probes. a, Contact mode height images for a calibration grating sample in air and water. All scale bars are 1 µm. **b,** Deconvoluted tip images derived from the height images. All scale bars are 100 nm. **c,** Line scans along A-A'. **d,** Tip radius estimated from tip-sample deconvolution for different imaging conditions.

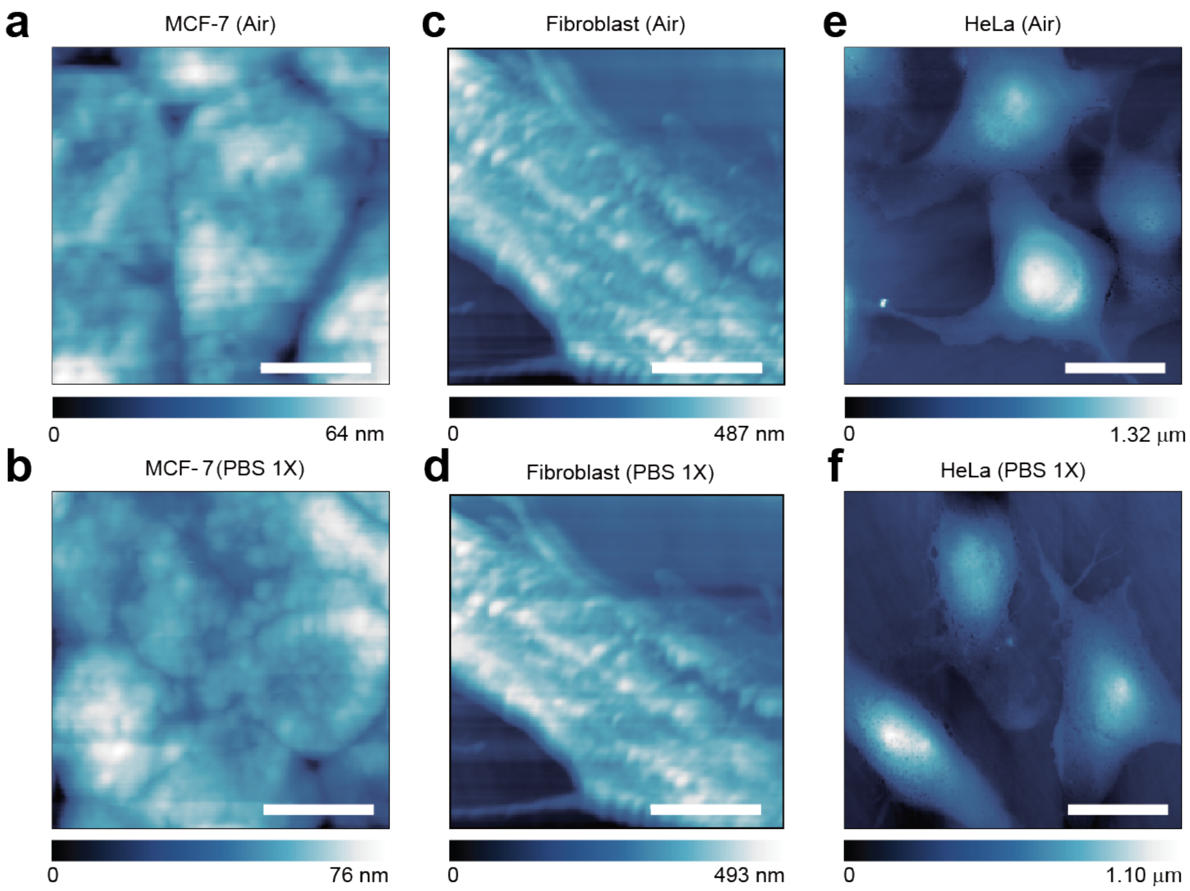

Supplementary Figure 26 | Morphological surface characterization of cells by hydrogel AFM nano-probes. a-b, Noncontact mode height images (length, width, and thickness: 260, 50, and 15 µm) of human breast cancer cells (MCF-7) in air (a) and PBS solution (b). Scale bars are 10 µm. **c-d,** Contact mode height images (length, width, and thickness: 360, 50, and 15 µm) of human fibroblast cells (MRC-5) in air (c) and PBS solution (d). Scale bars are 5 µm. **e-f,** Contact mode height images (length, width, and thickness: 360, 50, and 15 µm) of HeLa cells in air (e) and PBS solution (f). Scale bars are 20 µm.

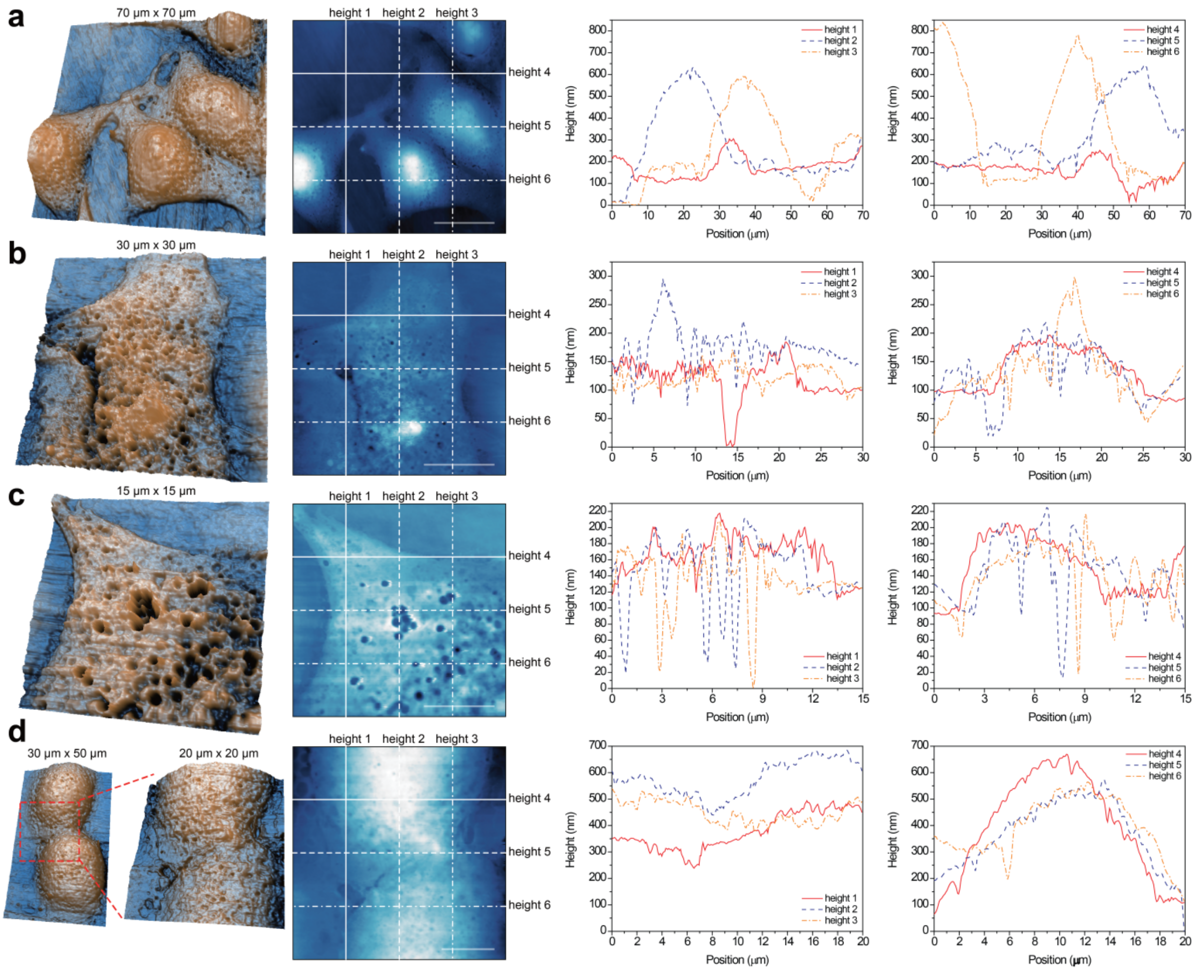

Supplementary Figure 27 | Representative three-dimensional AFM height data collected for HeLa cells in PBS. a-d, Microstructural features of the cell surface can be observed on the nanometer scale, as obtained by topographical imaging with a hydrogel nano-probe (length, width, and thickness: 360, 50, and 15 μm). Scale bars in 2D height images are 20, 10, 5, and 5 μm from (a) to (d), respectively.

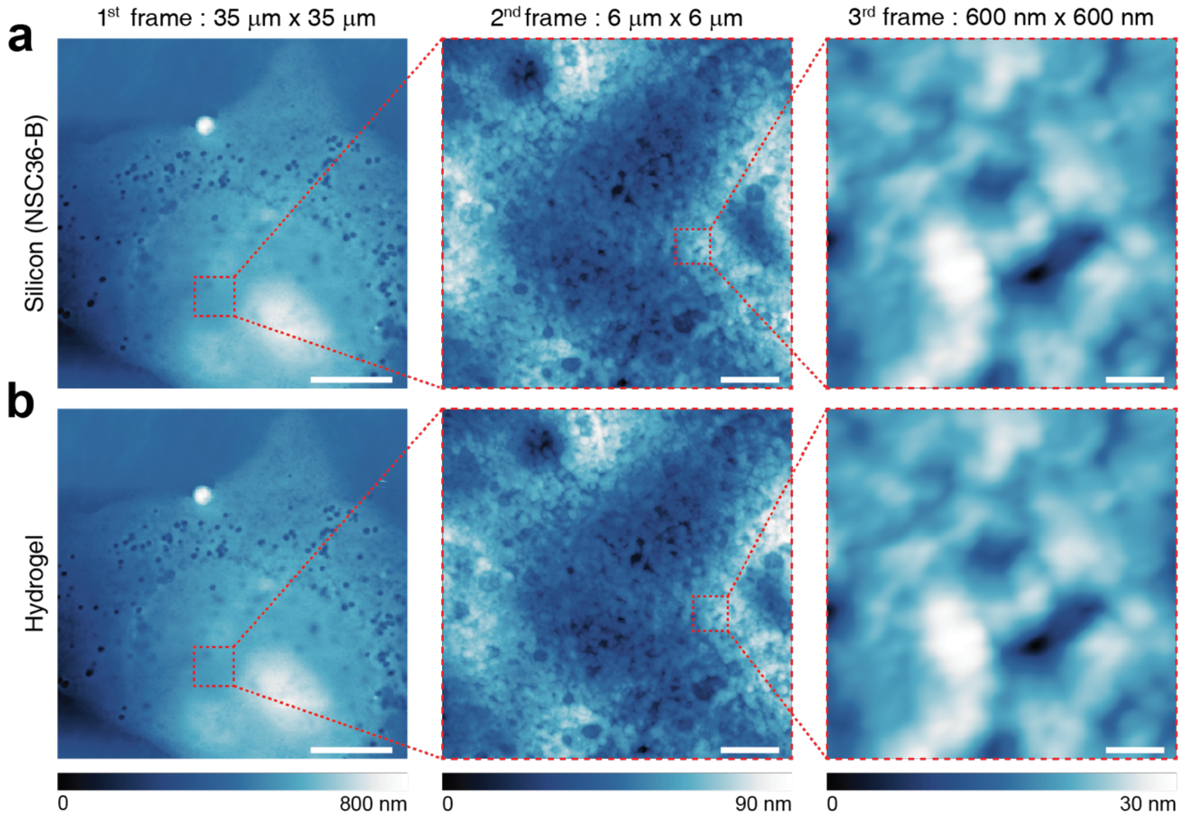

Supplementary Figure 28 | Contact mode AFM images on dried HeLa cells by fabricated hydrogel and commercial silicon probes. a, A commercial silicon probe (NSC36-B, Mikromasch) and **b,** fabricated hydrogel probe (length, width, and thickness: 210, 50, and 10 μm) are used. A scan rate of 0.3 Hz and contact force of 3 nN were used in all experiments. The scale bars are 7 μm (1st frame), 1 μm (2nd frame), and 100 nm (3rd frame), respectively. For the 600 nm × 600 nm region, similar image qualities are obtained with both probes.

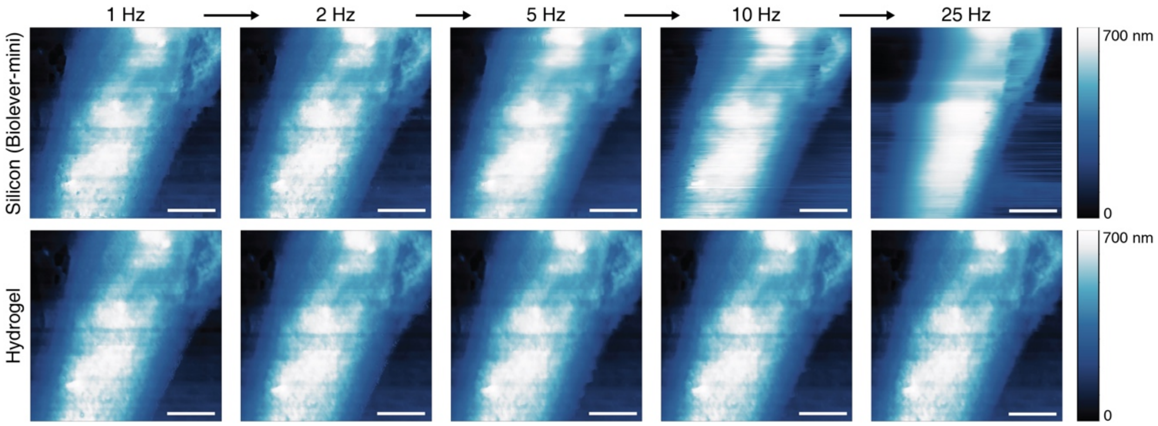

Supplementary Figure 29 | Performance comparison for noncontact mode high-speed cell imaging in liquid conditions by hydrogel versus commercial silicon AFM probes. Noncontact mode AFM height images of MRC-5 fixed cell imaged by a commercial silicon probe (Biolever-mini, Olympus) and hydrogel probe (length, width, and thickness: 220, 40, and 20 μm) with different scan rates of 1, 2, 5, 10, and 25 Hz, respectively. All scale bars are 10 μm.

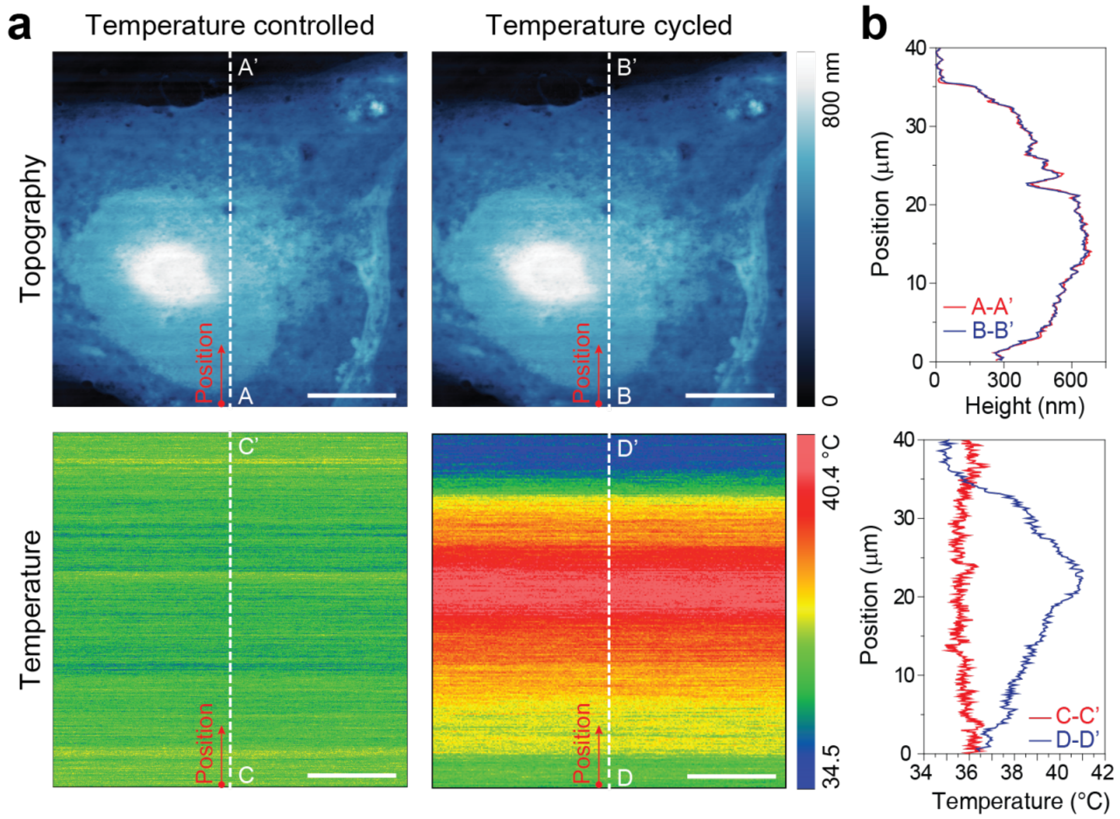

Supplementary Figure 30 | Noncontact mode AFM imaging with a hydrogel nano-probe under constant or modulated temperature conditions in PBS solution. a, Height (top) and temperature images (bottom) of an MRC-5 fibroblast cell by a hydrogel nano-probe under constant temperature around 36 °C (left) or cycled between 34.5 and 40.4 °C (right). **b,** Line scans of height and temperature along lines A-A', B-B', C-C', and D-D'. The vibration amplitude setpoint was 24 nm which is 80 % of the free vibration amplitude of 30 nm. All scale bars are 10 µm.

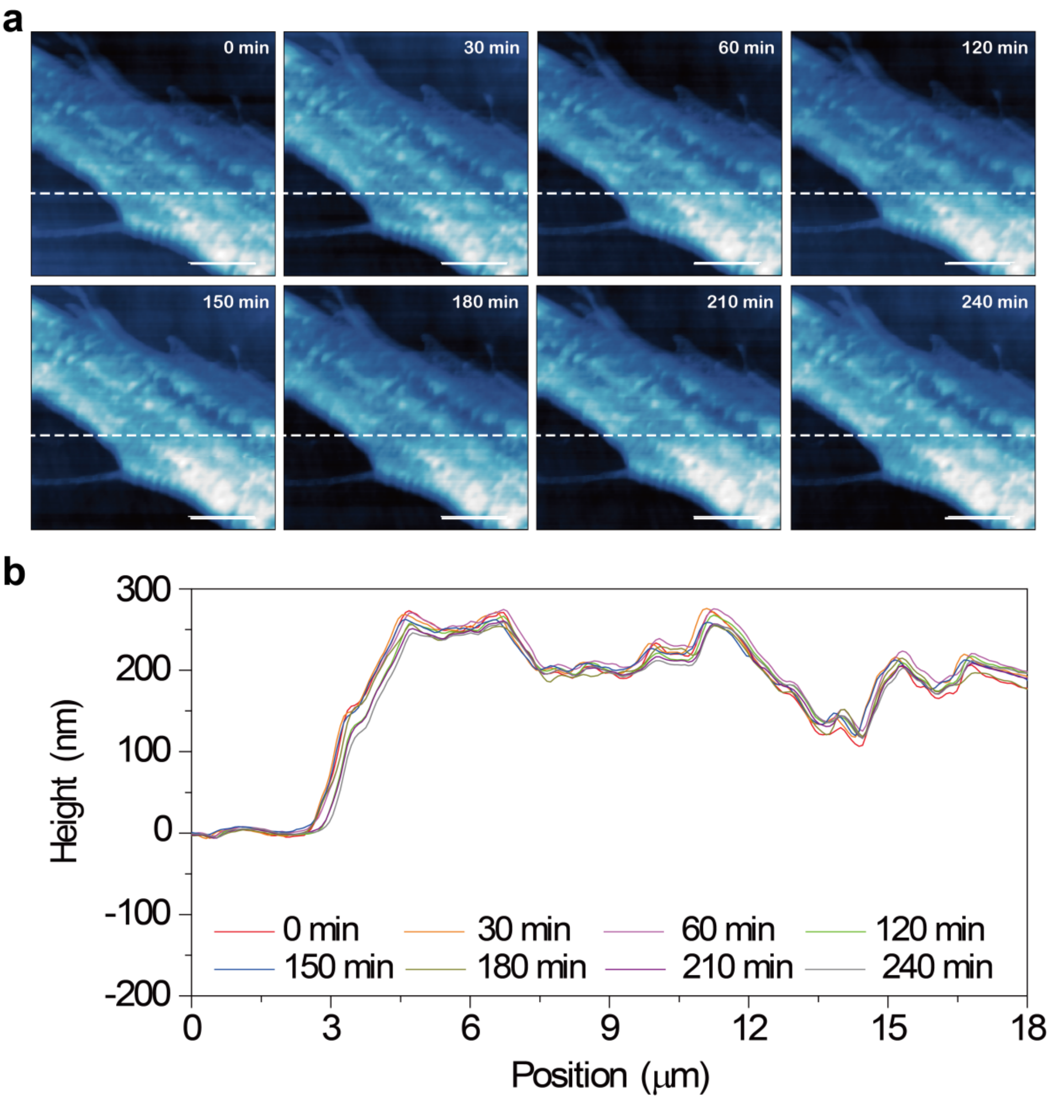

Supplementary Figure 31 | Time-lapse cell imaging with a hydrogel AFM nano-probe. In order to test probe durability and measurement reproducibility, contact mode imaging for human fibroblast cells (MRC-5) was repeated in saline conditions with a single hydrogel nano-probe (length, width, and thickness: 360, 50, and 15 μm) over 240 min. **a,** Height images at 0, 30, 60, 120, 150, 180, 210, and 240 min. All scale bars are 5 μm. **b,** Line scans compare results along the white dashed lines.

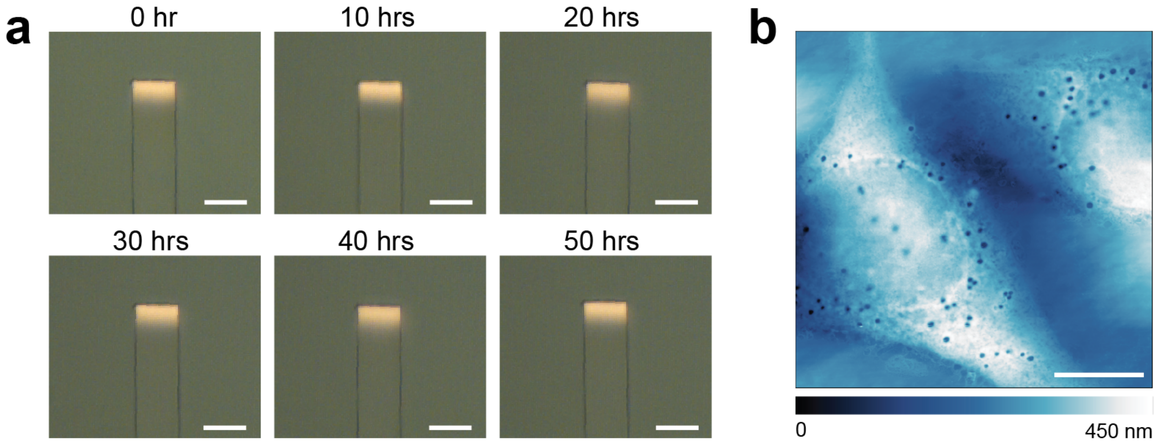

Supplementary Figure 32 | Visual investigation of long-term stability of the partial metal coating on the hydrogel nano-probe in liquid. a, Optical micrographs showing the stable partial gold coating in water taken at the interval of 10 hrs. All scale bars are 50 µm. **b,** Height image of HeLa cells taken in PBS by the hydrogel nano-probe with the partial metal coating incubated in water for 50 hrs before imaging. Scale bar is 10 µm.

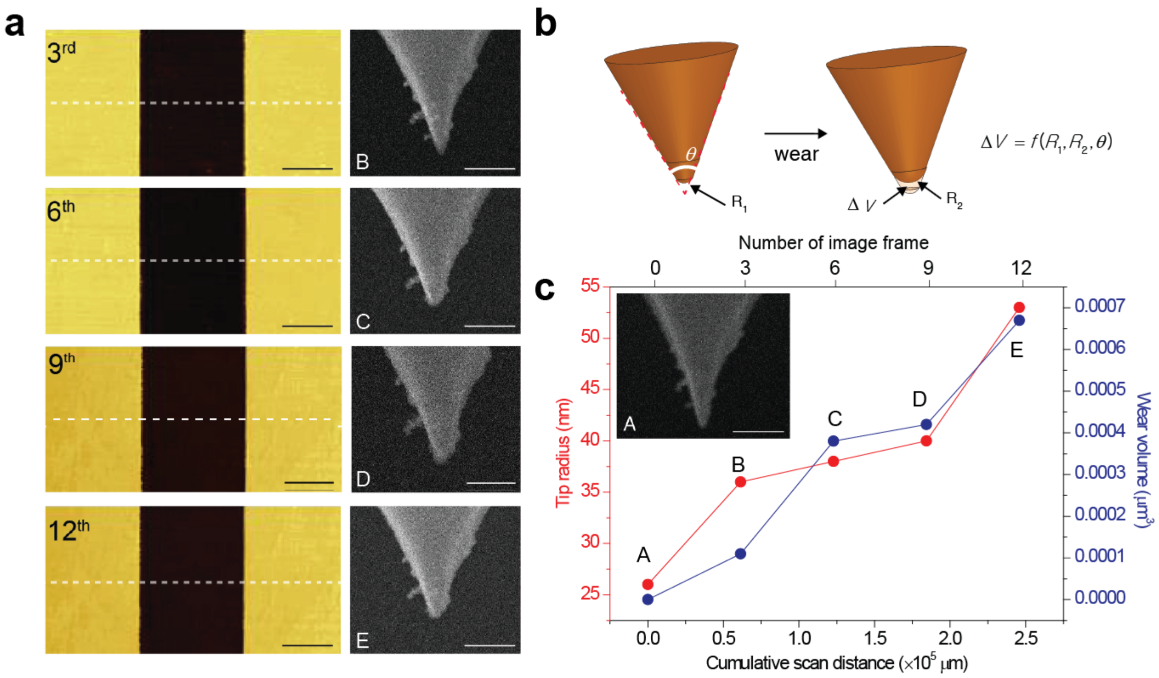

Supplementary Figure 33 | Wear characteristics of hydrogel nano-probes during contact mode imaging in PBS solution. a, Contact mode height images and SEM images taken at the 3rd, 6th, 9th, and 12th frames during repeated imaging of the calibration grating in PBS solution using hydrogel probe (length, width, and thickness: 300, 50, and 10 μm). Contact force of 3 nN and scan rate of 1 Hz was used. Scale bars are 5 μm and 500 nm for AFM and SEM images, respectively. **b,** Simplified hydrogel tip model as a truncated circular cone with a hemispherical cap. **c,** Tip radius and wear volume as a function of the cumulative scan distance. Inset shows the initial SEM image taken before the wear test. Scale bar is 500 nm.

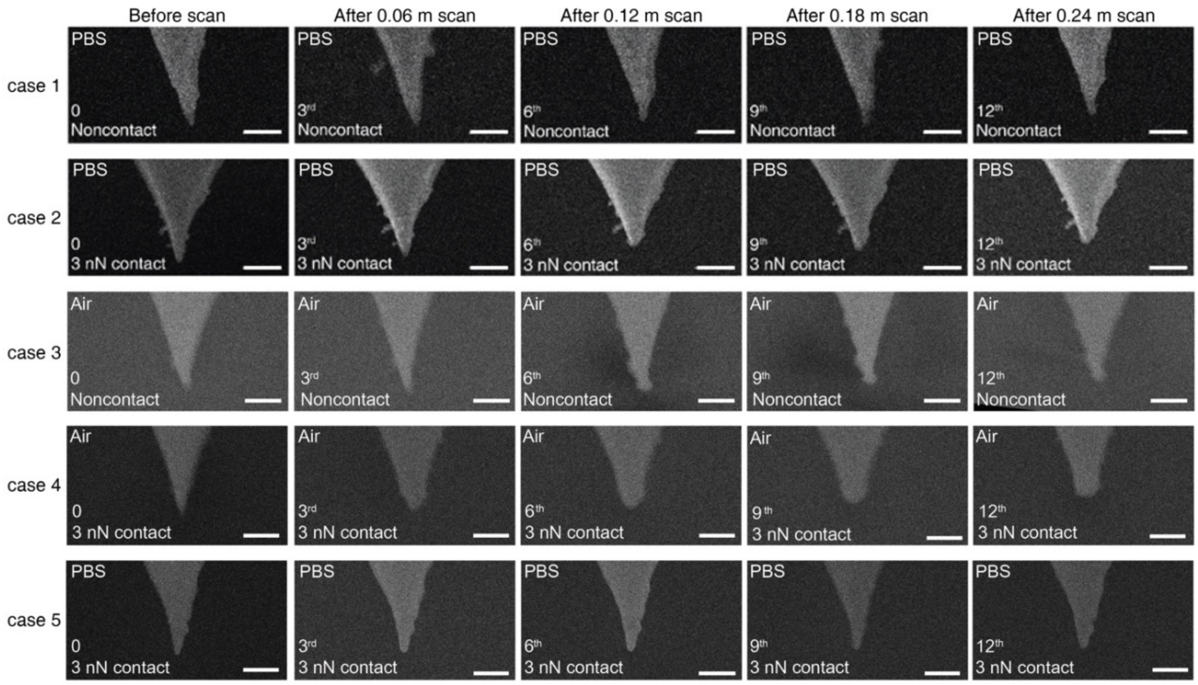

Supplementary Figure 34 | Sequential SEM images taken during durability testing. SEM images of hydrogel nano-tips (length, width, and thickness: 200, 50, and 10 μm for noncontact probes, and 300, 50, and 10 μm for contact probes) taken every 3 frames during repeated noncontact and contact mode imaging of the silicon and PDMS calibration grating samples in PBS solution and air. All scale bars are 500 nm. Total scan distance is calculated to be 0.246 m for each case.

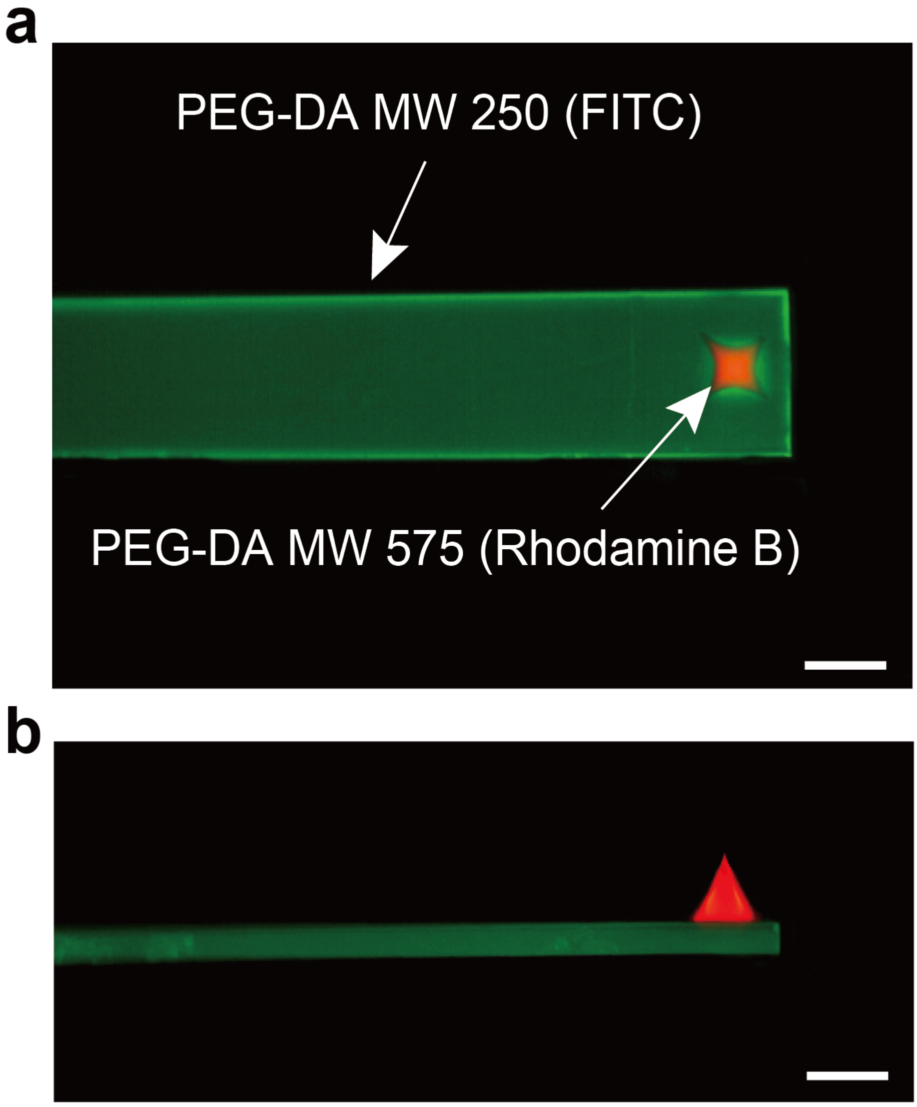

Supplementary Figure 35 | Hydrogel nano-probes with modular assembly. a-b, Top (a) and side (b) view of the composite hydrogel nano-probes with modular assembly. The beam structure consists of PEG-DA MW 250 mixed with FITC dye, and the tip structure has PEG-DA MW 575 mixed with Rhodamine B dye. Scale bars are 25 μm.

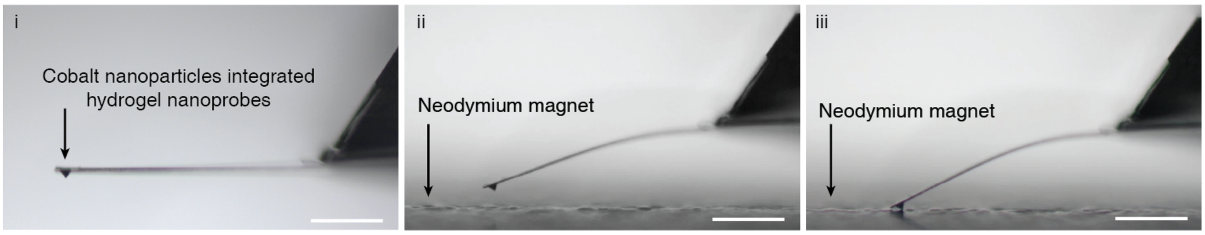

Supplementary Figure 36 | Magnetic field-induced deflection of a hydrogel nano-probe with cobalt nanoparticles encapsulated within the tip. A hydrogel nano-probe with encapsulated cobalt nanoparticles is straight when it is far away from a permanent neodymium magnet (i). The hydrogel nano-probe is deflected when it approaches towards a neodymium magnet (ii). The hydrogel nano-probe eventually collapses and makes contact with the magnet since the magnetic force is larger than the restoring force of the cantilever spring (iii). All scale bars are 200 μm.

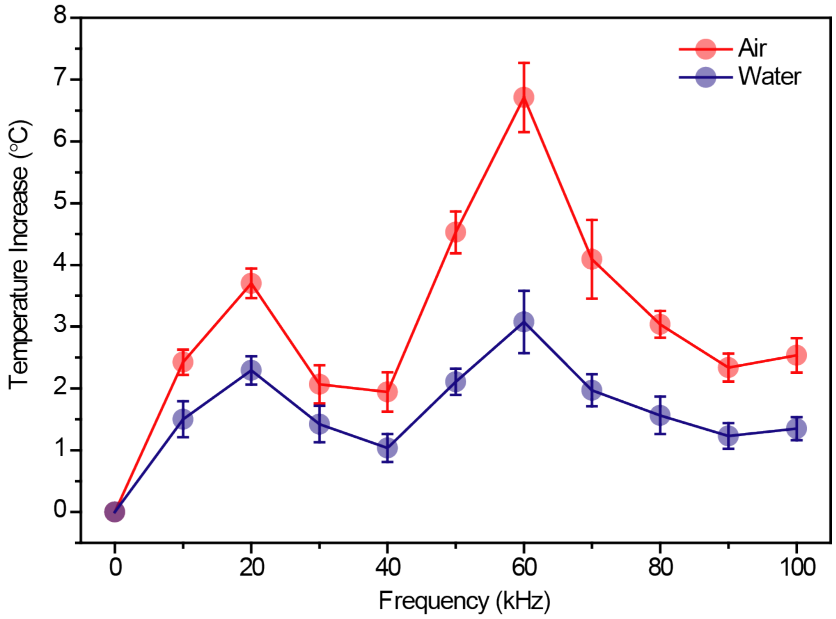

Supplementary Figure 37 | Frequency-dependent inductive heating. Temperature rise of the hydrogel nano-probe with the magnetic tip as a function of the frequency in air and water. The applied power is fixed to be 50 W. For noncontact temperature measurements via quantum dot fluorescence quenching (see Supplementary Figs. 39-43), quantum dots and cobalt nanoparticles are co-encapsulated in the hydrogel tip.

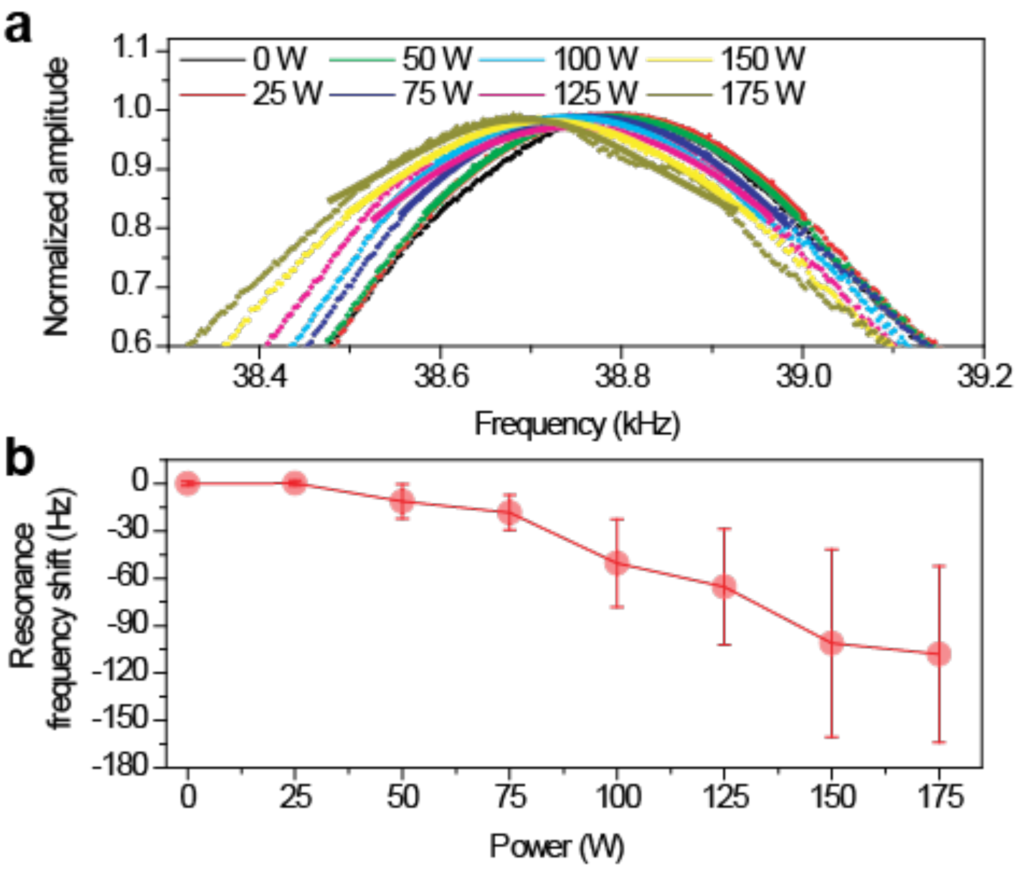

Supplementary Figure 38 | Resonance frequency shift of an inductively heated hydrogel nano-probe with the magnetic tip in air. a, Normalized amplitude spectra of a hydrogel nano-probe with a cobalt nanoparticle-encapsulated tip under induction heating in air. **b,** the resonance frequency shift as a function of the input power.

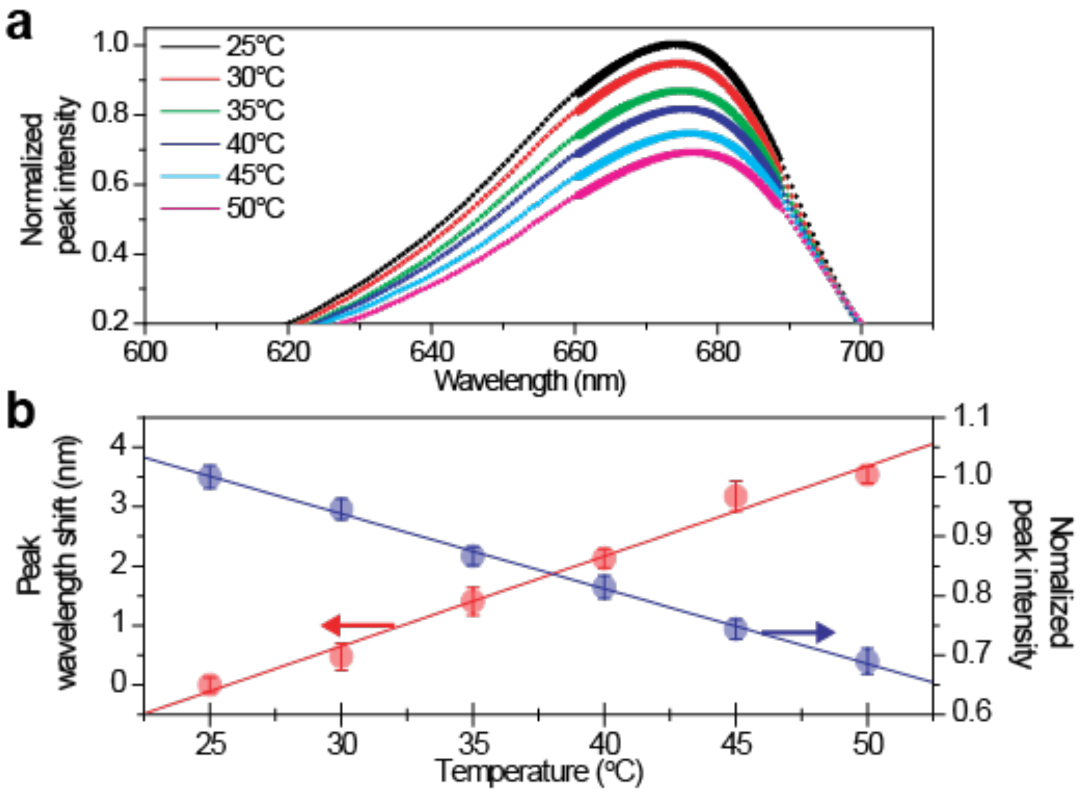

Supplementary Figure 39 | Fluorescence peak wavelength shift- and normalized peak intensity-temperature calibration of a hydrogel nano-probe with quantum dot-encapsulated tip in air. a, Normalized fluorescence spectra of a hydrogel nano-probe with encapsulated CdTe quantum dots which is in contact with the glass side of an ITO-coated glass substrate under Joule heating. **b**, the peak wavelength shift and the nomalized peak intensity as a function of temperature at the tip contact point in air.

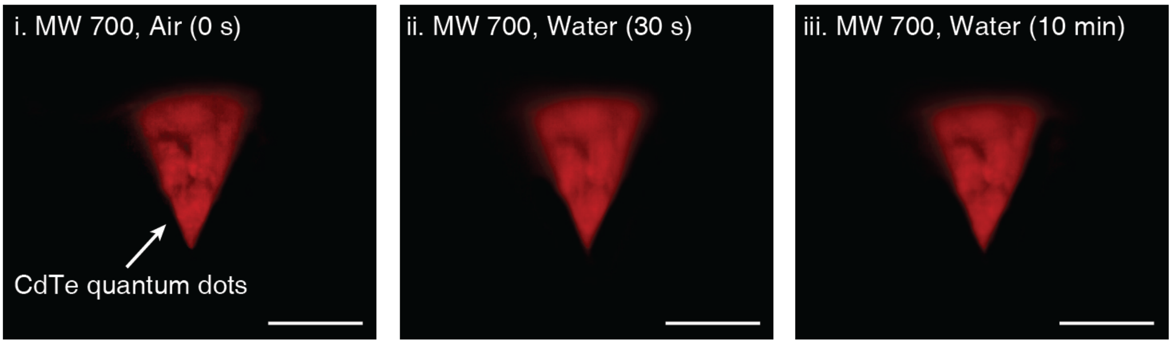

Supplementary Figure 40 | Hydrogel nano-probe with quantum dot-encapsulated tip in air and water. Fluorescence microscope images of a hydrogel nano-probe tip (PEG-DA MW 700) with CdTe quantum dots encapsulated at 0 s in air (i), 30 s (ii) and 10 min (iii) after immersion in deionized water showed similar and stable fluorescence intensities which are attributed to the larger diameter of quantum dots relative to the mesh size of the cured hydrogel. All scale bars are 20 μm.

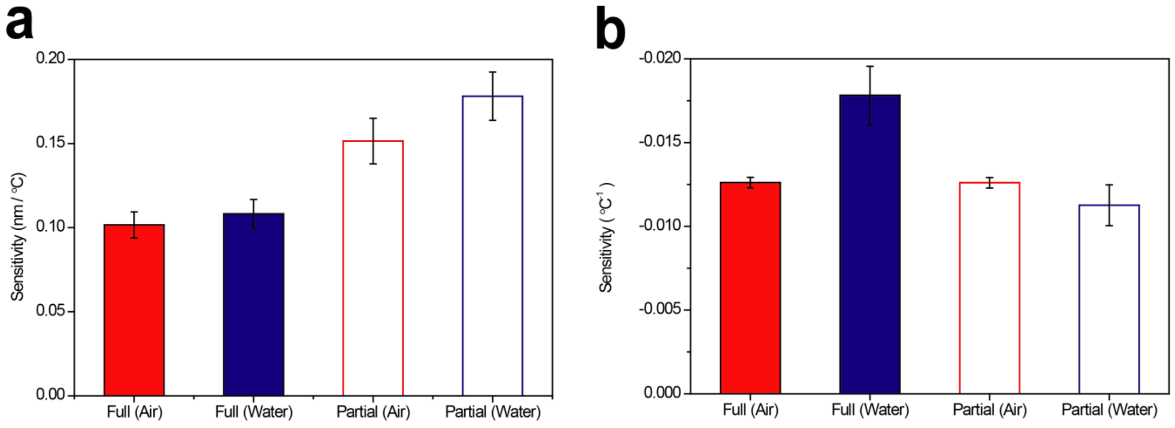

Supplementary Figure 41 | Temperature sensitivity of fluorescence peak wavelength shift and normalized peak intensity of hydrogel nano-probes with quantum dot-functionalized tip. a, Temperature sensitivity comparison for hydrogel nano-probes with CdTe quantum dots fully or partially encapsulated in the tip based on the peak wavelength shift measured in air and water. **b,** Temperature sensitivity comparison for hydrogel nano-probes with CdTe quantum dots fully or partially encapsulated in the tip based on the normalized peak intensity measured in air and water. Error bars represent standard errors for slopes in the linear regression for measurements.

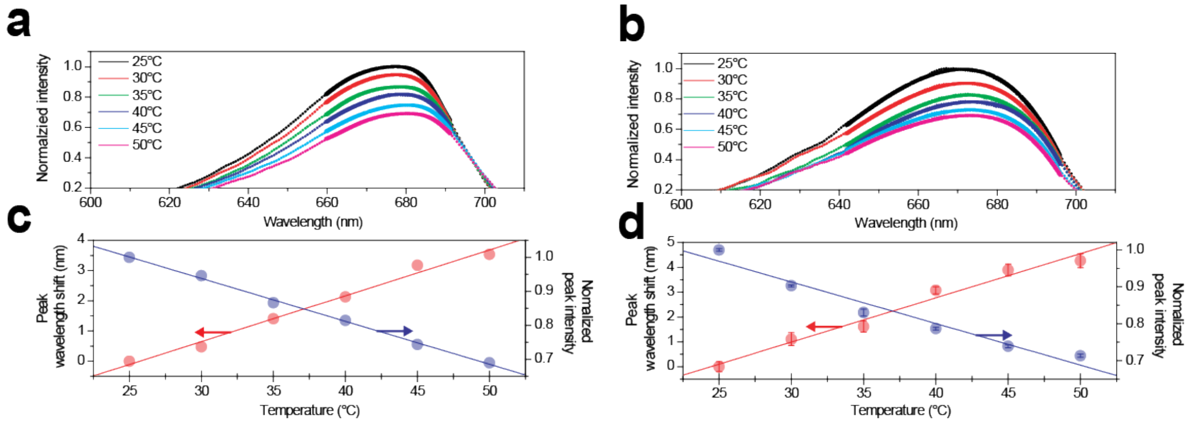

Supplementary Figure 42 | Temperature calibration of a hydrogel nano-probe with the dual-functional tip (co-encapsulated Co and CdTe nanoparticles) in air and water. a-b, Normalized intensity spectra of a hydrogel nano-probe with the dual-functional tip under induction heating in air (a) and water (b). **c-d,** Peak wavelength shift and the normalized peak intensity as a function of the local temperature at the contact point with the glass side of the ITO-coated glass substrate in air (c) and water (d).

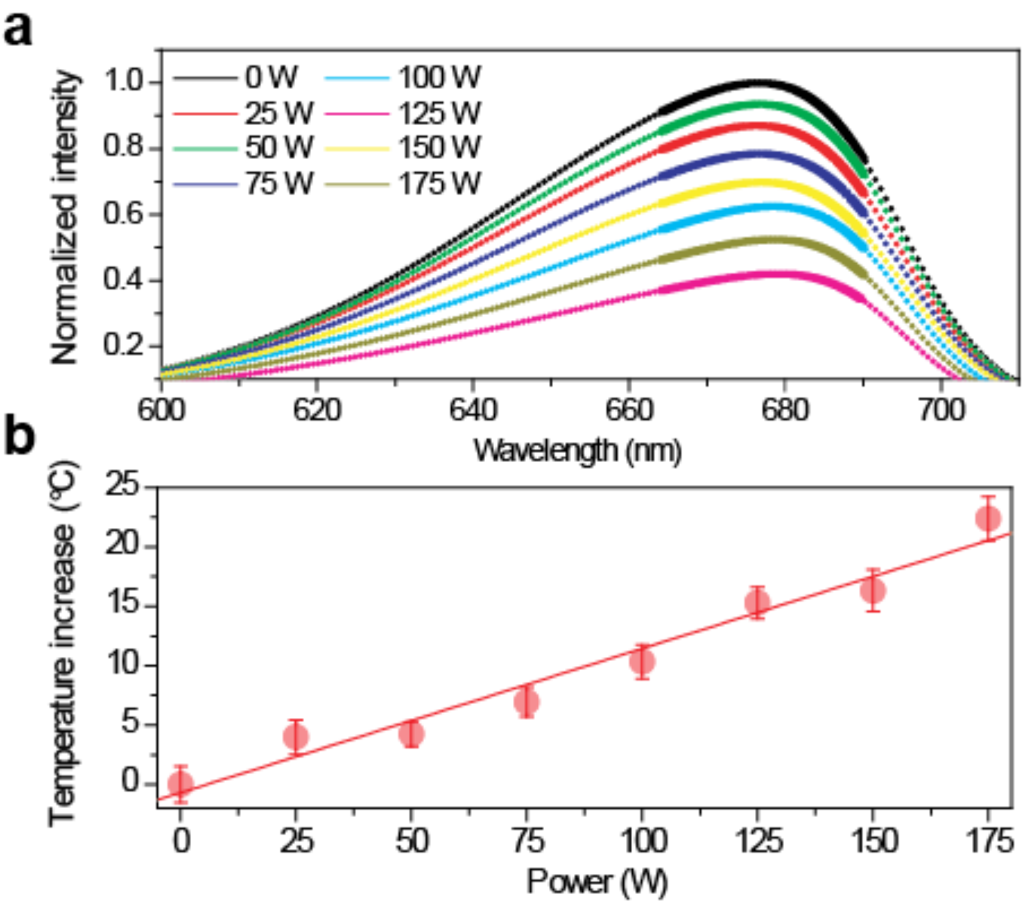

Supplementary Figure 43 | Dual function (inductive heating and temperature sensing) demonstration in air for a hydrogel nano-probe with co-encapsulated CdTe and Co nanoparticles. a, Fluorescence spectra from a hydrogel nano-probe with CdTe quantum dots and cobalt nanoparticles sequentially encapsulated under induction heating in air. **b,** Temperature increase measured by calibrated quantum dot fluorescence quenching as a function of the input power.

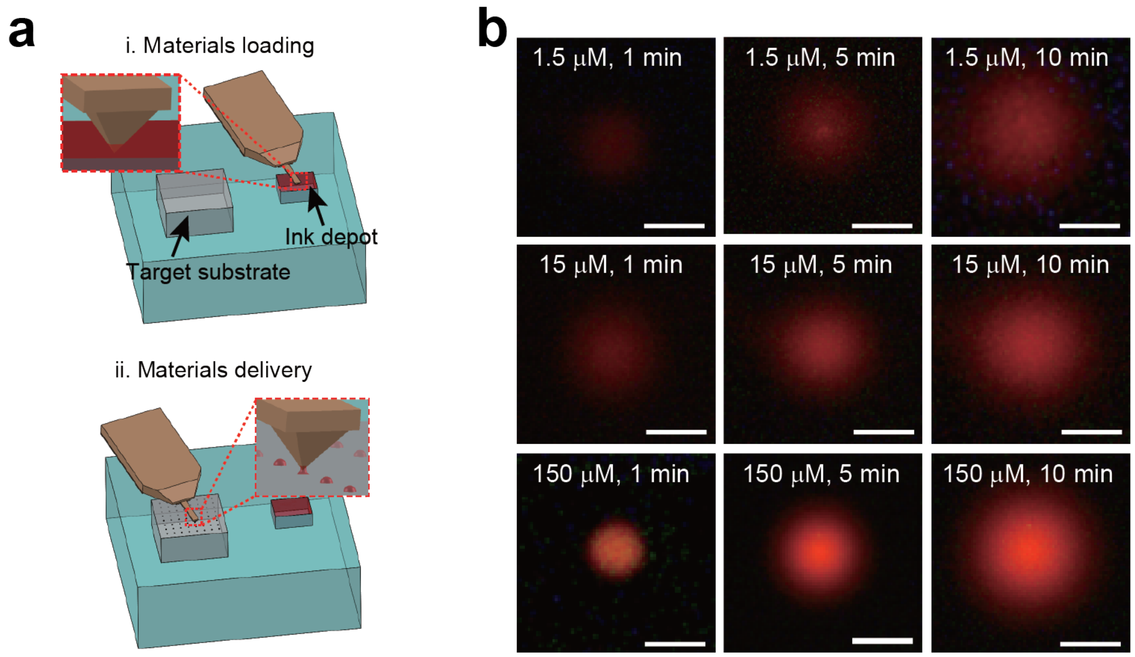

Supplementary Figure 44 | Experimental measurement of Rhodamine B delivery. a, Experimental schematic for localized materials loading (i) and delivery (ii). **b,** Effect of sample concentration and contact time on the efficiency of localized materials delivery. Rhodamine B dye-loaded hydrogel tips are used for delivery onto hydrated PEG-DA MW 700 target substrates. All scale bars are 5 µm.

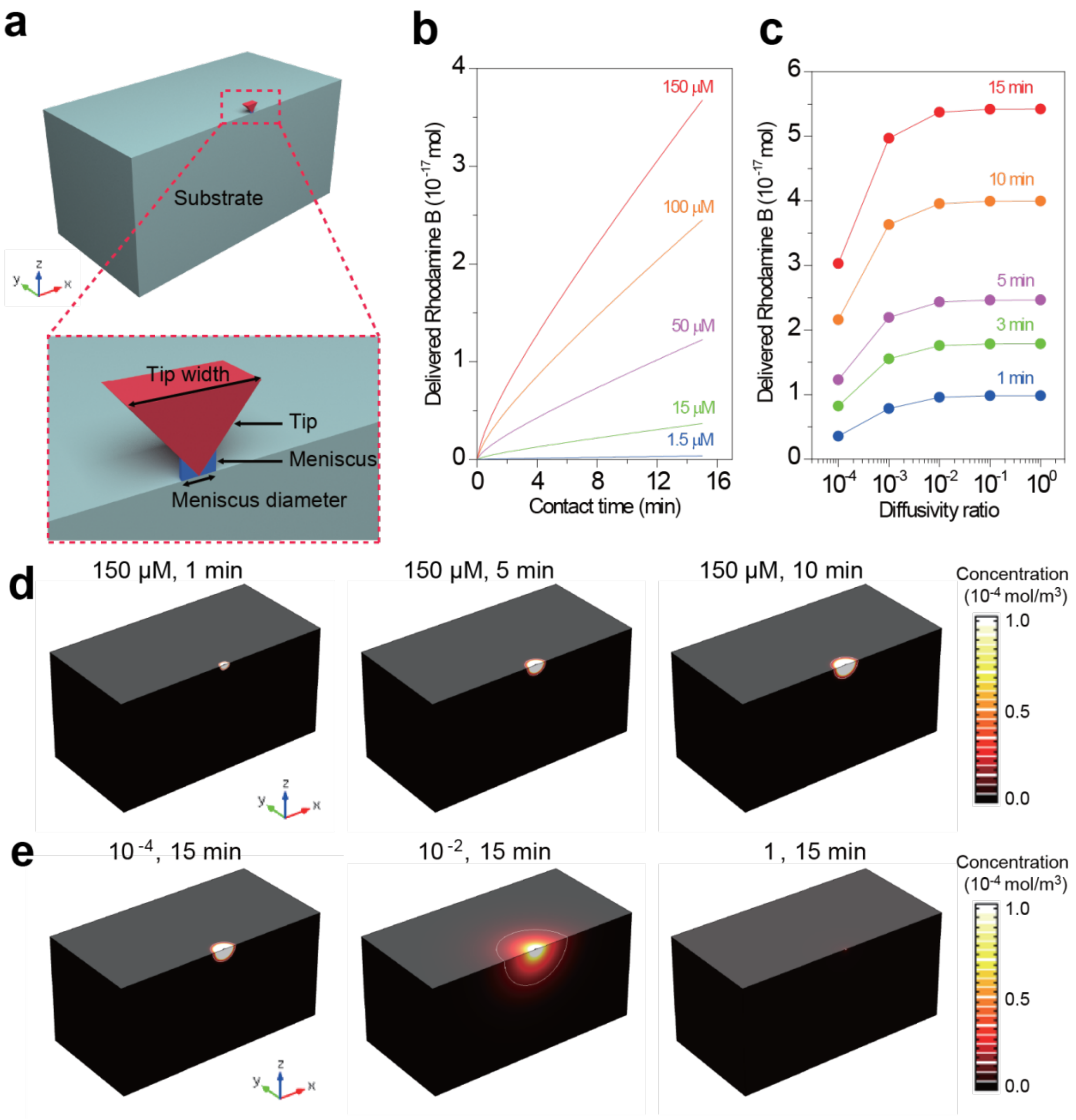

Supplementary Figure 45 | Quantitative analysis of Rhodamine B delivery. a, Cross-sectional view of simulation domain including the tip, the substrate, and the water meniscus. Exterior shape of the meniscus is assumed to be a cylinder with a diameter that is 20% of the tip width at the base. **b,** Calculated Rhodamine B delivery to the PEG-DA MW 700 substrate as a function of contact time with initial Rhodamine B concentrations of 1.5, 15, 50, 100, and 150 μM, respectively. **c,** Calculated Rhodamine B delivery to the substrate as a function of the diffusivity ratio ($D_{substrate}/D_{water}$) at 1, 3, 5, 10, and 15 min, respectively, with the initial Rhodamine B concentration of 150 μM. **d,** Concentration distribution during Rhodamine B delivery with different contact times (1, 5, and 10 min). **e,** Concentration distribution at 15 min after the initial contact as a function of the diffusivity ratio. Iso-concentration contours (white solid lines) are plotted at the concentration of 10^{-5} mol/m^3. Initial Rhodamine B concentrations in the tip are 150 μM for all simulations.

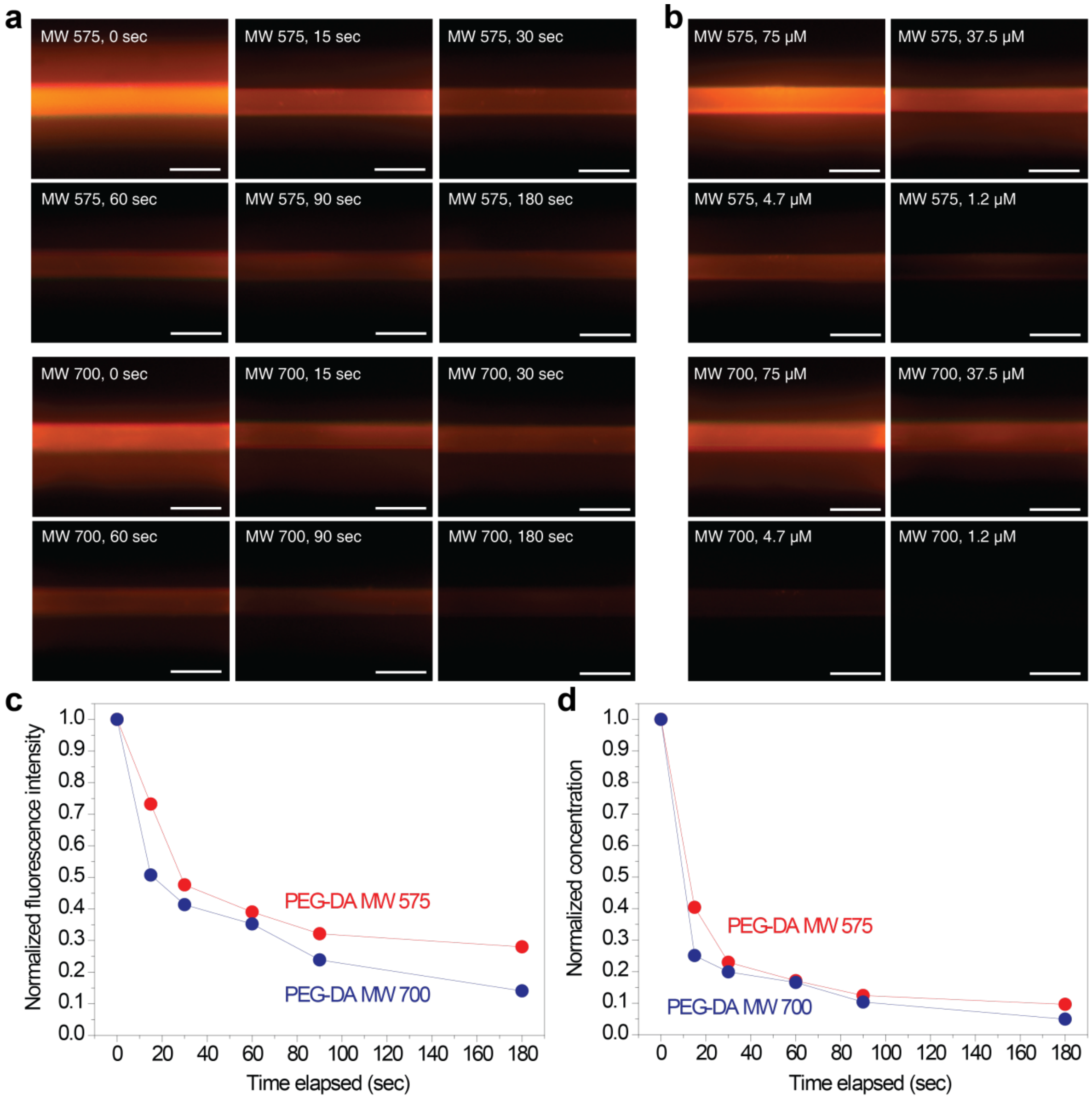

Supplementary Figure 46 | Measurement of Rhodamine B concentration and fluorescence intensity from Rhodamine B-loaded PEG-DA hydrogel cantilevers. a, Time-lapse fluorescence images of Rhodamine B loaded PEG-DA MW 575 and 700 cantilevers (length, width, and thickness: 300, 50, and 10 µm) in water. After each cantilever was immersed in Rhodamine B dissolved in water (150 µM) for 10 min to ensure saturated loading, it was taken out and immediately immersed in fresh DI water. **b,** Fluorescence images of PEG-DA cantilevers (MW 575 and 700) loaded with various Rhodamine B concentrations (75, 37.5, 4.7, and 1.2 µM) at saturation right before dipping in water which are used for intensity-concentration calibration. The exposure time was set to be 500 ms for all images. All images taken from the side show thicknesses of cantilevers. All scale bars are 20 µm. **c-d,** Normalized fluorescence intensity (c) and normalized concentration (d) as a function of dwell time in water. Diffusivities of Rhodamine B in PEG-DA MW 575 and 700 extracted from experimental results were 4.4×10^{-14} m^2 s^{-1} and 7.0×10^{-14} m^2 s^{-1}, respectively.

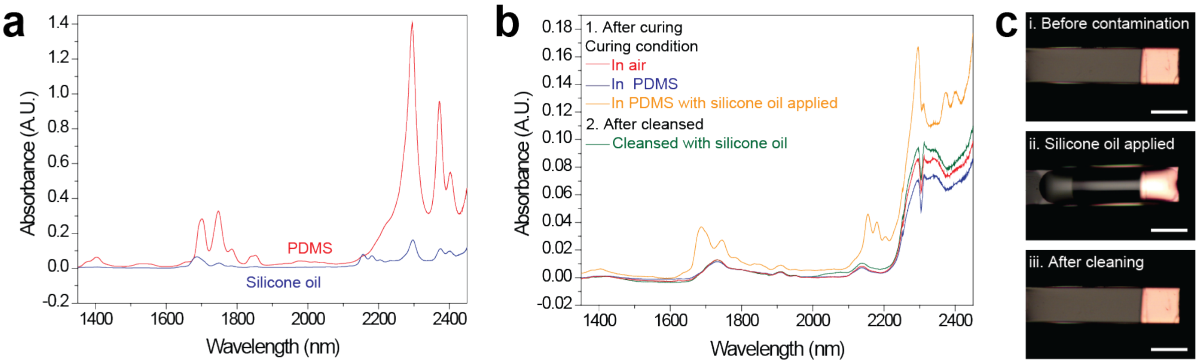

Supplementary Figure 47 | Near infrared (NIR) spectra of fabricated hydrogel probes without or with deliberate contamination and cleaning. a, NIR spectra of PDMS and silicone oil. PDMS could be the source of the suspected contaminant, silicone oil, during hydrogel probe fabrication by replica molding and storage in Gel-Pak. **b,** NIR spectra of PEG-DA MW 250 hydrogel probe cured in the absence of PDMS (red), cured in contact with PDMS (royal blue), cured in contact with PDMS and deliberately contaminated by applying a drop of silicone oil (orange), and after the applied silicone oil was cleaned with acetone (green). **c,** Optical micrographs of hydrogel nano-probes (i) before and (ii) after the contaminant, silicone oil, was applied, and then (iii) cleaned. All scale bars are 50 µm.

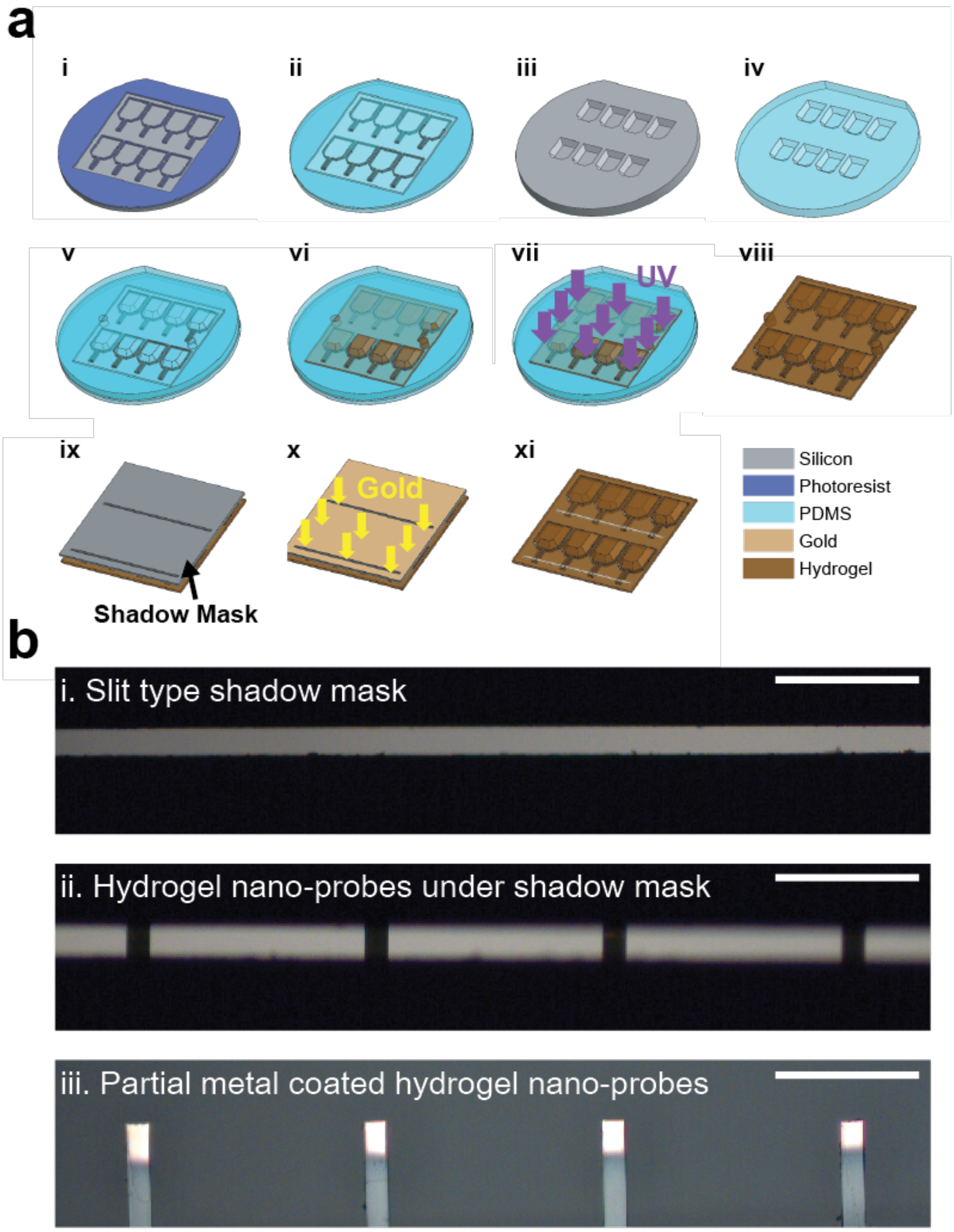

Supplementary Figure 48 | Concept and process of wafer-level batch fabrication of hydrogel nano-probes. **a,** Fabrication processes – i: Negative pyramid structures (silicon etched by KOH) aligned with negative cantilever structures (patterned SU8 photoresist), ii: Tip/cantilever mold (PDMS), iii: Handle mold master (silicon etched by KOH), iv: Handle mold (PDMS), v: Align the PDMS tip/cantilever mold with the PDMS handle mold, vi: Inject the hydrogel pre-polymer, vii: Wafer-scale UV exposure, viii: Batch fabricated hydrogel nano-probes, ix: Align hydrogel nano-probes with a shadow mask for partial metal deposition, x: Metal deposition, xi: Wafer-level batch fabricated hydrogel nano-probes with partial metal coating. **b,** Slit type shadow mask (i), hydrogel nano-probes aligned with the shadow mask (ii), and partial metal coated hydrogel nano-probes (iii). All scale bars are 300 µm.

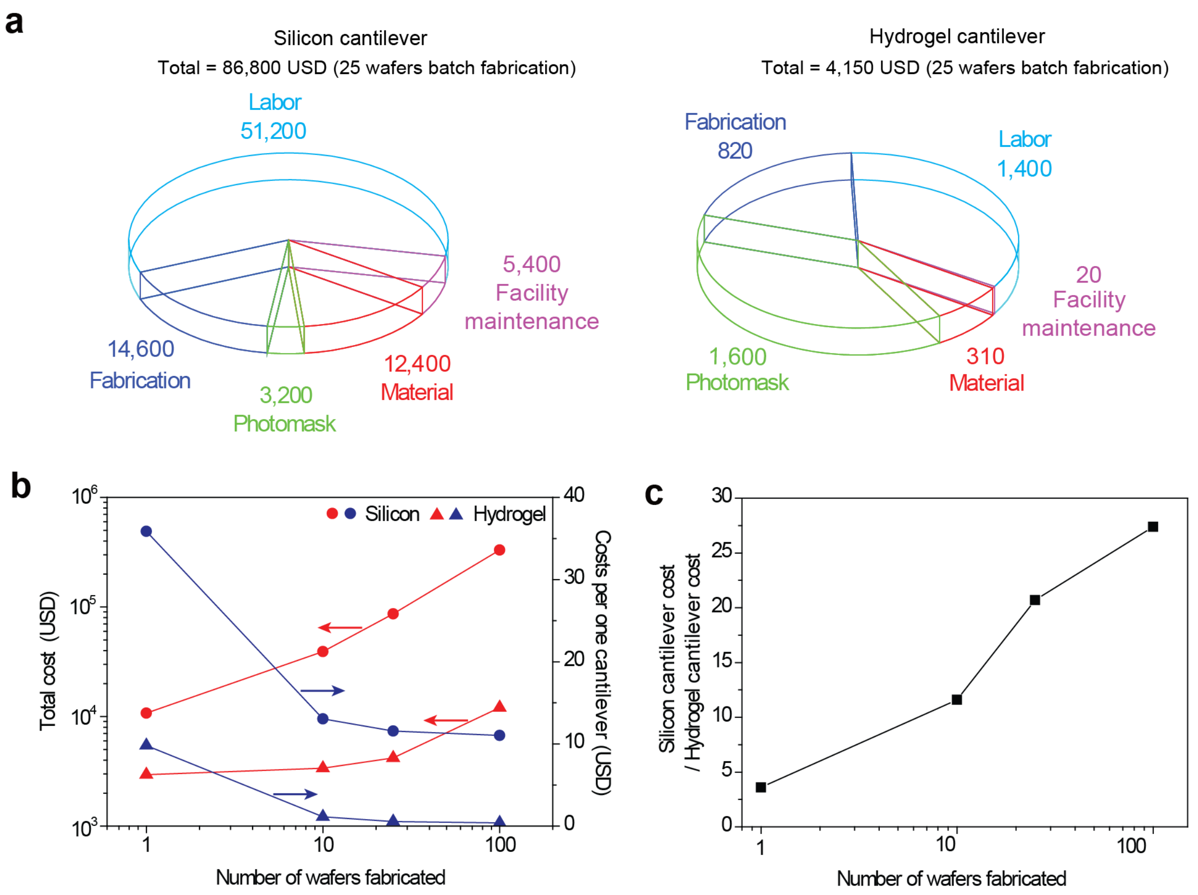

Supplementary Figure 49 | Cost analysis. a, Itemized expenses for fabricating silicon and hydrogel AFM probes (100 wafers each). Fabrication costs include estimated fees for using microfabrication facilities and material costs include wafers, photoresists, developers, gold, and etchant. Facility maintenance costs are deduced by dividing the total costs of utilities, salaries, and supplies with the average number of users in the facilities. **b,** Total cost (left y-axis) and cost per single probe (right y-axis) in US dollars for both silicon and hydrogel AFM probes as a function of the number of wafers fabricated. **c,** Ratio of fabrication cost for silicon probes to that for hydrogel probes as a function of the number of wafers fabricated.

Supplementary Tables

Supplementary Table 1 | Calibration methods for the spring constant of hydrogel nano-probes.

	Thermal method	Reference cantilever method	Stylus profilometer method
Applicable range	0.005 ~ 100 N m^{-1} (ref. 1)	0.02 ~ 25 N m^{-1} (ref. 2)	> 100 N m^{-1}
Nano-probe number in Fig. 3h (Reference cantilever used)	#5	#2 (OCTO500D),<br>#3 (OCTO1000D),<br>#4 (OCTO500S)	#1
Advantage	- User friendly<br>- Wide applicable range	No metal coating is required	- No metal coating is required<br>- Applicable to very stiff cantilevers
Disadvantage	Metal coating is required.	A reference cantilever with a known spring constant is required.	Precise cantilever positioning is required.
Factors determining the applicable range	- Resolution limit of the optical detector<br>- Mechanical stability of the hydrogel cantilever	- Available range of reference cantilever spring constants<br>- Stick-slip motion<br>- Uncertain contact point	Resolution of the stylus profilometer

Supplementary Table 2 | Calculated Tabor coefficients and ratio of adhesion force to maximum force (F_{ad}/F_{max}) for *F-d* measurements in air.

Substrate	Tabor coefficient	F_{ad}/F_{max}
PEG-DA MW 575	~0.19	~ 0.040
PEG-DA MW 700	~0.22	~ 0.045

Supplementary Table 3 | Summary of vertical and lateral tip deformations for *F-d* measurements and tip-sample deconvolution experiments.

Condition	Sample	Tip radius (nm)	Contact radius (nm)	Maximum vertical tip deformation (nm)	Maximum lateral tip deformation (nm)
Liquid (*E*: 410 MPa, 0.4 nN)	Polyacrylamide (0.15%)	150	130.7	0.005	0.0014
Liquid (*E*: 410 MPa, 0.5 nN)	Polyacrylamide (0.30%)	150	146.6	0.01	0.0018
Liquid (*E*: 410 MPa, 3 nN)	PDMS 20:1	150	61.3	0.07	0.022
Air (*E*: 1.46 GPa, 210 nN)	PEG-DA MW 575	150	79.9	1.13	0.33
Air (*E*: 1.46 GPa, 210 nN)	PEG-DA MW 700	150	93.1	0.97	0.28
Air (*E*: 1.46 GPa, 300 nN)	Silicon	30	15.9	8.4	2.38

** Poisson's ratios for PEG-DA hydrogel, polyacrylamide, and silicon samples are 0.4, 0.5 (ref. 3) , and 0.22 (ref. 4), respectively.

Supplementary Table 4 | Elastic modulus comparison between experimentally measured and reference values.

	Measurements		Literature References
	Stylus profilometer	AFM (Force-displacement)	
PEG-DA MW 575	37 MPa	40.4 MPa	-**
PEG-DA MW 700	24.8 MPa	24.7 MPa	-**
PDMS 20:1	-*	1070 kPa	950~1100 kPa (ref. 5)
Polyacrylamide (Bis-acrylamide 0.30%)	-*	38.5 kPa	34.9 kPa (ref. 6)
Polyacrylamide (Bis-acrylamide 0.15%)	-*	16.8 kPa	16.7 kPa (ref. 6)

*: The stylus profilometer method is not available for these samples.
**: Reference values were not found for the specific combination of hydrogel pre-polymer solution and PI, PI concentration, and UV dose used in this study.

Supplementary Table 5 | Material compositions of functional hydrogel nano-probes.

	Rhodamine B	CdTe quantum dots	Co nanoparticles	Au nanoparticles
Molecular weight of PEG-DA	575 g mol^{-1}	700 g mol^{-1}	250 g mol^{-1}	700 g mol^{-1}
Material characteristic length	1.6 nm (molecular size)	4.0 nm (diameter)	30 nm (diameter)	50 nm (diameter)
Content of functional particles or dyes	4 µM	~0.03% in weight	~20% in weight	~0.02% in weight
Content of water	-	~40% in weight	-	~40% in weight
UV dose	590 mJ cm^{-2}	120 mJ cm^{-2}	120 mJ cm^{-2}	590 mJ cm^{-2}
Observation mode*	Fluorescence	Fluorescence	Bright field	Dark field

*: used for making merged images shown in Fig. 8a,b.

Supplementary Discussion

Tip sharpness improvement via lateral and vertical tip mold compression

Supplementary Fig. 5 summarizes the fabrication results of the lateral tip mold compression approach. In general, the aspect ratio increases and the tip gets sharper as the applied bi-axial compressive strain increases. When the bi-axial strain surpasses 15%, the local aspect ratio around the tip apex region significantly increases and eventually nanowire-attached hydrogel nano-probes can be fabricated (Supplementary Fig. 5c). Such nanowire-attached hydrogel probes would be useful for imaging deep trenches.

The lateral tip mold compression generally increases the tip aspect ratio and decreases the tip radius by applying bi-axial compression with two independent actuators. The independent axis control would be advantageous when the PDMS tip mold is slightly asymmetric. This asymmetry results from the deviation of an exact square or circle of the oxide etch mask for KOH etching of the silicon wafer which becomes the master mold for the PDMS replica molding. If the tip mold is symmetric, then vertical tip mold compression that requires one degree-of-freedom deformation can be applied to improve the tip sharpness (Supplementary Fig. 6). In the vertical compression scheme, a tipless hydrogel cantilever is pushed against the pre-polymer solution-filled pyramid mold. This induces localized vertical compression of the tip mold but squeezes the tip apex region of the tip mold. As a result, the replicated hydrogel tip becomes sharper while the aspect ratio is compromised.

Metal coating on hydrogel cantilevers for optical readouts

A metal coating was added to the top side (side opposite to the tip integration side) of the hydrogel cantilever in order to permit light reflection for AFM operation. In our experiments, a 120 nm thick metal coating was selectively deposited on the top side of hydrogel cantilevers by using a PDMS shadow mask (Supplementary Fig. 7a, b). With the PDMS shadow mask, the coverage of the metal coating on each cantilever was controlled to be 10, 50 or 100% from the free end in order to investigate its swelling-induced bending characteristics in liquids (Supplementary Fig. 8). Metal-deposited hydrogel cantilevers were fully immersed in water and PBS solution (PBS, Sigma Aldrich) and their bending behaviors were recorded using an inverted optical microscope (IX81, Olympus). The hydrogel cantilever with full metal coverage bent towards the metal-deposited side while others (10 and 50% coverage) remained flat. The metal coating only covered part of the top side of the cantilever in order to promote equal water absorption across the entire hydrogel material, thereby enabling the cantilever to remain flat (Supplementary Fig. 8). If the size or the position of the metal reflector is properly adjusted (Supplementary Fig. 9a, b), then the tip apex region is visually accessible due to the transparency of the hydrogel and aids tip registration to specific locations during experiments.

Parylene coating for hydrogel cantilever

We are also investigating additional fabrication steps in order to completely prevent water absorption into the hydrogel cantilever. Before tip integration, a 1 μm thick layer of parylene can be conformally deposited around the hydrogel cantilever with a chemical vapor deposition coater (DACS-LAB, Femto Science). In this case, only the hydrogel tip would respond to swelling, which may be attractive for materials delivery applications. As shown in

Supplementary Fig. 13, the hydrogel cantilever can be coated with parylene and it behaves similarly in air and water.

Imaging performances of hydrogel nano-probes

Imaging performances of hydrogel nano-probe were investigated for many aspects (i.e. soft biological sample imaging, high-resolution imaging, long-term imaging). First, Calibration gratings were imaged in air and water with both contact and noncontact modes. Spring constants and resonance spectra of the hydrogel probes were measured in air and water by using commercial AFM instruments (NX10 and NX-Bio, Park Systems) prior to contact and noncontact mode AFM imaging. The imaging results were then compared with those obtained using commercial silicon probes. Hydrogel probes provide topographical imaging with quality that is at least similar to that of commercial silicon probes (Supplementary Fig. 21).

To investigate imaging performances for soft biological samples, several fixed cells including MCF-7, MRC-5, and HeLa cells were prepared and imaged in air and PBS solution with both contact and noncontact modes (Supplementary Figs. 26 and 27). Unless otherwise noted, a hydrogel AFM nano-probe with a radius of curvature of approximately 25-30 nm was used for all measurements. For contact mode imaging, a contact force range of 0.5 to 300 nN and a scan rate range of 0.5 to 10 Hz were used. For noncontact mode imaging, the vibration amplitude setpoint was kept at 80% of the free vibration amplitude at a given drive condition and a scan rate between 0.25 and 5 Hz was used. For detailed comparison with the fabricated hydrogel AFM probes, several commercial silicon AFM probes (CONTR, Nanosensors; PPP-NCHR, Nanosensors; Biolever mini, Olympus; PPP-FMR, Nanosensors) were used at operating conditions similar to those for hydrogel probes. Feedback control parameters, however, were optimized separately until the best imaging qualities were obtained for both commercial silicon and fabricated hydrogel probes. Height images were plotted by using the Gwyddion 2.39 and XEI 4.1.0 programs.

To evaluate the high resolution imaging capability of the hydrogel nano-probes, a comparative study was performed by imaging identical regions of dried HeLa cells with silicon (NSC36-B, Mikromasch) and hydrogel probes (Supplementary Fig. 28). The spring constant of the hydrogel nano-probe was matched with that of the silicon probe for fair comparison (2 N m^{-1}). The imaging area was sequentially reduced (1st frame: 35 μm × 35 μm, 2nd frame: 6 μm × 6 μm, and 3rd frame: 600 nm × 600 nm). This result shows that the hydrogel probe can deliver high-resolution cell imaging which is equivalent to that obtained with the silicon probe.

Lastly, to investigate the long-term imaging stability of hydrogel nano-probes (especially in liquid conditions), human fibroblast cells were imaged in PBS solution with contact mode for 4 hrs (Supplementary Fig. 31). Imaging quality was maintained without showing any evidence of degradation or contamination.

High speed imaging performances for soft biological samples

Though high speed performance of hydrogel nano-probes was investigated as shown in Fig. 6, fixed cells (MRC-5) were imaged in noncontact mode at various scan rates to apply the outstanding performance of hydrogel nano-probes (length, width, and thickness: 220, 50, and 20 μm) for noncontact mode high speed imaging to biological samples. The scan size was set

to be 40 μm × 40 μm with the pixel resolution of ~40 nm/pixel (Supplementary Fig. 29). For benchmarking, a commercial silicon probe (Biolever mini, Olympus) was also employed. Feedback parameters were optimized for each cantilever at 1 Hz and maintained at higher scan rates. Again, the hydrogel probe outperformed the silicon probe. Only the hydrogel probe maintained the image quality up to 25 Hz. This is attributed to the higher f_0/Q ratio of the hydrogel probe compared to that of the silicon probe (1.21 vs. 0.43).

Suspected fracture damage of the silicon probes

During the noncontact mode high speed imaging test with silicon and hydrogel probes, we have observed significant image degradation after 50 Hz imaging only with the silicon probe (Fig. 5e). We suspected fracture damage in the vicinity of the tip apex due to its sudden occurrence. To determine when and where the silicon probe got damaged, we have examined sequential height images between the transition from 50 to 1 Hz. The z-servo gain (G) was increased from 1.5 to 2 after the noncontact mode image at 50 Hz (i in Supplementary Fig. 24a) was obtained to handle the imaged distortion. However, the image became worse and showed high-speed distortion as well as evidence for tip degradation (ii in Supplementary Fig. 24a). Then, the scan rate and/-or z-servo gain were decreased down to 1 Hz and/-or 1.5, respectively. However, the periodic hexagonal structure of the AAO could no longer be captured (iii, iv in Supplementary Fig. 24a). After the noncontact mode imaging at 1 Hz and with the z-servo gain of 1.5, the silicon probe tip was examined under with the scanning electron microscope (CX-200TM, COXEM). The SEM image taken after the high speed noncontact mode imaging experiment shows a clear difference from its initial SEM image (Supplementary Fig. 24b). The observed tip degradation with the silicon probe is thought to be fracture damage considering its sudden occurrence.

Stability of hydrogel cantilevers with partial metal coating

For comparison of the stability performance of hydrogel cantilevers with that of silicon cantilevers, two kinds of hydrogel-silicon cantilever pairs were chosen. Either hydrogel or silicon cantilevers were mounted on the NX10 AFM in an acoustic enclosure to measure time-dependent static deflection and resonance frequency while the temperature and relative humidity (RH) near the cantilever were monitored by a USB-type temperature and humidity sensor module (TSP01, Thorlabs). The sensor module was configured as close as possible to the cantilever (~5 mm apart) inside the acoustic enclosure. The measured temperature was ~30 °C and the relative humidity was 20 and 100% RH in air and liquid, respectively. The first cantilever pairs include three hydrogel cantilevers (type #1; length, width, and thickness: 240-250, 50, and 10 μm; k_{air} = 1.2-1.3 N m^{-1}) and three silicon cantilevers (NSC36-B, Mikromasch; k_{air} = 1.1-1.5 N m^{-1}) for deflection stability comparison with similar spring constants. Supplementary Fig. 20a shows static deflection of a hydrogel cantilever (type #1) in air, PBS solution, and water. Standard deviations of the static deflection of three hydrogel (type #1) cantilevers were measured to be 4.95, 2.76, and 2.56 nm in air, PBS solution, and water, respectively. Similar measurements were done with three silicon cantilevers (NSC36-B, Mikromasch) and results were compared directly in Supplementary Fig. 20c. For static operations (i.e., contact mode imaging and force-displacement measurements), the hydrogel (type #1) cantilevers are generally less stable than the silicon cantilevers (NSC36-B, Mikromasch) in air while they become more stable than NSC36-B in liquid. In addition, the hydrogel (type #1) cantilevers are the most stable in water. For fair comparison, the static deflection measured was converted to the bending angle, θ =tan^{-1} (δ/l), and results were

shown in Fig. 4c. The hydrogel (type #1) cantilevers are generally more stable than the silicon cantilevers (NSC36-B, Mikromasch) and the most stable in water.

The second cantilever pairs include three hydrogel cantilevers (type #2; length, width, and thickness: 200-220, 50, and 20 μm; f_{air} = 80-92 kHz) and three silicon cantilevers (BL-AC40TS, Olympus; f_{air} = 112-124 kHz) for resonance stability comparison with similar resonance frequencies. Supplementary Fig. 20b shows resonance frequencies of a hydrogel cantilever (type #2) in air, PBS solution, and water. Standard deviations of the resonance frequencies of three hydrogel (type #2) cantilevers were measured to be 16.1, 8.2, and 7.2 Hz in air, PBS solution, and water, respectively. Similar measurements were conducted with three silicon cantilevers (BL-AC40TS, Olympus) and results were compared directly in Supplementary Fig. 20d and in Fig. 4d after normalized by average values. For both absolute and normalized frequencies, the hydrogel (type #2) cantilevers are more stable than the silicon cantilever (BL-AC40TS, Olympus) in air, PBS solution, and water. In addition, the hydrogel (type #2) cantilevers are the most stable in water which is similar to the result observed in static stability tests.

Cellular imaging performance and stability under temperature modulation

Considering the temporal and spatial variations in environmental conditions such as pH or temperature that can possibly be encountered during cell imaging, the imaging performance and stability of hydrogel probes were investigated with MRC-5 fibroblast cells in PBS solution under temperature modulation between 34.5 and 40.4 °C by using a calibrated ceramic heater (Ultramic, Watlow). The range of temperature modulation was chosen to be wide enough to cover any dynamic change in typical cellular environments. For comparative reference, the same cellular region was imaged under the buffer temperature maintained at ~36 °C (Supplementary Fig. 28a, b). Even with the buffer temperature modulation, the imaging performance and stability were maintained without showing any noticeable difference.

Estimation of tip deformation

Since significant deformation of the tip would deteriorate the accuracy of *F-d* measurements, both the vertical and lateral tip deformations of hydrogel nano-probes were estimated. For the estimation of the vertical tip deformation, the contact between an elastic sphere and an elastic half space (i.e., infinitely flat plane) was assumed. As the tip contacts the sample, pressure is applied to a circular region with a contact radius defined as:

$$a = \sqrt{R\delta} \qquad (1)$$

where a, R, and δ represent the contact radius, tip radius, and total deformation, respectively. Assuming a frictionless contact, the pressure between the tip and sample following the Hertzian theory is given by $p(r) = \frac{p_0(a^2 - r^2)^{1/2}}{a}$, with the origin at the center of the contact, where r and p_0 represent the distance from the center and the maximum pressure, respectively[7]. With the pressure given, the normal displacement of the tip is expressed as

$$\overline{u_t(r)} = \frac{1-\nu_t^2}{E_t}\frac{\pi p_0}{4a}\left(2a^2 - r^2\right),\ r<<a \qquad (2)$$

where $\overline{u_t}$, ν_t, and E_t represent the normal displacement, Poisson's ratio, and elastic modulus of the tip, respectively.

The maximum normal displacement (deformation) of the tip and sample occurs at the center point (r = 0).

$$\overline{u_t(0)} = \frac{1-\nu_t^2}{E_t}\frac{\pi p_0}{2}a \qquad (3)$$

The total load compressing the sample, P, is related with the pressure by

$$P = \frac{2}{3}p_0 \pi a^2 \qquad (4)$$

Substituting p_0 with P using equation (4), the maximum normal displacement of the tip can be expressed as:

$$\overline{u_t(0)} = \frac{3P(1-\nu_t^2)}{4E_t a} \qquad (5)$$

As such, the normal displacement of the sample as a function of the distance r from the center can be expressed as:

$$\overline{u_s(r)} = \frac{1-\nu_s^2}{E_s}\frac{\pi p_0}{4a}\left(2a^2 - r^2\right),\ r<<a \qquad (6)$$

$$\overline{u_s(0)} = \frac{1-\nu_s^2}{E_s}\frac{\pi p_0}{2}a \qquad (7)$$

where $\overline{u_s}$, ν_s, and E_s represent the normal displacement, Poisson's ratio, and elastic modulus of the sample, respectively. The vertical tip deformation at the tip apex was calculated for certain cases with the corresponding maximum force applied (Supplementary Table 3). It was shown that the vertical tip deformation at the tip apex is almost negligible compared to the tip radius for *F-d* experiments. The vertical tip deformation at the tip apex during the tip-sample deconvolution experiment with 300 nN force applied was ~8.4 nm which is appreciably higher than the other cases. However, the high contact force of 300 nN on a hard silicon substrate is not a typical imaging or *F-d* experiment condition.

Due to the tilt of the probe, the lateral tip deformation during *F-d* measurement was also investigated. The geometry of the tip contacting the sample surface with a tilt angle was simplified to be a tapered rod with a squared cross-sectional area (Supplementary Fig. 18) where a point force is applied at the center of tip apex. The width of the square at tip apex was assigned to have the same cross-sectional area as the contact area of circular region derived from the calculated contact radius. For this simplified model, the exact solution satisfying the equilibrium equation can be obtained. Since it has a non-uniform cross sectional area, the following differential equation[8] can be considered :

$$\frac{d^2\left(EI\frac{d^2y}{dx^2}\right)}{dx^2}=q \qquad (8)$$

where E, I, and q are the elastic modulus, moment of inertia, and distributed load, respectively. For this case, q=0. The moment of inertia for rectangular cross section is a function of x as follows:

$$I=\frac{\left(\frac{w_2-w_1}{L}x+w_1\right)^4}{12} \qquad (9)$$

where w_1 and w_2 are widths of the tip at x=0 and x=L, respectively, and L is the tip height. The differential equation (8) can be solved with the following four boundary conditions:

$$y\big|_{x=0}=0 \qquad (10)$$

$$\left.\frac{dy}{dx}\right|_{x=0}=0 \qquad (11)$$

$$\left.\frac{d\left(EI\left(\frac{d^2y}{dx^2}\right)\right)}{dx}\right|_{x=L}=F\sin(\alpha) \qquad (12)$$

$$\left.EI\frac{d^2y}{dx^2}\right|_{x=L}=0 \qquad (13)$$

The following exact solution for lateral tip deformation (y) is obtained:

$$y=-\frac{2L^2F\sin(\alpha)(3Lw_1-3w_1x+2w_2x)x^2}{Ew_1(Lw_1-w_1x+w_2x)^2} \qquad (14)$$

Supplementary Table 3 summarizes maximum vertical and lateral tip deformations of hydrogel nano-probes used in *F-d* measurements and tip-sample deconvolution experiments. Since the vertical tip deformation was calculated at the center of contact area and the lateral tip deformation was calculated by assuming the point lateral force being applied at the contact center, tabulated values represent maximum possible deformations (i.e. actual deformations are expected to be smaller than those values tabulated). Specifically, lateral tip deformations for *F-d* measurements are negligibly small compared to tip radii of hydrogel nano-probes. Therefore, even if there is a non-zero lateral tip deformation, the Hertz contact model can be applied as long as the spherical indenter region but the sidewall of the tip contacts with the sample. Of note, the tip-sample deconvolution experiments were done with excessively high contact force and a hard silicon substrate in order to indirectly quantify the tip deformation during high contact force imaging experiments (Fig. 6c,d) rather than *F-d* measurements.

Tip radius estimation from tip-sample deconvolution experiments

Even though Supplementary Fig. 25 already shows the AFM image quality of hydrogel probes comparable to that of commercial silicon probes at a moderate pixel resolution (60~140 nm pixel^{-1}), it would be necessary to quantify tip deformation of relatively soft and compliant hydrogel nano-probes induced by contact force or swelling during imaging at a higher pixel resolution. To this end, a hydrogel nano-probe (length, width, and thickness: 220, 50, and 20 μm) of its initial tip radius of sub 30 nm was used to image cylindrical hole regions of the calibration grating (HS-100MG, Nanosensors). An identical location of the grating was sequentially imaged using the hydrogel nano-probe in air at the force setpoint of 0.3, 3, 30, and 300 nN (Supplementary Fig. 25a). The scan size was confined to 5 μm × 5 μm which corresponds to the pixel resolution of 10 nm pixel^{-1}. After imaging at 300 nN, the force setpoint was adjusted to be 0.3 nN and the same region was imaged in water. The small cumulative scan distance of ~25.6 mm prevents wear-induced tip degradation. To estimate the tip radius[9], all acquired height images were processed by using the tip-sample deconvolution to obtain the 3D tip shape using the Gwyddion 2.39 program (Supplementary Fig. 25b). Deconvoluted images shown in Supplementary Fig. 25b were processed to have pixels of 128 × 128 (780 nm × 780 nm), and sparse discrete data points along the line A-A' (300 nm length) were interpolated by the B-spine (polynomial degree of 3) to have 2000 points with 0.15 nm step. This interpolation is necessary for the subsequent circular fitting to obtain the tip radius. Supplementary Fig. 25c shows the tip shape (line scan along A-A' in Supplementary Fig. 25c) for each imaging condition. The estimated tip radii were 27.4, 27.4, 27.9, and 29.5 nm for 0.3, 3, 30, and 300 nN contact forces, respectively, all of which are similar to the initial tip radius. 1000-times contact force increase from 0.3 to 300 nN resulted in only a 2.1 nm increase in tip radius. At our regular setpoint values (≤3 nN), the tip deformation induced by physical contact is not significant at all. The tip radius estimated from the height image at 0.03 nN in water was 27.6 nm which is also similar to the initial tip radius. This small change also agrees with the small swelling (~2% for PEG-DA MW 250 cured with 590 mJ cm^{-2}) previously shown in Supplementary Fig. 14. In summary, we can draw two conclusions. First, the tip deformation at our typical setpoint is negligible. Second, the tip swell is not significant at all to affect the imaging quality for our main targets (soft matter including biological samples).

Long-term durability of the hydrogel-metal interface

To check the durability of the hydrogel-metal interfacial adhesion contact during excessively long-term exposure to a liquid environment, the metal-coated hydrogel nano-probe was incubated in water for 50 hrs. As shown in Supplementary Fig. 32a, no visual evidence of degradation was observed. After a 50 hr long incubation in water and subsequent drying, the imaging tip was attached to the hydrogel probe which was then successfully used to image HeLa cells in PBS solution (Supplementary Fig. 32b). This experiment proved that the functionality of the partial metal coating on hydrogel probes could be maintained during experiments that last longer than two consecutive days.

Application of soft hydrogel nano-probes for friction measurements

Since soft hydrogel nano-probes which exhibit low bending stiffness also have low torsional stiffness, they would be potentially useful for friction measurements in the lateral force microscopy (LFM) mode. To experimentally confirm the promise of this approach, two

hydrogel nano-probes were prepared with spring constants on the range of 1.4 (PEG-DA MW 250, length, width, and thickness: 230, 50, and 10 μm), and 0.02 N m^{-1} (PEG-DA MW 575, length, width, and thickness: 280, 50, and 10 μm), respectively. Supplementary Fig. 22a, b show deflection sensitivity and thermomechanical noise spectrum of the softer hydrogel probe. These two hydrogel nano-probes were employed for LFM imaging on graphitic layers adhered to a silicon substrate (Supplementary Fig. 23a, b). As expected, enhanced LFM voltage signals showing a higher friction contrast between the graphitic layers and the underlying silicon were observed with the softer hydrogel probe (Supplementary Fig. 23c).

Computational method to simulate Rhodamine B delivery

Numerical simulations were performed by using commercial finite element analysis software (COMSOL Multiphysics 4.4, COMSOL Inc.). In order to quantify the content of Rhodamine B delivered, the local concentration of Rhodamine B was investigated by solving the diffusion from the tip to the substrate through a water meniscus. The substrate was thought to be sufficiently large (length, width, and thickness: 500, 500, and 250 μm) with an open boundary condition with zero exterior concentration. The exterior shape of the meniscus was assumed to be a cylinder for which the diameter was 20% (5 μm) of the tip base width and height of 3.2 μm (Supplementary Fig. 45a). Diffusivity of Rhodamine B in water was set to be 4×10^{-10} m^2 s^{-1} (ref. 9) and those for PEG-DA MW 575 and 700 substrates were experimentally measured to be 4.4×10^{-14} m^2 s^{-1} and 7.0×10^{-14} m^2 s^{-1}, respectively (Supplementary Fig. 45). Initially, Rhodamine B was present only in the tip. After 15 min transient simulations, the local Rhodamine B concentration in the substrate was integrated to calculate the overall content of Rhodamine B delivered. Quantitative analyses of Rhodamine B delivery for different initial concentrations in the tip (1.5, 15, 50, 100 and 150 μM) and diffusivity ratios ($D_{substrate}/D_{water}$) were carried out (Supplementary Fig. 45b,c). Rhodamine B delivery is linearly proportional to the initial concentration in the tip. At 15 min after the initial contact, 3.7×10^{-19}, 3.7×10^{-18} and 3.7×10^{-17} mol of Rhodamine B was delivered with initial concentrations of 1.5, 15 and 150 μM, respectively. Concentration distributions in the substrate with different contact times show that the maximum concentration in the substrate decreases and the iso-concentration surface expands as the contact time increases (Supplementary Fig. 45d). Rhodamine B delivery becomes saturated as the diffusivity ratio approaches to unity. At 15 min after the initial contact, 3.03×10^{-17}, 5.37×10^{-17} and 5.42×10^{-17} mol of Rhodamine B was delivered and the maximum concentrations were 6.0×10^{-2}, 1.1×10^{-3} and 1.3×10^{-5} mol m^{-3} with diffusivity ratios of 10^{-4}, 10^{-2} and 1, respectively. As the diffusivity ratio increases, Rhodamine B delivery increases and it spreads over a larger region (Supplementary Fig. 45e).

Contamination and cleaning of hydrogel nano-probes

Replica molding and UV curing within the PDMS mold assembly may inherently contaminate as-fabricated hydrogel probes because PDMS can leave residual silicone oil[11] from the fabrication process. In addition, fabricated hydrogel probes stored in Gel-Pak, a typical container for regular AFM probes, may also be subject to contamination with silicone oil released and slowly evaporated from the sticky gel layer. While the second case can be completely prevented by storing hydrogel probes in a gel-free container, the first case is not avoidable. To quantify the amount of such inherent contamination, near infrared spectra (NIR) exhibiting a unique fingerprint for silicone oil were measured with hydrogel probes fabricated and treated differently by using the NIR spectrometer (NIR SCAN, Texas

Instruments) (Supplementary Fig. 47a, b). NIR spectra of both PDMS and silicone oil show peaks at wavelengths around 1680, 1750, 2290, 2370, and 2410 nm. Similar NIR spectra from hydrogel (PEG-DA MW 250) substrates cured with and without physical contact with PDMS may imply that the suspected inherent contamination of silicone oil during replica molding and UV curing is either absent or at least below the detection limit of the NIR spectrometer used. Intentionally, the hydrogel substrate cured in contact with PDMS was contaminated by applying silicone oil drops and its NIR spectrum was measured. Then, the contaminated hydrogel substrate was cleaned by dipping it in acetone and its spectra was measured again. The signature of the silicone oil almost completely disappeared after acetone cleaning. The intentional silicone oil contamination and acetone cleaning procedures repeated with hydrogel nano-probes confirmed that the silicone oil contamination encountered in any circumstance can be simply remedied (Supplementary Fig. 47c).

Batch fabrication and cost analysis

Hydrogel nano-probes can be batch fabricated for mass production. Once wafer-scale tips/cantilevers and handle molds are aligned, the hydrogel pre-polymer solution is introduced into the aligned wafer-scale mold assembly. The channel network is specifically designed to easily inject the hydrogel pre-polymer into an array of individual tip/cantilever-handle mold assemblies with dead-end. Once a wafer-scale UV irradiation from a flood exposure cures the injected pre-polymer, several hundreds of tip-integrated hydrogel nano-probes are fabricated simultaneously. Supplementary Fig. 48a shows the concept, fabrication processes, metal coating result of batch fabrication of hydrogel nano-probes. A slit type shadow mask was fabricated to be aligned with hydrogel nano-probe arrays for simultaneous metal coating (Supplementary Fig. 48b).

Lastly, we have done cost analysis for volume production of hydrogel and silicon probes and made a detailed comparison between them (Supplementary Fig. 49). Assuming 100 wafer batch processing, the overall fabrication cost for hydrogel AFM probes would be only ~3.4% of that for silicon AFM probes.

Supplementary Methods

Fabrication methods for tipless hydrogel cantilevers

Tipless hydrogel cantilevers can be fabricated by three different methods, which rely on extended open-end mold and cutting, open-end mold, and dead-end mold, respectively (Supplementary Fig. 1a, b, and c). In common, three methods use two PDMS molds – cantilever and handle molds – aligned where a hydrogel mixed with a photoinitiator (pre-polymer solution) is introduced and then cured by UV irradiation. For each method, the cantilever mold is different while the handle mold is identical. Supplementary Fig. 1 shows detailed fabrication steps of each method. Among the three methods, the most frequently used method is the extended open-end and cutting method (Supplementary Fig. 1a) since it provides the best free-end shape (i in Supplementary Fig. 1d) and offers post control of the cantilever length by cutting with a razor blade. However, it would not be ideal if tipless hydrogel cantilevers with a fixed length are to be made repeatedly due to the resolution of the cutting process. Open-end and dead-end methods were developed as alternatives to address this issue. The fabrication steps are the same as extended open-end and cutting method except that the cantilever mold has a fixed length with open or dead ends. When the pre-polymer solution is simply introduced into the open-end beam mold via capillary forces, the negative Laplace pressure at the end of the open-end mold automatically stops the flow. In experiments, this process took less than 3 s. By contrast, when the pre-polymer solution is introduced into the dead-end mold, the beam mold is slowly filled due to the trapped air within the mold assembly. Eventually, the trapped air escapes through the PDMS which is gas permeable and the cantilever mold becomes completely filled with the pre-polymer solution. In general, this process took ~30 min, which is an appreciably longer time scale than that of the other two methods. In contrast to the extended open-end and cutting method which generally provide sharp right-angled corners, the open-end mold method results in concave corners at the free end which are due to the negative Laplace pressure and the dead-end mold method results in convex corners at the end which come from the SU8 master mold (Supplementary Fig. 1d).

Tip mold fabrication and integration

Tip mold fabrication methods (Supplementary Fig. 2) were explained in detail in Methods. A fabricated tip mold was filled with ~10 pL of the pre-polymer solution and mounted on the UV LED setup (Fig. 2a). A tipless hydrogel cantilever was aligned with the tip mold under a bright field optical microscope (LV100, Nikon) and made contact with either undeformed or deformed pyramidal tip molds (Supplementary Fig. 3a, b). Since the PEG-DA is transparent, the position of the tip attachment and the degree of compression can be monitored. Upon UV exposure (590 mJ cm^{-2}), the pre-polymer solution within the tip mold was cured and the hydrogel tip became firmly attached to the hydrogel cantilever.

Tip radius estimation protocol from SEM images

When hydrogel nano-probes were imaged with a normal scanning electron microscope (CX-200TM, COXEM), the imaging resolution was limited. In addition, hydrogel nano-probes were often imaged under FE-SEM without a metal coating. For example, SEM inspection does not allow a metal coating around the tip apex for successive AFM imaging during the durability test. In such cases, the quality of SEM images is compromised. Therefore, an image processing algorithm and protocol is necessary for tip radius estimation from SEM

images. A custom image processing algorithm was developed by using the Labview Vision Toolkit (National Instruments). Supplementary Fig. 4 shows the image processing sequence. An SEM image of a hydrogel nano-probe tip is 2D FFT low pass filtered to remove high frequency noises noted as white or gray dots in the dark background and smoothed. Then, the scale bar at the bottom right corner is detected and used for the pixel-length calibration. For tip radius estimation, the region of interest around the tip apex is determined and masked. The contrast in the masked region is enhanced by ~15% to amplify the tip-background boundaries with a median filter while suppressing the amplification of the background noise. Finally, the detected amplified tip-background boundaries are fitted to a circle.

Stylus profilometer experiments

The elastic moduli of PEG-DA cantilevers were measured by the stylus profilometer (Dektak XT, Bruker)[1]. First, loading force was calibrated both in air and water using a commercial silicon cantilever (OCTO500D, Micromotive) in order to minimize errors in the measured elastic moduli (Supplementary Fig. 10). Dimensions and fundamental resonance frequency were measured by a scanning electron microscope (FE-SEM 4800, Hitachi) and an atomic force microscope (NX10, Park Systems), respectively, in order to obtain the elastic modulus of the silicon cantilever. Then, the cantilever was scanned along the longitudinal direction by the stylus profilometer at indicated loading forces ranging from 9.8 to 98 μN and static deflection of the cantilever was recorded. Taking into account the dimensions and elastic modulus, the actual loading force (F) was calculated from the observed static deflection by using the Euler-Bernoulli beam equation:

$$k = \frac{F}{\delta} = \frac{Ewt^3}{4l^3} \qquad (15)$$

where w, t, and l are the width, thickness, and length of the cantilever, respectively, E is the elastic modulus, k is the spring constant and δ is the static deflection. Hydrogel cantilevers of defined size (length, width, and thickness: 2000, 350, and 100 μm) were then characterized using a procedure similar to one for the reference silicon cantilever. Both the commercial silicon and fabricated PEG-DA MW 250 cantilevers were scanned at 82 μN and the fabricated PEG-DA MW 575 and 700 cantilevers were scanned at 16 μN under steady-state swelling conditions (Fig. 3a). The dimensions of the hydrogel cantilevers were measured by an upright fluorescent microscope (LV100D with Intensilight C-HGFI, Nikon). 4 μM Rhodamine B dye was encapsulated into PEG-DA hydrogel cantilevers (length, width, and thickness: 2000, 350, and 100 μm). The fluorescence microscope along with a 10a 10× objective lens and CCD camera (ProgRes® C10^{plus}, JENOPTIK) was used to capture fluorescence images for 80 min with 10 min intervals (Supplementary Fig. 12a, b, and c). The exposure time was set to 50 ms for all images. All fluorescence images taken were analyzed by a custom Python code. At a given time, transient linear expansions of each PEG-DA hydrogel sample were obtained from cantilever edges found by the edge detection based on Otsu's thresholding method[12]. Once cantilever edges were found, fluorescent intensities were calculated by averaging the RGB luminance in each pixel within the cantilever edges[13]:

$$\text{RGB luminance} = 0.299R + 0.587G + 0.114B \qquad (16)$$

From the recorded fluorescence images, bulk diffusion properties could also be investigated (Supplementary Fig. 12e). Time-dependent mechanical properties of PEG-DA MW 250, 575, and 700 cantilevers in water were investigated over a 17 min period following initial

hydration (Supplementary Fig. 11). PEG-DA MW 250, 575, and 700 cantilevers require 5, 7, and 7 min at most, respectively, to become fully swollen in water and hence mechanically stabilized. We also wish to note that the cantilevers of the actual hydrogel nano-probes used for AFM applications are several-times thinner than the cantilevers used in the mechanical testing experiments. For the hydrogel AFM nano-probes, they require less than a 1 min incubation time—nearly equivalent to the time needed for the probe to approach the surface. In some experiments, the effect of curing conditions on the elastic modulus of hydrogel cantilevers was investigated by choosing two different UV doses (1.4 and 590 mJ cm^{-2}) for the polymerization of PEG-DA MW 250/575 mixture cantilevers (Supplementary Fig. 14).

Spring constant calibration

Depending on the spring constant estimated from the elastic modulus and geometrical dimensions of the cantilever, a specific method was chosen from three options including the thermal method[14], the reference cantilever method[15], and the stylus profilometer method[16]. For the thermal method, the deflection sensitivity of a hydrogel cantilever was obtained by deflecting the cantilever against a freshly cleaved and atomically smooth mica substrate. With a commercial AFM instrument (NX10, Park Systems), power spectrum densities of the cantilever displacement were measured 100 times, averaged, and fitted to a simple harmonic oscillator model. The area below the fitted curve was integrated and converted to the displacement power by using the deflection sensitivity. Then, the equipartition theorem, $k\langle x^2\rangle = k_b T$, was applied to calculate the spring constant taking into account Boltzmann's constant, k_b, and the ambient temperature, T. For the reference cantilever method, commercial silicon cantilevers underwent thermal calibration as described above and were then used as references. A reference cantilever was configured on the sample mount and a hydrogel cantilever to be calibrated was configured on the cantilever mount of the NX10 AFM instrument. The hydrogel cantilever was pushed against the reference cantilever and the mica substrate up to the deflection setpoint in order to obtain the corresponding deflection sensitivities (S_{ref} and S_{sub}). Then, the spring constant was calculated as follows:

$$k = k_{ref}\left(\frac{l}{l-d_{load}}\right)^3\left(\frac{S_{sub}}{S_{ref}}-1\right)\cos^2\theta \qquad (17)$$

where k_{ref} and l represent the spring constant and length of the reference cantilever, respectively, d_{load} is the distance from the contact point on the reference cantilever to its free end, and θ is the tilt angle of the hydrogel cantilever under calibration[15]. For the stylus profilometer method, a hydrogel cantilever was configured on the sample mount in a stylus profilometer (Dektak XT, Bruker). Then, the stylus in contact with the hydrogel cantilever at a force setpoint longitudinally scanned the hydrogel cantilever. The spring constant was obtained by dividing the static deflection of the hydrogel cantilever by the actual force setpoint from calibration. Hydrogel cantilever types #1, #2~#4, and #5 in Fig. 3h were calibrated by using the stylus profilometer method, the reference cantilever method, and the thermal method, respectively. Commercial silicon cantilevers used as references were OCTO500D for type #2, OCTO1000D for type #3, and OCTO500S for type #4, respectively, all from Micromotives. The hydrogel cantilever #6 was too soft to be calibrated with any of the three methods. Supplementary Table 1 summarizes the calibration methods for determining the spring constant of hydrogel cantilevers used in this work.

Combining the compositional and geometrical tuning capabilities, the spring constant of hydrogel cantilevers can be varied over more than 8 orders of magnitude and this range has been experimentally demonstrated (Fig. 3h and Supplementary Fig. 19). The wide tuning range of the spring constant may extend applications of hydrogel cantilevers beyond AFM nano-probes (i.e. extremely sensitive nanomechanical sensors). In addition, the tuning range can be further extended by introducing other soft or hard hydrogel types as the scheme is general.

Force-displacement (*F-d*) measurements

A hydrogel probe and sample pair was carefully chosen so that the tip elastic modulus exceeded the sample elastic modulus by 40-60 times (40× for PEG-DA MW 575 and 60× for PEG-DA MW 700) for *F-d* measurements in air, and 360-15,000 times (360× for PDMS 10:1 and 15,000× for Polyacrylamide substrates) for *F-d* measurements in water in order to apply the Hertzian model[17,18]. PEG-DA MW 575 and 700 substrates were prepared using the UV LED (590 mJ cm^{-2}). The PDMS pre-polymer mixture was cured on a hot plate at 100 °C for 60 min. Polyacrylamide substrates were prepared under 10 wt vol^{-1}% ammonium persulfate and tetramethylethylenediamine polymerization conditions[6]. For each tip and sample pair, the degree of vertical tip deformation at the tip apex (maximum tip deformation) was quantitatively estimated (Supplementary Table 3). It was shown that the deformations in both the vertical and lateral directions are negligibly small. Because direct measurement of the maximum tip deformation is a challenge, we indirectly measured tip deformation through tip-sample deconvolution analysis. The tip deformation was determined to be ~2.1 nm in the case of contact between the hydrogel tip and a hard silicon substrate with 300 nN contact force (Supplementary Fig. 25). Since the extent of tip deformation would be significantly decreased when the substrate is switched to one with a much lower elastic modulus, it is reasonable to assume that the hydrogel tip deformation is not significant for all tip-sample pairs used in *F-d* measurements. Of note, all samples exhibit much lower elastic moduli than those of hydrogel tips. The lateral tip deformation resulting from the cantilever tilt was also quantitatively analyzed (see Supplementary Discussion for details). It was shown that the maximum lateral deformation of the hydrogel tip is negligibly small for the chosen tip and sample pairs. For these reasons, the hydrogel tip was modeled as a rigid indenter, and the Hertz model was applied to *F-d* measurements performed without any modification. Prior to *F-d* experiments, deflection sensitivities were measured by indenting a freshly cleaved mica substrate with hydrogel probes. Spring constants were calibrated by using the reference cantilever method[5] under the NX10 AFM instrument. Before measuring the spring constants, commercial silicon cantilevers were thermally calibrated and used as reference cantilevers (OCTO500D for type #2, OCTO1000D for type #3, and OCTO500S for type #4, all from Micromotive). PEG-DA and PDMS substrates were treated with soft X-ray (VSN-05UE, VSI) for 20 min before testing to minimize electrostatic interaction between tips and samples. *F-d* measurements were performed at 256 points for all substrates at a loading rate of 1 μm s^{-1} using a pyramid tip-attached hydrogel nano-probe with a tip radius of 150 nm. For measurements in liquid, the hydrogel nano-probe was incubated in water for 10 min beforehand.

Force-displacement data analysis

First, the approach portion of the force-displacement data was converted to the force-indentation data presentation (Supplementary Fig. 15), and the initial 10-20% of *F-d* data was fitted as the baseline which was then subtracted from the *F-d* data. The data after subtraction were divided into two different types depending on the magnitude of adhesive interactions.

For measurements in air, there existed an appreciable adhesive interaction between the PEG-DA MW 250 hydrogel tip and PEGDA MW 575 or 700 substrates. To verify the best-suited model for analysis, the ratio of adhesive force to maximum applied force and the Tabor coefficient were calculated in order to determine which region the experiment belongs to in the adhesion map[19], from which Hertz analysis was invoked[20] (Supplementary Table 2). The contact point was chosen to be the point where the extrapolated baseline intersects with the approach curve (Supplementary Fig. 16a)[20,21]. Then, the data were subtracted from the chosen contact point, and Hertz-fitted while the fitting range was gradually increased (Supplementary Fig. 16b). The fitting range to extract the elastic modulus was determined by locating the range beyond which the extracted elastic modulus becomes insensitive to the change in the fitting range (Supplementary Fig. 16c).

For measurements in liquid where a smooth and continuous transition from the pre-contact to post-contact region was shown (i.e., there was negligible adhesive interaction), the specific range of data was chosen from the point of the apparent pre-contact region to some point in the post-contact region[22] (Supplementary Fig. 17a). The chosen data were fitted with a two-part polynomial equation (18), using a standard Levenberg-Marquardt least squares minimization algorithm[22]:

$$f(\delta) = \begin{cases} a + b\delta & \delta < \delta_{\text{trial}} \\ \frac{4}{3}\sqrt{R}E_{\text{sample}}\left(\delta - \delta_{\text{trial}}\right)^{3/2} + c & \delta > \delta_{\text{trial}} \end{cases} \quad (18)$$

where a, b, and E_{sample} are fitting parameters, and $c = a + b\delta_{\text{trial}}$. A trial contact point, δ_{trial}, was shifted from the very first point of the pre-contact region to the post-contact region with an interval of ~5 nm (Supplementary Fig. 17b) to find the trial contact point that minimizes the root mean squared error. The resulting values of the root mean squared error for the contact point of each trial were plotted in order to locate the contact point that has the best fitting result (Supplementary Fig. 17b). Pre-contact data were discarded, and the remaining post-contact data were Hertz-fitted. The rest of the analysis is the same as measurements conducted in air (Supplementary Fig. 17c, d). The resulting elastic moduli of various soft substrates measured with hydrogel probes both in air and liquid were compared with literature values in Supplementary Table 4.

Durability tests

Durability tests were systematically conducted by investigating the wear characteristics of hydrogel nano-probes with AFM and SEM. First, a hydrogel nano-probe with tip radius of ~30 nm was used to image a hard silicon calibration grating sample (HS-100MG, BudgetSensors) repeatedly in PBS solution with contact mode (force setpoint of 3 nN) to obtain 12 images with the scan size of 40 μm × 40 μm (i.e. cumulative scan distance of 0.246 m). Tip wear was examined by taking a SEM image of the hydrogel tip region without metal coating after obtaining every three AFM images (Supplementary Fig. 33a). We approximated the worn hydrogel tip by a truncated circular cone with a hemispherical cap (Supplementary Fig. 33b) to calculate the wear volume by

$$\Delta V = \frac{\pi}{3}\left(R_i^2 + R_{3n}^2 + R_i R_{3n}\right)(R_{3n} - R_i)\cot\left(\frac{\theta}{2}\right)\cos\left(\frac{\theta}{2}\right) + \pi\left(R_i^3 - R_{3n}^3\right)\left\{\frac{2}{3} + \frac{1}{3}\sin^3\left(\frac{\theta}{2}\right) - \sin\left(\frac{\theta}{2}\right)\right\} \quad (19)$$

where R_i and R_{3n} represent the tip radii before the wear test and after imaging $3n$ frames, respectively, and θ represents the tip cone angle, all of which are extracted from SEM images by using the custom image processing protocol (Supplementary Fig. 4). Supplementary Fig. 33c shows the tip radius and wear volume as a function of the cumulative scan distance.

Durability tests were repeated with different grating materials, imaging modes, and surrounding media. Supplementary Fig. 34 shows SEM images of hydrogel nano-tips taken before, during, and after five durability test cases (i.e. after obtaining 0th, 3rd, 6th, 9th, and 12th AFM images). Among them, SEM images taken before and after durability tests are the same images as shown in Fig. 7a.

Modular assembly of hydrogel nano-probes

Hydrogel cantilevers can be assembled by modules with different compositions. As an example, the hydrogel cantilever structure was made with FITC mixed PEG-DA MW 250 and the tip was made with Rhodamine B mixed PEG-DA MW 575 (Supplementary Fig. 35). This combination would be useful for applications which require selective swelling of the tip region. Arbitrary compositional combinations for the cantilever and tip are feasible. In addition, the cantilever structure itself can be modular if the hydrogel pre-polymer filling and curing are divided into several steps.

Hydrogel nano-probes with functional nanomaterials encapsulated

If the pre-polymer solution is mixed with functional materials, filled, and cured in the tip mold, then the functional materials can be encapsulated within the tip and the hydrogel tip will exhibit and make use of the functionality of the encapsulated materials. In this way, functional hydrogel nano-probes can be realized through established encapsulation schemes. Supplementary Table 5 summarizes the material compositions and fabrication conditions of functional hydrogel nano-probes explored in this work.

Hydrogel nano-probes with integrated magnetic nanoparticles

If magnetic nanoparticles are encapsulated in the hydrogel tip, then magnetically functional hydrogel nano-probes are fabricated. Since the UV curing of hydrogels is highly exothermic and the temperature within the tip mold can be significantly increased during this process, cobalt nanoparticles were chosen due to their relatively high Curie temperature of 1,115 °C (ref. 22) which prevents demagnetization of nanoparticles.

Cobalt nanoparticles (25-30 nm diameter, SkySpring Nanomaterials, Inc.) were first mixed in a PEG-DA MW 250 pre-polymer solution (Cobalt nanoparticles:PEG-DA MW 250 = 20:80, w w^{-1}), and the mixture was dispensed into the pyramid mold and cured by UV exposure (120 mJ cm^{-2}). Then, a tipless hydrogel cantilever was introduced and made contact with the cured hydrogel tip containing cobalt nanoparticles (henceforth termed "magnetic tip"). The magnetic tip was attached to the tipless cantilever upon additional UV exposure (120 mJ cm^{-2}) with the standard tip attachment protocol (Fig. 2b). The magnetic tip responded to an external magnetic field emanating from a permanent neodymium magnet of ~0.38 T. The hydrogel cantilever with the cobalt magnetic tip bent towards and contacted the neodymium magnet when it was placed near the magnet (Supplementary Fig. 36). Therefore, the magnetic tip can be applied to magnetic force microscopy applications. Our primary interest is localized inductive heating with the magnetic tip. To this end, a 1 mm diameter enamel wire was wound to configure a 4.5 cm diameter coil with total 28 turns which exhibited inductance of

70 μH. The drive signal was generated from a function generator (33250A, Agilent) and amplified through a power amplifier (HSA4014, NF Corporation) in order to source powers ranging from 25 to 175 W to the coil. The operating frequency was set to be ~60 kHz which optimizes the efficiency of the induction heating in our current experimental setting (Supplementary Fig. 37). The coil was mounted horizontally such that the magnetic field emanated vertically. When a hydrogel cantilever with the magnetic tip was placed in the middle of the coil under operation, the magnetic tip was inductively heated and the generated heat was transferred to the surrounding medium via convection and to the cantilever beam via conduction. Thermal time constants, τ_{th}, of hydrogel cantilevers were calculated to be ranged from 220 μs to 870 μs for a length of 150~300 μm, width of 50 μm, and thickness of 20 μm with thermal conductivity of 0.25 W m^{-1} K^{-1}, specific heat of 2.2 kJ kg^{-1} °C^{-1}, and density of 1,100 kg m^{-3} (ref. 23). Considering this calculation, any measurement with hydrogel nano-probes with the magnetic tip was started at 10 s after the induction power was turned on (10 s corresponds to 11.5~45.5τ_{th}, thus, thermal equilibrium can be guaranteed)[25]. The temperature rise of the hydrogel cantilever could be indirectly confirmed by the downshift of the resonance frequency, which was acquired after the hydrogel cantilever reached its thermal equilibrium (Fig. 8d and Supplementary Fig. 38). The observed downshift of the resonance frequency is mainly due to the temperature dependent elastic modulus of the cured hydrogel. Resonance spectra of hydrogel nano-probes with the magnetic tip were obtained in air and water with the frequency resolution of ~1.25 Hz and partially fitted with the simple harmonic oscillator model to find out the resonance frequency. For a given power, measurements were repeated three times for statistical analysis.

Hydrogel nano-probes with integrated quantum dots

CdTe quantum dots (PL-QDN-670, PlasmaChem) were first mixed in a PEG-DA MW 700 pre-polymer solution (CdTe quantum dots:PEG-DA MW 700 = 0.0003:0.9997, w w^{-1}), and the mixture was dispensed into the pyramid mold and cured by UV exposure (120 mJ cm^{-2}). Then, a tipless hydrogel cantilever was introduced and made contact with the cured hydrogel tip containing quantum dots (henceforth termed "quantum dot tip"). The quantum dot tip was attached to the tipless cantilever upon additional UV exposure (120 mJ cm^{-2}) with the standard tip attachment protocol (Fig. 2b). The hydrogel probe with the quantum dot tip made contact with the glass side of an indium tin oxide (ITO)-coated glass substrate under Joule heating (Sourcemeter 2400, Keithley) in air and water where the contact force was set at ~200 nN. CdTe quantum dots encapsulated in PEG-DA MW 700 hydrogel tips were stable in water (Supplementary Fig. 40) because their diameters are larger than the mesh size of the UV-cured PEG-DA MW 700 hydrogel (Fig. 8a). Fluorescence spectra of the encapsulated CdTe quantum dots were acquired at various heating powers for the ITO-coated glass by using an optical spectrometer (HR4000, Ocean Optics) with the wavelength resolution of 0.14 nm and partially fitted to the asymmetric double sigmoidal function[26] to obtain peak wavelengths and intensities. Then, the fluorescence intensities were normalized by dividing all acquired values by the peak intensity of the spectrum acquired at room temperature. Once the surface temperature at the tip contact point was independently obtained by using a K-type thermocouple (U1186A, Agilent), the peak wavelength shift and the normalized peak intensity were plotted as functions of the measured temperature (Fig. 8h and Supplementary Fig. 39). After this temperature calibration, the hydrogel probe with the quantum dot-functionalized tip could be used in temperature sensing.

Supplementary Fig. 41 summarizes temperature sensitivities of the fluorescence peak wavelength shift and the normalized peak intensity of various hydrogel probes with quantum dot-functionalized tips in air and water.

Hydrogel nano-probes with co-encapsulated quantum dots and magnetic nanoparticles

CdTe quantum dots (PL-QDN-670, PlasmaChem) were first mixed in a PEG-DA MW 700 pre-polymer solution (CdTe quantum dots:PEG-DA MW 700 = 0.0003:0.9997, w w^{-1}), and the mixture was dispensed into the pyramid mold and cured by UV exposure (120 mJ cm^{-2}). The cured volume of the quantum dot-embedded hydrogel was less than 5% of the total volume of the pyramid mold. Then, cobalt nanoparticles (25-30 nm diameter, SkySpring Nanomaterials, Inc) mixed in a PEG-DA MW 250 pre-polymer solution (Cobalt nanoparticles:PEG-DA MW 250 = 20:80, w w^{-1}), were dispensed into the pyramid mold and cured by UV exposure (120 mJ cm^{-2}). Next, a tipless hydrogel cantilever was introduced and made contact with the dual-functionalized hydrogel tip containing the sequentially embedded quantum dots and magnetic nanoparticles (henceforth termed "dual-functional tip"). The temperature of the hydrogel probe with the dual-functional tip was calibrated by recording the fluorescence spectra of the dual-functional tip in contact with the glass side of the ITO-coated glass substrate under Joule heating in air and water (Supplementary Fig. 42). Following a similar approach to that used to inductively heat hydrogel probes with only cobalt nanoparticles embedded inside, a hydrogel probe with the dual-functional tip was placed in the middle of the coil. When the amplified AC power was applied to the coil, cobalt nanoparticles were inductively heated. The generated heat in the magnetic nanoparticle cluster was transferred directly to the underlying quantum dot cluster via conduction. At a given power, the average temperature of the tip apex region containing quantum dots was measured *in situ* by recording the fluorescence spectrum from the calibrated hydrogel probe with the dual-functional tip in air and water (Fig. 8j and Supplementary Fig. 43). Acquired fluorescence spectra were analyzed by following the same procedures described as described above.

Preliminary experiments for materials delivery using hydrogel nano-probes

For swelling-mediated material delivery applications, a PEG-DA MW 575 pyramidal tip was attached to a hydrogel cantilever made with PEG-DA MW 250. The tip composition was selected on the basis that higher molecular weight PEG-DA hydrogels are more suitable for materials delivery applications due to their larger mesh sizes and greater swelling. After the tip integration, the Rhodamine B dye was encapsulated within the pyramidal tip by dipping the tip in an ink depot[27] containing Rhodamine B-mixed aqueous solutions (1.5, 15, and 150 μM) for 30 s as shown in Supplementary Fig. 44a. A single PEG-DA hydrogel probe can be used for multiple rounds of materials loading and delivery (Fig. 8m). Then, the Rhodamine B-loaded hydrogel tip contacted either a hydrated PEG-DA MW 700 substrate (70% w w^{-1}) at a force of 100 nN at room temperature for 1-10 min (Supplementary Fig. 44b), or MCF-7 cell surface at 37 °C with 1-2 nN for 1 min (Fig. 8n). For sustained delivery, excess water and buffer on PEG-DA substrates and cells was aspirated to leave a thin meniscus of liquid[28]. After local delivery, fluorescence images for PEG-DA substrates and cells were taken with an LV100D fluorescent microscope and the ProgRes® C10^{plus} CCD. The exposure time was set to 100 ms for all images (Fig. 8m,n and Supplementary Fig. 44b).

Supplementary References

1. Bull, M. S., Sullan, R. M. A., Li, H. & Perkins, T. T. Improved single molecule force spectroscopy using micromachined cantilevers. *ACS Nano* **8**, 4984-4995 (2014).
2. Cumpson, P. J., Clifford, C. A. & Hedley, J. Quantitative analytical atomic force microscopy: a cantilever reference device for easy and accurate AFM spring-constant calibration. *Meas. Sci. Tech.* **15**, 1337-1346 (2004).
3. Johnson, B. D., Beebe, D. J. & Crone, W. C. Effects of swelling on the mechanical properties of a pH-sensitive hydrogel for use in microfluidic devices. *Mat. Sci. Eng.: C* **24**, 575-581 (2004).
4. Hopcroft, M. A., Nix, W. D. & Kenny, T. W. What is the Young's Modulus of Silicon? *J. Microelectromech. Syst.* **19**, 229-238 (2010).
5. Wang, Z., Volinsky, A. A. & Gallant, N. D. Crosslinking effect on polydimethylsiloxane elastic modulus measured by custom-built compression instrument. *J. Appl. Poly. Sci.* **131**, 41050 (2014).
6. Tse, J. R. & Engler, A. J. Preparation of hydrogel substrates with tunable mechanical properties. *Curr. Prot. Cell Bio.* **47**, 10.16. 11-10.16. 16 (2010).
7. Johnson, K. L. & Johnson, K. L. *Contact mechanics*. (Cambridge university press, 1987).
8. Timoshenko, S. *History of strength of materials: with a brief account of the history of theory of elasticity and theory of structures*. (Courier Corporation, 1953).
9. Villarrubia, J. Algorithm for scanned probe microscope image simulation, surface reconstruction, and tip estimation. *J. Res. Nat. Natl. Inst. Stan.* **102**, 425-454 (1997).
10. Jones, M. C., Nassimbene, R. D., Wolfe, J. D. & Frederick, N. V. Mixing and dispersion measurements on packed bed flows using a fiberoptic probe array. *Chem. Eng. Sci.* **51**, 1009-1021 (1996).
11. Lo, Y.-S. *et al.* Organic and inorganic contamination on commercial AFM cantilevers. *Langmuir* **15**, 6522-6526 (1999).
12. Otsu, N. A threshold selection method from gray-level histograms. *IEEE Trans. Syst, Man Cybern.* **11**, 23-27 (1975).
13. Sproson, W. N. *Colour science in television and display systems*. (Adam Hilger, 1983).
14. Hutter, J. L. & Bechhoefer, J. Calibration of atomic-force microscope tips. *Rev. Sci. Instrum.* **64**, 1868-1873 (1993).
15. Gates, R. S. & Reitsma, M. G. Precise atomic force microscope cantilever spring constant calibration using a reference cantilever array. *Rev. Sci. Instrum.* **78**, 086101 (2007).
16. Hopcroft, M. *et al.* Micromechanical testing of SU-8 cantilevers. *Fatigue Fract. Eng. Mat. Struct.* **28**, 735-742 (2005).
17. Herruzo, E. T., Perrino, A. P. & Garcia, R. Fast nanomechanical spectroscopy of soft matter. *Nat. Commun.* **5**, 3126 (2014).
18. Tsukruk, V. V., Gorbunov, V. V., Huang, Z. & Chizhik, S. A. Dynamic microprobing of viscoelastic polymer properties. *Polym. Int.* **49**, 441-444 (2000).
19. Johnson, K. L. & Greenwood, J. A. An adhesion map for the contact of elastic spheres. *J. Col. Int. Sci* **192**, 326-333 (1997).
20. Lin, D. C., Dimitriadis, E. K. & Horkay, F. Robust strategies for automated AFM force curve analysis—II: adhesion-influenced indentation of soft, elastic materials. *J. Biomech. Eng.* **129**, 904-912 (2007).
21. Lin, D. C., Dimitriadis, E. K. & Horkay, F. Robust strategies for automated AFM force curve analysis—I. Non-adhesive indentation of soft, inhomogeneous materials. *J. Biomech. Eng.* **129**, 430-440 (2007).
22. T., D. J. & Brooke, T. M. *Cell Imaging Techniques: Methods and Protocols*. (Humana Press Totowa, 2006).
23. Enghag, P. *Encyclopedia of the elements: technical data-history-processing-applications*. (John Wiley & Sons, 2008).
24. DiGuilio, R. & Teja, A. S. Thermal conductivity of poly (ethylene glycols) and their binary mixtures. *J. Chem. Eng. Data* **35**, 117-121 (1990).

25. Chui, B. W. *et al.* Low-stiffness silicon cantilevers with integrated heaters and piezoresistive sensors for high-density AFM thermomechanical data storage. *J. Microelectromech. Syst.* **7**, 69-78 (1998).
26. Khan, T., Bodrogi, P., Vinh, Q. T. & Winkler, H. *LED Lighting: Technology and Perception*. (John Wiley & Sons, 2014).
27. Bog, U. *et al.* On-chip microlasers for biomolecular detection via highly localized deposition of a multifunctional phospholipid ink. *Lab Chip* **13**, 2701-2707 (2013).
28. Loh, O. *et al.* Nanofountain-Probe-Based High-Resolution Patterning and Single-Cell Injection of Functionalized Nanodiamonds. *Small* **5**, 1667-1674 (2009).
